# The Applicability and Performance of Tools Used to Assess the Father-Offspring Relationship in Relation to Parental Psychopathology and Offspring Outcomes

**DOI:** 10.3389/fpsyt.2020.596857

**Published:** 2021-01-05

**Authors:** Jasmine Siew, Jane Iles, Jill Domoney, Florence Bristow, Zoe J. Darwin, Vaheshta Sethna

**Affiliations:** ^1^Department of Forensic and Neurodevelopmental Sciences, Institute of Psychiatry, Psychology and Neuroscience, King's College London, London, United Kingdom; ^2^Department of Psychology, University of Surrey, Guildford, United Kingdom; ^3^Section of Women's Mental Health, Health Service and Population Research Department, Institute of Psychiatry, Psychology and Neuroscience, King's College London, London, United Kingdom; ^4^Perinatal Services for Croydon, South London and Maudsley National Health Service (NHS) Foundation Trust, London, United Kingdom; ^5^School of Human and Health Sciences, University of Huddersfield, Huddersfield, United Kingdom

**Keywords:** partners, paternal depression, perinatal mental health, fathers, paternal involvement, father-offspring relationship quality

## Abstract

**Introduction:** Father-infant interactions are important for optimal offspring outcomes. Moreover, paternal perinatal psychopathology is associated with psychological and developmental disturbances in the offspring, and this risk may increase when both parents are unwell. While, the father-offspring relationship is a plausible mechanism of risk transmission, there is presently no “gold standard” tool for assessing the father-offspring relationship. Therefore, we systematically searched and reviewed the application and performance of tools used to assess the father-offspring relationship from pregnancy to 24-months postnatal.

**Methods:** Four electronic databases (including MEDLINE, PsycINFO, Maternity and Infant Care Database, and CINAHL) were searched. Selected articles included evidence of father-offspring relationship assessment in relation to parental perinatal psychopathology and/or offspring outcomes. Data was extracted and synthesized according to the following: (i) evidence supporting the performance of tools in terms of their psychometric properties when applied in the context of fathers, (ii) tool specific characteristics, and (iii) study specific methodological aspects in which the tool was embedded.

**Results:** Of the 30,500 records eligible for screening, 38 unique tools used to assess the father-offspring relationship were identified, from 61 studies. Ten tools were employed in the context of paternal psychopathology, three in the context of maternal psychopathology, and seven in the context of both maternal and paternal psychopathology, while nine tools were applied in the context of offspring outcomes only. The remaining nine tools were used in the context of both parental psychopathology (i.e., paternal, and/or maternal psychopathology) and offspring outcomes. Evidence supporting the psychometric robustness of the extracted observational, self-report and interview-based tools was generally limited. Most tools were originally developed in maternal samples—with few tools demonstrating evidence of content validation specific to fathers. Furthermore, various elements influencing tool performance were recognized—including variation in tool characteristics (e.g., relationship dimensions assessed, assessment mode, and scoring formats) and study specific methodological aspects, (e.g., setting and study design, sample characteristics, timing and nature of parental psychopathology, and offspring outcomes).

**Conclusion:** Given the strengths and limitations of each mode of assessment, future studies may benefit from a multimethod approach to assessing the father-offspring relationship, which may provide a more accurate assessment than one method alone.

## Introduction

The antenatal period and 1st year postpartum (“perinatal period” hereafter) are associated with significant adjustments to fathers that pose risks for mental health difficulties. Among the various disorders that manifest perinatally, depression is one of the most common with incidence rates ranging between 8.4 and 10% in fathers (1, 2)—this risk increasing more than two-fold in the context of maternal depression (3, 4). Moreover, psychopathology in fathers is associated with a range of psychological and developmental disturbances in the offspring (5), with increased vulnerability when both parents experience mental health difficulties in the perinatal period (2, 3, 6). Although genetic and environmental vulnerabilities may contribute to adverse offspring outcomes, a key potential mechanism of risk transmission is parenting quality (5).

There is emerging evidence in support of each link of this mechanism—i.e., between paternal perinatal mental health disorders and the father-offspring relationship, as well as between the father-infant relationship and subsequent offspring development. In general, less optimal parenting and reduced paternal involvement have been reported in the context of both diagnostic and symptom-level difficulties, experienced by fathers, in the perinatal period [for a review, see (7)]. Comparably, non-optimal patterns of parenting, associated with the occurrence of paternal perinatal psychopathology, may also explain adverse offspring outcomes (8).

Furthermore, perinatal psychopathology experienced by the mother also influences paternal parenting (9)—with evidence in support of both the “*spill over”* hypothesis (whereby higher depressive symptom-levels are associated with less optimal father-offspring relationships); as well as the “*compensatory/buffering”* model (such that fathers may try to compensate for (or buffer against) depression experienced by their partner by becoming more involved with the infant) (10).

Yet, inconsistencies in the reported evidence exist, with studies reporting no significant links between father-offspring relationship indices and either paternal or maternal psychopathology during the antenatal period [e.g., (11)], the 1st year postpartum [e.g., (12, 13)], and up to 24-months postpartum [e.g., (14)]; as well as with infant outcome domains [e.g., (15–17)].

Nevertheless, difficulties in parenting that tend to be especially evident in the first postnatal year (18–20) manifest in many different forms. They may become apparent through the *quality* of the father-infant relationship—such as fathers attachment relationship with the fetus [e.g., (21, 22)] behavioral disengagement and expression of negative affectivity [e.g., (23–25)], fewer mental state references in speech (26)—as well as *quantity* of paternal involvement—indexed by lower levels of engagement in positive enrichment activities and routine child-care [e.g., (10, 27, 28)]. In response infants may also alter their interactive behaviors with increased negative affect and avoidance, such as gaze aversion (23). Thus, infancy is a key developmental phase in the intergenerational psychopathology transmission by impairments in the father-infant relationship. Consequently, an array of tools has been used to assess parenting behaviors of fathers (typically observational and self-report assessments, but also interview schedules).

In addition to studying the quality and quantity of social interactions between parent and child, attachment security is another widely assessed index of the quality of the parent-child relationship. The Strange Situation Procedure [SSP: (29)] is the gold standard for classifying infants into one of three organized attachment patterns, identified by Ainsworth and colleagues, and a fourth the disorganized-disoriented (D) pattern introduced subsequently by Main and Solomon (30). Yet, there is currently no “gold-standard” tool for assessing the interplay between father and infant in the perinatal period. Different modes of assessments for the father-offspring relationship have their own strengths and weakness, and it is likely that to some degree, the discrepancies in findings across studies may be attributable to the measurement method, rather than the specific construct being measured (31). For example, while observational tools permit the objective assessment of parent, infant and dyadic behaviors, they are time-consuming, are often restricted in terms of the duration of observation (typically ~5-min) and behaviors may be influenced by specific demand characteristics of the situation (e.g., videotaping, presence of an observer, structured settings) (32). On the other hand, self-report tools are easy to administer, require minimal training and can be applied to large samples; however, respondents may be influenced by response bias, including social desirability (33).

Also, certain parenting dimensions may be better captured by a specific assessment tool. In particular, the coding of affect expressions and emotion during observed interactions are often difficult to assess since the coding of these dimensions often relies on inferences from the observer and may be judged in an unsystematic manner. However, self-report and interview tools may be especially useful for capturing subjective emotional information—though, still subject to certain biases (34, 35). Taken together, the selection of a tool requires extensive knowledge about the dimensions of parenting that one intends to assess, considering the strengths and weaknesses of each assessment method. Equally important, is knowledge on the tool's psychometric performance. Hence, a systematic summary of tools, to assess the father-offspring relationship, as well as an evaluation of their psychometric properties in the context of parental psychopathology and/or offspring outcomes (key variables in the parenting model of risk transmission), is warranted.

While existing reviews have examined the father-offspring relationship in the context of parental psychopathology [for example, (7, 36)] and in relation to child developmental outcomes [for example, (36–39)], few have focused on the application and performance of the tools used to assess the father-offspring relationship in both these contexts. Those reviews which have included some evaulation on how tools have been applied to assess the father-offspring relationship have primairly focused on one type of assessment tool (mostly observational or self-report measures of the father-offspring relationship) [for example, (32)] and/or have examined a particular construct of parenting behavior (for example, paternal play, attachment relationship, paternal involvement) [for example, (37, 40, 41)]. However, given the strengths and limitations of each mode of assessment and their potentially differential performance when applied to specific features of the father-offspring relationship, a multi-method approach to the evaulation of available tools would be beneficial in helping guide tool selection. Lastly, although existing reviews have evaluated the psychometric properties of some tools developed in father-only samples (32, 40, 41)—though still focused on one type of measurement tool or a specific parenting construct—clarity is needed to also understand how tools developed in maternal and/or parental samples perform when applied to assess various behavioral features of the father-offspring relationship (32). This is important since mothers and fathers may differ in the way they understand and interact with their child (37), despite evidence of similarities in their early parenting behaviors.

Therefore, this study aims to review the application and performance of tools used to assess the father-offspring relationship when examined in the context of paternal and maternal psychopathology and/or offspring outcomes, during the perinatal period and up to 24-months postpartum. First, we focus on tools applied to assess two key domains of the father-offspring relationship: father-offspring relationship quality and paternal involvement. Second, we focus on both the antenatal and postnatal timepoints for the measurement of the father-offspring relationship. The antenatal time-point is a period of considerable vulnerability for the father-offspring relationship—that continues across early infancy. Also, parenting sensitivity is thought to originate in the antenatal period (42–44) and antenatal mental representations of bonding with the unborn child likely influence early postnatal parenting behavior (22, 45). Similarly, increased paternal involvement during the antenatal period is linked with both positive fetal outcomes [for a review, see (46)], and improved parenting (47, 48). Third, we examine tools used to assess paternal, infant and dyadic patterns of behavior since the father-offspring relationship is not only shaped by parent behavior, but also the degree of coordination with the infants cues and signals (49). Finally, we assess the performance of tools in the context of wider family characteristics—since they are likely to shape the developing father-offspring relationship (36, 50), and also independently influence both parental mental health and offspring outcomes (51, 52).

Utilizing a systematic search and review framework (53) the study objectives were: (i) to review evidence supporting the performance of tools (applied to assess father-offspring relationship quality and paternal involvement) in terms of their psychometric properties, (ii) to evaluate specific characteristics of each tool, and (iii) study specific methodological aspects in which the tool was embedded.

## Methods

### Father-Offspring Relationship: Definition

Two key relationship domains comprise:

1. Father-offspring relationship quality—covering: (i) direct father-offspring interactions assessing parent and/or infant interactive behaviors (often, but not restricted to, recorded face-to-face interactions), and (ii) father-offspring attachment relationship—i.e., the fathers' antenatal relationship to the fetus and postnatal bonding with the infant.
2. Involvement—fathers' engagement in a range of child-care related activities (including, for example, direct engagement in positive activities such as reading, singing, playing, and engagement in routine activities (i.e., bathing, feeding, dressing) (54).

### Search Strategy

A systematic search was conducted using four electronic databases to identify relevant articles from inception to June 2020 (MEDLINE, PsycINFO, Maternity and Infant Care Database, and CINAHL). A combination of search terms was used: fathers or partners; father-child or parent-child relations; generic terms for mental health; and antenatal or postpartum period (see Supplementary Data Sheet 1 for an example of the search strategy).

The search was intentionally broad to identify relevant studies for a series of reviews to support a best practice guide (55). Subsequently, the performance of the search strategy, for each separate review, was tested using key papers and refined to improve sensitivity.

The electronic databases search was complemented by backward and forward citation chaining—i.e., respectively checking reference lists within included studies and checking subsequent studies that cited these. In preparation for publication, forward chaining was used to check for any relevant papers published since the initial search and complimentary second search. Records were imported into referencing software (Endnote version X9) and duplicates removed.

Finally, relevant tools identified in the included studies were subject to citation chaining—i.e., checking studies which cited the original source of the extracted relationship tool. Thus, the original source of the tool was located and additional studies using the same tool to assess the father-offspring relationship were checked (subject to the same eligibility criteria).

### Study Selection

Records screened based on title and abstract, by a pool of reviewers, were categorized as: selected for full-text review, discuss, exclude. Records were selected for full-text review, if within the abstract, there was evidence of father-offspring relationship assessment in relation to parental perinatal psychopathology and/or offspring outcomes. Where there was uncertainty, further discussions occurred. A second reviewer then confirmed categorisations, and where necessary, obtained potentially eligible studies in full. Studies confirmed for full-text review were appraised against the eligibility criteria and then confirmed by a second reviewer. Conflicting viewpoints were discussed among members of the review panel until consensus for inclusion or exclusion was reached.

### Study Eligibility Criteria

Inclusion criteria were: (i) *study design:* observational studies; (ii) *sample:* expectant or new fathers or partners (e.g., step-fathers) recruited from clinical or community settings; (iii) *tools*: included at least one tool used to assess the relationship between the father and the offspring not confined to, but taking place at some point during the antenatal period and until 24-months postpartum; (iv) *tool's mode of administration*: observational, self-report or interview schedules; (v) *tool administration*: professional, researcher, and self-administered, and (vi) *tool's correlates*: a range of parental psychopathology (links between paternal and/or maternal psychopathology and the father-offspring relationship) and/or offspring outcomes (linking the father-offspring relationship with independent offspring outcomes).

Exclusion criteria were: (i) tools which did not include the measurement of paternal interactive features (e.g., tools that focused on infant attachment security toward the parent); (ii) interactions with twins/triplets, or triadic interactions; (iii) self-report or interview measures of the father-offspring relationship completed on behalf of the father by the mother; (iv) full-text articles not written in the English language, and (v) studies not published in peer-reviewed journals.

### Data Extraction

#### Data Extraction of Included Studies and Father-Offspring Interaction Tools Identified

Data were extracted and tabulated according to the study descriptive characteristics and key features of included tool(s) used to assess the father-offspring relationship. However, where details relating to the tool's characteristics were not reported in the included studies, these data were extracted at the tool's source.

#### Data Extraction for the Tool's Psychometric Properties

Data supporting evidence of the tool's validity were guided by the New Standards for Educational and Psychological Testing (56).

The framework comprised four key validity domains—i.e., internal structure, content validity, response process, and relations with other variables.

Table 1 includes an overview of descriptions and methods of evaluation for each validity domain. A second reviewer confirmed that the extracted data supported evidence for validity. Conflicting viewpoints were discussed until consensus was reached.

**Table 1:** Descriptions of validity domains and methods of evaluation.

**Validity domain^a^**	**Descriptions of validity domain^b^**	**Methods of evaluating validity domain^b^**
1. Internal structure validity	The extent to which relations between internal components and scores correspond with the definition and intended structure of the construct being measured (i.e., cohesiveness of items and domains).	• Inter-scale correlations among items and/or domains • Cronbach Alpha • Factor analysis • Inter-intra rater reliability • Test-retest reliability
2. Content validity	The extent to which the content of a measure represents a specified content domain—for the purpose of this review, we focus on content specific to the father-offspring relationship.	• *Theory*: content is based on theoretical evidence • *Expert review:* content of tool reviewed by experts • *Content analysis*: interviews with the target group subject to content analyses
3. Response process validity	The extent to which respondents or observed individuals and raters understand the construct and perform in the same way corresponding to the intended, defined construct being measured.	• *Feedback:* interview or observe respondents, observed individuals or raters on their performance of the measure—for example, through piloting of the measure
**4. Relations with other variables (3 sub-domains)**		
*4a. Convergent validity*	The presence and strength of an association between a measurement construct and another measure of the same construct, or related constructs—for the purpose of this review, the same construct refers to measures of paternal parenting, while related constructs refer to measures of maternal parenting and severity of parental psychological symptoms	• Presence (*p* < 0.05) and strength of association (i.e., effect sizes pertaining to weak, moderate, and strong associations between two measurements)^c^.
*4b. Discriminant validity (known groups)*	The extent to which differences in scores between groups reflect known differences in the level of the construct—for the purpose of this review, we focus on clinical diagnostic groups and parent symptom level groups	• Significant differences (*p* < 0.05) between two groups (e.g., clinically depressed vs. non-depressed fathers).
*4c. Criterion validity*	The extent to which a measure is correlated with an external criterion variable, simultaneously (concurrent) or at a point in the future (predictive)—for the purpose of this review, we focus on offspring outcomes for the external criterion variable	• Presence (*p* < 0.05) and strength of association (i.e., effect sizes pertaining to weak, moderate, and strong associations between two measurements)^c^.

a *Validity domains are according to The Standards for Educational and Psychological Testing (56)*.

b *Additional guidance for definitions and methods of evaluation by two external sources (Goodwin and Leech, 2003; Krabbe, 2016)*.

c *Effect sizes: Cohen's r—including weak (<0.3), moderate (0.03), and large effects (>0.5) (57); Cohen's d – including weak (<0.2), moderate (0.5), and large effects (0.8) (57); Odds Ratio (OR) effect size conversion—including weak (1.68), moderate (3.47), and large effects (6.71) (58)*.

### Data Synthesis

The data extracted were synthesized and presented as follows: (1) study specific methodological aspects in which the tool was embedded (i.e., study setting, sample characteristics, sample size and relations with diverse psychopathological and offspring outcome variables); (2) the tool's characteristics (i.e., the conceptualization of father-offspring relationship constructs, interaction dimensions, as well as their scoring formats); (3) evidence supporting the performance of tools in terms of their psychometric properties.

## Results

### Sample

**Figure 1:**
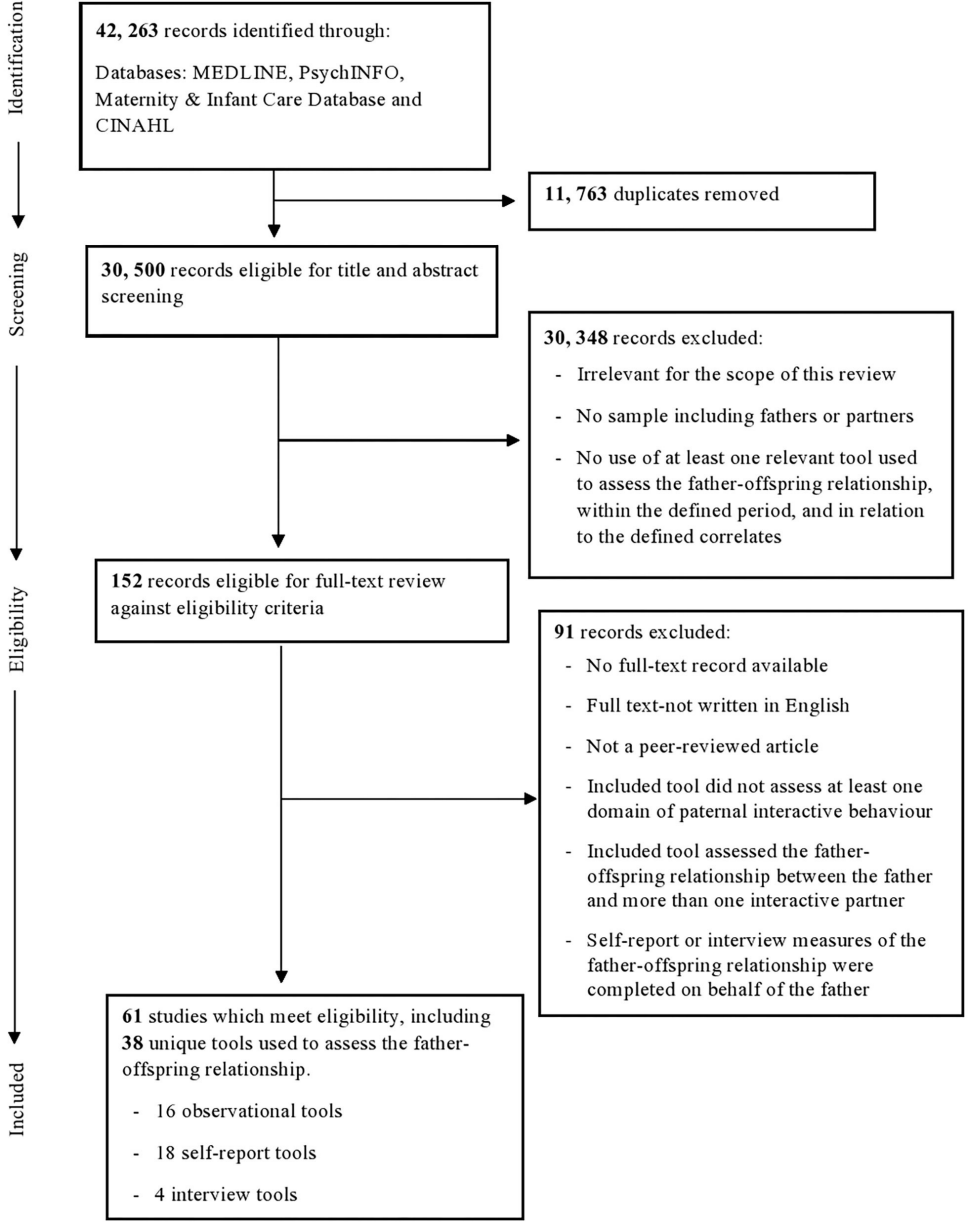
Flowchart of review process.

Forty-two thousand two hundred sixty-three publications were identified. After removing duplicates, 30,500 records were eligible for screening. From this sample, 30,348 publications were excluded by screening titles, and where necessary abstracts. As shown in Figure 1, this process led to the identification of 152 records eligible for full-text review, 91 of which were excluded when reviewed in full. Thus, the final sample comprised 61 publications.

### Descriptive Characteristics of Publications

#### Identification of Publications

The 61 included publications presented relevant data concerning 38 unique tools. Ten tools were employed in the context of paternal psychopathology, three in the context of maternal psychopathology, and seven in the context of both maternal and paternal psychopathology. While nine tools were applied in the context of offspring outcomes only; the remaining nine were used in the context of both parental psychopathology (i.e., paternal, and/or maternal psychopathology) and offspring outcomes.

Twenty-six tools were used to assess father-offspring relationship quality, while 12 were used to assess paternal involvement. Almost half of the tools included in this review were developed in samples comprising both mothers and fathers (*n* =18): five observational, nine self-report, and four interview tools. In contrast, only seven tools were developed in father-only samples: two observational and five self-report measures. The remaining 13 tools were developed in maternal samples only: nine observational and four self-report tools.

#### Study Country of Origin and Data Analyses Time-Points

Studies were published across 14 different countries from 1989 to 2019: countries in Europe (*n* = 25), North America (*n* = 24), Australia (*n* = 5), Asia (*n* = 3), South America (*n* = 1), and the Middle East region (*n* = 3). Cross-sectional data points were extracted from 34 studies and longitudinal data points were extracted from 17 studies, while 10 studies included both cross-sectional and longitudinal data points. Across most longitudinal data points extracted, the follow-up period was generally within an 18-months window ranging from 2-to-18 months—with an average follow-up period of ~6-to-9-months.

#### Sample Characteristics: Clinical Characteristics and Paternal Socio-Demographics

All studies involved community samples of fathers. Forty of the 61 studies included a measure of parental psychopathology. Thirty-five studies assessed paternal psychopathology—of which 28 studies screened fathers for psychological symptoms, four provided clinical diagnostic categorization and three studies reported both. Within these 35 studies, 11 also included a sample of mothers screened for maternal psychological symptoms, while two studies measured both symptoms and diagnostic categorization in mothers. An additional four studies assessed maternal psychological symptoms in the absence of a paternal measure of psychopathology, while one study provided diagnostic categorization in mothers only.

Study samples were largely homogeneous—comprising mainly white European fathers from middle to high-income settings, residing in the family home and from two-parent families. Fathers' education level was primarily assessed through either the average number of education years, or level of education attainment—with years in education ranging from 12- to-16-years or the completion of college education or above (across the majority of studies). Moreover, fathers' age at first measurement of the father-offspring relationship ranged from 18-to-51 years—with a mean age of 34 years.

Supplementary Files 1–3 provide a summary of descriptive characteristics of studies utilizing observational, self-report and interview tools, respectively. Additional details extracted are presented in Supplementary Files 4–6 (59–82).

### Descriptive Characteristics of Tools Used to Assess the Father-Offspring Relationship Across the Included Studies

#### Observational Tools: Set-Up, Duration, Scoring, and Interactive Behaviors of Father-Infant Relationship Quality Assessed

The 31 studies utilizing observational tools (*n* = 16) were all based on face-to-face interactions between father-infant dyads conducted in the family home (*n* = 24) or a laboratory setting (*n* = 7). Interactions were conducted using a range of settings—i.e., free play on a floor-mat (*n* = 11), an infant-seat setting (*n* = 5), structured play (*n* = 6), feeding interactions (*n* = 2), or a combination of these settings (*n* = 7). Most interactions included the use of toys—only six explicitly excluded the use of toys during interactions. The duration of face-to-face observational assessments were mostly between 3 and 10 min. Moreover, there were a range of scoring formats across the observational tools—i.e., rating scales (*n* = 8), frequency counts (*n* = 3); time-durations (*n* = 1) and binary scales (*n* = 1), or a combination of these scoring formats (*n* = 3).

All the observational tools included in this review assessed one interaction domain—i.e., the father-infant relationship quality. These tools assessed at least one of the following features: (i) *paternal interactive behavior* (*n* = 11) (e.g., sensitive, controlling, intrusive, and remote behaviors), (ii) *paternal affect* (*n* = 6) (e.g., overall displays of positive or negative interactive affect or mood) and/or (iii) *paternal speech analysis* (*n* = 3) (e.g., mentalization, attentional focus of speech).

In addition to paternal behaviors, infant and dyadic behaviors were also assessed. Five tools assessed infant behavior—i.e., (i) *infant interactive behavior* (e.g., passivity, responsiveness toward the father) and (ii) *infant affect*; while four tools assessed dyadic behavior—i.e., (iii) *dyadic interactive behavior* (e.g., dyadic synchrony), and (iv) *dyadic affect*.

Finally, three tools extracted from three studies assessed the overall quality of the father-offspring relationship (including paternal and/or infant behaviors).

#### Self-Report Tools: Responder, Items, Time to Complete, Features of Father-Offspring Relationship Quality, and Paternal Involvement Assessed

The number of items across self-report tools ranged from 8-to-24. Most of the tools used rating scales—ranging from 4-to-14 points. None of the studies reported the time to complete self-report assessments.

Self-report tools assessed two domains of the father-offspring relationship: (i) father-offspring relationship quality and (ii) paternal involvement. Father-offspring relationship quality was assessed by ni of the 18 self-report tools. Four features of relationship quality were identified across these tools: (i) *fathers attachment relationship to the fetus* (*n* = 3), (ii) *fathers attachment relationship to the infant* (*n* = 3) (iii) *father-infant bonding* (*n* = 1), and (iv) *paternal warmth* (*n* = 2). Paternal involvement in child-care related activities was assessed by the remaining nine self-report tools.

#### Interview Tools: Responder, Set-Up, Duration, Features of Paternal Involvement, and Father-Offspring Relationship Quality Assessed

Interviews were conducted with fathers in the family home (*n* = 2) or over the telephone (*n* = 1); while one tool was used in the context of both face-to-face and phone interviewing. Only one study (83) reported the interview duration (45 min; The Working Model of Child Interview [WMCI; (84)]. Moreover, scoring formats comprised a rating scale (*n* = 1), time-durations (*n* = 2), or a combination of the two (*n* = 1).

The four interview tools assessed two domains of the father-infant relationship: (i) father-infant relationship quality and (ii) paternal involvement. Father-infant relationship quality was assessed by one tool and paternal involvement in child-care was assessed by the remaining three interview tools.

### Validity of Tools Used to Assess the Father-Offspring Relationship

Data supporting evidence of the tool's validity is presented below (i.e., evidence indicated as the presence of an association at *p* < 0.05 across the validity domains). Table 2 provides a summary of the evidence supporting the psychometric robustness of observational, self-report and interview tools across four validity domains.

**Table 2:** Summary of the tools used to assess the father-offspring relationship demonstrating evidence of validity, extracted from included studies.

	**Internal structure**				**Content validity**			**Response process**	**Relations with other variables**					
									**Convergent validity**			**Discriminant validity**		**Criterion validity**
	**IRR / internal consis-tency**	**Inter- scale corre-lations**	**Factor analysis**	**Test-retest reliability**	**Theory driven**	**Expert review**	**Content analysis**	**Feed-back**	**Paternal parenting measure**	**Maternal parenting measure**	**Severity of paternal/ maternal symptoms**	**Paternal/ maternal diagnostic group**	**Paternal/ maternal symptom level groups**	**Concurrent/ predictive**
**Observational tools developed in maternal samples (*****n*** **=** **9)**														
AMSS	1	0	0	0	N/A	N/A	N/A	N/A	0	0	0	0	0	0
Assessment of Mind-Mindedness	3	2	0	0	N/A	N/A	N/A	N/A	0	0	0	0	0	P3
Behavior-State System	1	0	0	0	N/A	N/A	N/A	N/A	0	0	0	0	0	0
Categorical System for Micro-Analysis of the Early Mother–Child Interaction	1	0	1	0	N/A	N/A	N/A	N/A	0	0	0	0	0	P1
CARE-Index	2	0	0	0	N/A	N/A	N/A	N/A	0	2	PS1, MS1	0	0	P1
Competing demands task (85)	1	0	0	0	N/A	N/A	N/A	N/A	0	0	0	0	0	0
GRS	4	2	0	0	N/A	N/A	N/A	N/A	0	2	0	PDG1	0	P1, C1
NICHD	6	1	0	2	N/A	N/A	N/A	N/A	1	3	0	0	0	P2, C1
SVIA	1	0	0	0	N/A	N/A	N/A	N/A	0	0	PS1	0	0	0
**Observational tools developed in paternal (*****n*** **=** **2) and parental samples (*****n*** **=** **5)**														
EAS	1	0	0	0	0	0	0	0	0	0	0	0	0	0
NCATS	2	0	0	0	0	0	0	0	0	0	PS1, MS1	0	MSG1	P1
PCERA	3	1	2	0	0	0	0	0	0	1	PS2, MS1	PDG1	MSG1	0
PCAMS^f^	1	0	0	0	1	0	0	0	0	0	0	PDG1	0	0
P-PATS^f^	1	0	0	0	1	0	0	0	0	0	0	PDG1	0	0
Unnamed (49)	1	0	0	0	0	0	0	0	0	1	0	0	0	0
Unnamed tool (86)	1	0	0	0	0	0	0	0	0	0	0	0	0	0
**Self-report tools developed in maternal samples (*****n*** **=** **4)**														
CRQ	1	0	0	1	N/A	N/A	N/A	N/A	0	0	0	0	0	0
FAB	1	0	0	0	N/A	N/A	N/A	N/A	1	0	PS1	0	0	0
PAQ	0	0	0	0	N/A	N/A	N/A	N/A	0	0	PS1	0	0	0
PBQ	0	1	0	1	N/A	N/A	N/A	N/A	0	1	PS2, MS1	0	0	C1
**Self-report tools developed in parental (*****n*** **=** **9) and paternal samples (*****n*** **=** **5)**														
Child-Rearing Practices Report [CRPR; (87)]	1	1	1	1	0	0	0	0	0	0	PS1	0	0	P1
HOME-SF	1	0	0	0	0	0	0	0	0	0	PS1	0	0	0
K-PAFAS^f^	1	1	1	1	1	1	1	1	1	0	PS1	0	0	0
Parental Involvement Questionnaire	2	0	0	2	0	0	0	0	0	2	0	0	0	P2
PAAS^f^	5	2	0	0	1	1	1	1	2	2	PS3, MS1	0	0	0
PFAS^f^	1	0	1	0	1	0	0	0	0	1	0	0	0	0
PI	1	0	0	1	1	0	0	0	1	0	0	0	0	0
PPAQ^f^ (published and unpublished versions)	5	1	1	2	1	1	1	1	0	2	PS3	0	PSG1	C2, P1
PRS	2	0	1	0	0	0	0	0	0	0	0	0	0	C1
Unnamed tool (88)	1	0	0	1	0	0	0	0	1	0	0	0	0	0
Unnamed tool (10)	1	0	0	1	0	0	0	0	1	0	0	0	0	0
Unnamed tool^f^ (17)	0	0	0	1	1	0	0	0	0	0	0	0	0	P1
Unnamed tool (28)	0	0	1	0	0	0	0	0	0	0	PS1	0	PSG1, MSG1	0
Unnamed measure (89)	0	0	0	0	0	0	0	0	0	0	0	0	0	C1
**Interview tools developed in parental samples (*****n*** **=** **4)**														
CDS	0	0	0	0	0	0	0	0	0	0	MS1	PDG1	0	0
Parental Involvement Time Diary (27)	0	0	0	0	0	0	0	0	0	0	PS1, MS1	0	0	0
WMCI	1	0	0	0	0	0	0	0	1	0	0	0	0	0
Unnamed tool (90)	0	0	0	0	1	0	0	0	0	0	0	0	0	0

f *, tool developed in father-only samples; PS/MS, evidence of convergence with the severity of paternal/maternal symptoms; PDG/MDG, evidence of discrimination between paternal/maternal diagnostic groups; PSG/MSG, evidence of discrimination between paternal/maternal symptom level groups; C/P, evidence of concurrent/predictive criterion validity*.

*Tool citations—Observational tools: Ainsworth Maternal Sensitivity Scales [AMSS; (91)]; Assessment of Mind-Mindedness (92); Behavior-State System (93); Categorical System for Micro-Analysis of the Early Mother–Child Interaction (94); CARE-Index [infant form; (95)]; Competing demands task (85); Global Rating Scales [GRS; (96, 97)]; National Institute of Child Health and Human Development coding scales [NICHD (98)]; Scale for Mother-Infant Interactions during Feeding [SVIA; (99)]; Emotional Availability Scales [EAS; (100)]; Nursing Child Assessment Teaching Scales [NCATS; (101)]; Parent Child Early Relational Assessment Scale [PCERA; (102)]; Paternal Cognitive Attributional Mentalizing Scale [PCAMS; (26)]; Paternal-Physicality, Affect and Touch Scale [P-PATS; (25); Unnamed (49); Unnamed tool (86)]; self-report tools: Child-Rearing Practices Report [CRPR; (87)]; Child Rearing Questionnaire [CRQ; (103)]; How I Feel About my Baby Now [FAB, (42)]; Home Observation for Measurement of the Environment—short-form version [HOME-SF; (104)]; Parental Attachment Questionnaire [PAQ; (105)]; Postpartum Bonding Questionnaire [PBQ; (106)]; Korean Paternal-Fetal Attachment Scale [K-PAFAS; (107)]; Parental Involvement Questionnaire (108); Paternal Antenatal Attachment Scale [PAAS; (109)]; Paternal Fetal Attachment Scale [PFAS; (110)]; Paternal Involvement Scale [PI; (111)]; Paternal Postnatal Attachment Questionnaire [PPAQ; (112)]; Parental Responsibility Scale [PRS; (113)]; five unnamed tools (10, 17, 28, 88, 89); interview tools: Child Development Supplement to Panel Study of Income Dynamics Time Diary [CDS; (114)]; Parental Involvement Time Diary (27); Working Model of Child Interview [WMCI; (84)]; an unnamed tool (90)*.

Further details extracted are available in Supplementary Files 7–9. This includes data from other father-infant interaction domains assessed by the included tools in this review which showed non-significant associations when examined in relation to psychological symptoms, offspring outcomes and other parenting measures—this data did not support the tools validity argument.

#### Validity Evidence Supporting Observational Tools

All 16 observational tools used to assess father-infant relationship quality demonstrated evidence of validity in at least one domain (i.e., internal structure, content validity, response process and relations with other variables), extracted from all of the included studies using observational tools in this review.

##### Internal Structure Validity—Observational Tools

All 16 observational tools demonstrated evidence of internal structure primarily through the measurement of inter-rater reliability using intraclass correlation coefficients (ICC) or Cohen's kappa coefficients ()—with authors typically establishing moderate to good levels of agreement between raters, when applied to paternal samples in the included studies.

Moreover, five of the 16 observational tools demonstrated additional evidence of internal structure in the remaining three sub-domains. This was demonstrated through evidence of dimensional structure with inter-scale correlations (i.e., Assessment of Mind-Mindedness; GRS; NICHD; PCERA), factorial validity (i.e., Categorical System for Micro-Analysis of the Early Mother–Child Interaction, PCERA) and test re-test reliability (i.e., NICHD).

##### Content Validity—Observational Tools

Two of the seven observational tools developed in paternal and parental samples (PCAMS; P-PATS) demonstrated evidence of content validity—through theoretically driven items specific to the measurement of paternal parenting. However, none of the observational tools demonstrated evidence of content validity, through the expert review of interactive domains or item development supported through interviews with fathers subject to content analysis.

##### Response Process Validity—Observational Tools

There was no reported evidence of response process validation across any of the observational tools developed in parental or paternal samples, included in this review.

##### Relations With Other Variables (i.e., Convergent, Discriminant, Criterion Validity)—Observational Tools

The majority of observational tools (12/16) excluding the AMSS, Behavior-State System; Competing demands task (85); EAS; an unnamed tool (86) demonstrated evidence of relations with other variables—in at least one validity sub-domain (i.e., convergent, discriminant, and criterion validity).

##### Convergent Validity (i.e., Parenting Measures, Severity of Psychological Symptoms)—Observational tools

Seven of the 16 observational tools demonstrated evidence of convergence with related constructs. Five observational tools [CARE-index; GRS; NICHD; PCERA; an unnamed tool (49)] demonstrated evidence of convergence with a measure of paternal and/or maternal parenting; while four tools (CARE-Index; PCERA; NCATS; SVIA) demonstrated evidence of convergence with paternal and/or maternal psychological symptoms—in some, but not all paternal and/or infant behaviors assessed across the tools.

Convergence With the same construct—i.e., With a measure of paternal parenting

The NICHD extracted from Brown and Cox (115) was the only observational tool which demonstrated evidence of convergence with another measure of paternal parenting—paternal sensitivity demonstrated a moderate convergent association with a measure assessing fathers perceptions and attitudes toward parenting, at 12-months (assessed via a modified version of the Parent Development Interview (116).

Convergence With related constructs—i.e., With a measure of maternal parenting

Five of the 16 observational tools [CARE-index; GRS; NICHD; PCERA; an unnamed tool (49)] demonstrated evidence of convergence with a measure of maternal parenting. Mostly moderate convergent associations between measures of father-infant and mother-infant interactions were evident, across the same behavioral sub-scales, including parent interactive behaviors (i.e., sensitivity, control, remoteness, intrusiveness, cognitive stimulation, synchrony, affectionate touch) and affect (i.e., positive and negative affect)—with the majority of evidence based on interaction assessments conducted across the 1st year postnatal and at 24-months.

Convergence With related constructs—i.e., With the severity of paternal and/or maternal psychological symptoms

Four of the 16 observational tools (CARE-index; PCERA; NCATS; SVIA) demonstrated evidence of convergence with paternal symptom level difficulties (i.e., primarily depression and to a lesser extent, anxiety and alcohol dependence)—with paternal psychological assessments conducted antenatally and during the 1st year postnatal (2-12-months). This was primarily demonstrated through weak-to-moderate convergent associations with paternal interactive behaviors (i.e., warmth, sensitivity, overall quality of father-infant interactions) speech (i.e., verbalisations) and affect (i.e., positive and negative affect), as well as infant interactive behaviors (i.e., difficulty, responsiveness and food refusal behaviors).

In addition, three of the four tools (CARE-index; NCATS; PCERA) demonstrated evidence of convergence with maternal symptom level difficulties symptoms (i.e. depression)—with maternal psychological assessments conducted 2-3-months postnatal. This was primarily demonstrated through weak-to-moderate associations with paternal interactive behaviors (i.e., control, unresponsiveness, warmth, sensitivity, and the overall quality of father-infant interactions) and affect (i.e., negative affect).

In general, increased levels of both paternal and maternal symptom level difficulties were associated with less optimal father-infant interactions—with most father-infant interaction assessments conducted during the 1st year postnatal (2- to-12-months).

##### Discriminant Validity (i.e., Known Groups Validity—Groups Based on Parent Clinical Diagnostic Status, and Level of Symptoms)—Observational Tools

Four observational tools (GRS; PCAMS; PCERA; P-PATS) demonstrated evidence of group discrimination between clinical diagnostic groups; while two tools (NCATS; PCERA) demonstrated evidence of discrimination between symptom-levels groups—in some, but not all paternal and/or infant behaviors assessed across the tools.

Discrimination Between Parent Clinical Diagnostic Groups

Four of the 16 observational tools (GRS; PCAMS; PCERA; P-PATS) demonstrated evidence of discriminant validity between paternal clinical diagnostic groups only (i.e., primarily presence v absence of clinical depression, and to a lesser extent, alcohol dependence)—with the majority of clinical diagnostic assessments conducted at 2-3-months postnatal.

This was demonstrated through evidence of clinical group discrimination across paternal interactive behaviors (i.e., sensitivity, responsiveness, gentle touch, active engagement and excitation), speech (i.e., attentional focus of speech), and affect (i.e., negative affect), as well as infant interactive behaviors (i.e., attention).

In general, fathers with diagnosed psychopathology displayed less optimal father-infant interactions, compared to fathers with no diagnosis—with most father-infant interaction assessments conducted during the 1st year postnatal (~2-to-12-months).

Discrimination between parent symptom-level groups

Two of the 16 observational tools (PCERA; NCATS) demonstrated evidence of discriminant validity between maternal symptom level groups only—i.e., high v low maternal depressive symptom groups (based on EPDS cut-off scores). This was demonstrated through evidence of group discrimination across paternal interactive behaviors (i.e., overall quality of father-infant interactions), affect (i.e., fathers enjoyment and pleasure in interaction), as well as infant interactive behaviors (i.e., negative affect), as well as infant affect (i.e., negative affect).

Evidence from these two studies utilizing the two tools was mixed. While some evidence suggests that fathers whose partner reported symptom level difficulties scoring above the cut-off (EPDS > 10; assessed at 2-3 months) displayed less optimal overall father-infant interactions at the same time-point, other research indicates that fathers whose partner reported symptom level difficulties above the cut-off (EPDS > 12; assessed at 2-months) displayed increased levels of positive affect during father-infant interactions at 15–18 months.

##### Criterion Validity (Offspring Outcome Variables)—Observational Tools

Six of the 16 observational tools (Assessment of Mind-Mindedness; Categorical System for Micro-Analysis of the Early Mother–Child Interaction; CARE-Index; GRS; NCATS; NICHD) demonstrated evidence of predictive criterion validity with offspring outcomes—in some, but not all paternal and/or infant behaviors assessed across the tools.

This was demonstrated through mostly moderate predictive associations between paternal interactive behaviors (i.e., sensitivity, responsiveness, control, cognitive stimulation, detachment, disengagement, remote and intrusive behavior, and the overall quality of father-infant interactions), speech (i.e., mind-related comments) and paternal and infant affect (i.e., positive affect) examined in relation to offspring outcomes—i.e., infant attachment security externalizing behavioral problems, language development, executive functioning and mental development.

In general, higher quality father-infant relationships characterized by more sensitive and responsive interactions are associated with more optimal offspring outcomes. The majority of evidence was based on interaction assessments conducted at 3-6-months and at 24-months and offspring outcome measures assessed between 12 and-36-months (follow-up period ranging from 7-to-17-months)—with the exception of two tools (Assessment of Mind-Mindedness; Categorical System for Micro-Analysis of the Early Mother–Child Interaction) examined in relation to child outcomes after an 8-to-11-years follow-up period.

#### Validity of Self-Report Tools Used to Assess the Father-Offspring Relationship

All 18 self-report tools used to assess father-offspring relationship quality or paternal involvement demonstrated evidence of validity in at least one validity sub-domain (i.e., internal structure, content validity, response process and relations with other variables), extracted from the included studies using self-report tools in this review.

##### Internal Structure Validity—Self-Report Tools

The majority of self-report tools (13/18) excluding the PAQ; PBQ; and three unnamed tools demonstrated evidence of internal structure through the measurement of internal consistency, primarily using Cronbach alpha—with authors typically establishing acceptable to good levels of internal consistency, when applied to paternal samples in the included studies in this review.

Furthermore, three self-report tools (CRPR; K-PAFAS, PPAQ) demonstrated evidence of internal structure in the remaining three sub-domains of internal structure (i.e., dimensional structure through within-tool inter-scale correlations, factorial validity, test-retest reliability), while one tool (PBQ) demonstrated evidence in two of these sub-domains (i.e., dimensional structure and test re-test reliability). A further 10 tools demonstrated additional evidence of internal structure in only one of the remaining three sub-domains—i.e., dimensional structure (PAAS), factorial validity [PFAS; PRS; an unnamed tool (28)] and test re-test reliability [CRQ; Parental Involvement Questionnaire; PI; three unnamed tools (10, 17, 88)].

##### Content Validity—Self-Report Tools

Six of the 10 self-report tools developed in paternal or parental samples [K-PAFAS; PAAS; PFAS; PI; PPAQ; an unnamed tool (17)] demonstrated evidence of content validity—through theoretically driven items specific to the measurement of paternal parenting. The K-PAFAS, PAAS and PPAQ additionally demonstrated evidence of expertly reviewed items and item development through interviews with fathers subject to content analysis.

##### Response Process Validity—Self-Report Tools

Three of the 14 self-report tools (K-PAFAS; PAAS; PPAQ) demonstrated evidence of response process validity specific to paternal samples, with modifications made based on fathers' responses during pilot testing.

##### Relations With Other Variables (i.e., Convergent, Discriminant, and Criterion Validity)—Self-Report Tools

Most self-report tools (17/18—excluding the CRQ) extracted across 27 studies demonstrated evidence of relations with other variables—in at least one validity sub-domain (i.e., convergent, discriminant, criterion validity).

##### Convergent Validity (i.e., Parenting Measures, Severity of Psychological Symptoms)—Self-Report Tools

Fourteen of the 18 self-report tools demonstrated evidence of convergence with either the same construct (i.e., paternal parenting) and/or related constructs (i.e., maternal parenting; severity of paternal and/or maternal psychological symptoms).

This included 10 self-report tools [FAB; K-PAFAS; PAAS; Parental Involvement Questionnaire; PBQ; PFAS; PI; PPAQ; two unnamed tools (10, 88)] which demonstrated evidence of convergence with a measure of paternal and/or paternal parenting; while seven tools [CPRP; FAB; HOME-SF; K-PAFAS; PAAS; PAQ; PBQ; PPAQ; an unnamed tool (28)] showed evidence of convergence with paternal and/or maternal psychological symptoms—in some, but not all father-offspring relationship sub-scales assessed across the tools.

##### Convergence With the Same Construct—i.e., With a Measure of Paternal Parenting

Six of the 18 self-report tools [FAB; K-PAFAS; PAAS; PI; two unnamed tools (10, 88)] demonstrated evidence of convergence with another measure of paternal parenting.

This was demonstrated through mostly moderate-large convergent associations between father-offspring relationship sub-scales (i.e., fathers attachment relationship to the fetus/infant and paternal involvement) and another measure of either fathers attachment relationship to the fetus/infant, paternal involvement, or relations with paternal perceived skill/competence—with the majority of evidence based on interaction assessments conducted antenatally and across the 1st year postnatal.

##### Convergence With Related Constructs—i.e., With a Measure of Maternal Parenting

Five of the 18 self-report tools (PAAS; Parental Involvement Questionnaire; PBQ; PFAS; PPAQ) demonstrated evidence of convergence with a measure of maternal parenting.

This included mostly moderate-large convergent associations between measures of the father-offspring and mother-offspring relationship, across the same behavioral sub-scales, including parents attachment relationship to the fetus/infant, parent-infant bonding and involvement—with the majority of evidence based on interaction assessments conducted antenatally and across the 1st year postnatal.

##### Convergence With Related Constructs—i.e., With the Severity of Paternal and Maternal Psychological Symptoms

Nine of the 18 self-report tools [CRPR; FAB; HOME-SF; K-PAFAS; PAAS; PAQ; PBQ; PPAQ; an unnamed tool (28)] demonstrated evidence of convergence with paternal symptom level difficulties (i.e., depression and anxiety)—with the majority of psychological assessments conducted antenatally and across the first postnatal year. This was primarily demonstrated through weak-to-moderate convergent associations with father-offspring relationship sub-scales—i.e., fathers attachment relationship to the fetus/infant, and to a lesser extent, father-infant bonding difficulties and involvement in child-care related activities.

In addition, two of the nine tools (PAAS; PBQ) demonstrated evidence of convergence with maternal symptom level difficulties symptoms (i.e., depression and anxiety)—with maternal psychological assessments conducted antenatally and at 6-weeks, respectively. This was demonstrated through weak associations with father-offspring relationship sub-scales—i.e., fathers attachment relationship to the fetus and father-infant bonding difficulties.

In general, evidence from tools examined in relation to paternal psychopathology suggests that increased symptom level difficulties were associated with less optimal father-infant interactions—with most father-infant interaction assessments conducted antenatally and across the first postnatal year.

In contrast, the two tools which demonstrated evidence of convergence with maternal symptom level difficulties was mixed. While some evidence suggests that fathers whose partner reported increased depressive symptom level difficulties antenatally had more optimal attachment relationships to the fetus, other evidence indicates that fathers whose partner reported increased anxiety and depressive symptom level difficulties (assessed antenatally and at 6-weeks, respectively) had less optimal attachment relationships to the fetus and more bonding difficulties with their infant at 6-months.

##### Discriminant Validity (i.e., Known Groups Validity—Parent Clinical Diagnostic Groups, Symptom-Level Groups)—Self-Report Tools

Two of the 18 self-report tools [PPAQ; an unnamed tool (28)] demonstrated evidence of discrimination between parent symptom-level groups.

##### Discrimination Between Parent Clinical Diagnostic Groups

There was no reported evidence of discrimination between clinical diagnostic groups across the self-report tools included in this review.

##### Discrimination Between Parent Symptom-Level Groups

Two of the 18 self-report tools [PPAQ; an unnamed tool (28)] demonstrated evidence of discriminant validity between paternal symptom level groups only—i.e., high v low paternal depressive symptom groups (based on EPDS cut-off scores). This was demonstrated through evidence of group discrimination across father-infant relationship sub-scales—i.e., fathers attachment relationship to the infant and involvement in child-care related activities.

This evidence suggests that fathers reporting symptom level difficulties scoring above the cut-off (assessed at 1, 4 and 9-months) displayed less optimal attachment relationships with their infant at 1- and 4-months and lower levels of involvement at 9-months.

##### Criterion Validity (Offspring Outcomes)—Self-Report Tools

Four of the 18 self-report tools [CRPR; Parental Involvement Questionnaire; PPAQ; an unnamed tool (17)] demonstrated evidence of predictive criterion validity with offspring outcomes; while three tools [PBQ; PRS; an unnamed measure (89)] demonstrated evidence of concurrent criterion validity only—in some, but not all father-offspring relationship sub-scales assessed across the tools.

This was primarily demonstrated through mostly moderate predictive and concurrent associations between father-infant relationship sub-scales (i.e., fathers attachment relationship to the infant and involvement in child-care related activities) and offspring outcomes (i.e., infant attachment security, difficult temperament, sleep quality and infant social and mental development).

In general, this evidence suggests that higher quality father-infant relationships are associated with more optimal offspring outcomes—with the majority of father-infant interactions assessments conducted across the 1st-year postnatal and offspring outcome measures assessed at 6-to-12-months (follow-up period for predictive associations ranging from 3-to-12-months)—with the exception of one tool (CRPR) assessing outcomes at pre-school age.

#### Validity of Interview Tools Used to Assess Father-Infant Relationship Quality

All four interview tools used to assess father-offspring relationship quality or paternal involvement demonstrated evidence of validity in at least one validity sub-domain (i.e., internal structure, content validity, response process, and relations with other variables), extracted from the included studies using interview tools in this review.

##### Internal Structure Validity—Interview Tools

One of the four interview tools (WMCI) extracted from one study demonstrated evidence of internal structure through the measurement of inter-rater reliability using Cohen's kappa coefficients (ϰ); indicating good levels of agreement between raters when applied to a paternal sample. However, there was no reported evidence of factorial validity or test re-test reliability, across the interview tools included in this review.

##### Content Validity—Interview Tools

One of the four interview tools [an unnamed tool (90)] developed in paternal or parental samples demonstrated evidence of content validity—through theoretically driven items specific to the measurement of paternal parenting. However, there was no reported evidence of content validity, through the expert review of interview domains or item development through interviews with fathers subject to content analysis, across the interview tools included in this review.

##### Response Process Validity—Interview Tools

There was no reported evidence of response process validation across the interview tools developed in parental or paternal samples included in this review.

##### Relations With Other Variables (i.e., Convergent, Discriminant, and Criterion Validity)—Interview Tools

Three of the four interview tools [CDS; Parental Involvement Time Diary (27); WMCI] extracted from three studies demonstrated evidence of relations with other variables—in two validity sub-domains (i.e., convergent and discriminant validity).

##### Convergent Validity (i.e., Parenting Measures, Severity of Psychological Symptoms)—Interview Tools

Three of the 14 self-report tools demonstrated evidence of convergence with either the same construct (i.e., paternal parenting) and/or related constructs (i.e., maternal parenting; severity of paternal and/or maternal psychological symptoms). This included one interview tool (WMCI) which demonstrated evidence of convergence with another measure of paternal parenting; while two tools [CDS; Parental Involvement Time Diary (27)] demonstrated evidence of convergence with paternal and/or maternal psychological symptoms—in some, but not all paternal and/or infant behaviors assessed across the tools.

##### Convergence With the Same Construct—i.e., With a Measure of Paternal Parenting

The WMCI extracted from Hall et al. (83) was the only self-report tool which demonstrated evidence of convergence with another measure of paternal parenting—paternal attachment representations of the infant assessed at 6-months demonstrated a moderate-strong convergent association with a measure of paternal sensitivity, at 24-months (assessed via the NICHD).

##### Convergence With Related Constructs—i.e., With a Measure of Maternal Parenting

There was no reported evidence of convergence with a related measure of maternal parenting across the self-report tools, included in this review.

##### Convergence With Related Constructs—i.e., With the Severity of Paternal and Maternal Psychological Symptoms

One of the four interview tools [Parental Involvement Time Diary (27)] demonstrated evidence of convergence with paternal symptom level difficulties—i.e., paternal dysphoria and depression assessed antenatally and through 3-to-9-months. This was demonstrated through weak and moderate convergent associations with paternal measures of involvement in child-care related activities.

In addition, two of the four interview tools [CDS; Parental Involvement Time Diary (27)] demonstrated evidence of convergence with maternal symptom level difficulties symptoms (i.e., depression and anxiety)—with maternal psychological assessments conducted antenatally and through 3-to-12-months. This was demonstrated through weak associations with father-offspring relationship sub-scales—i.e., fathers attachment relationship to the fetus and father-infant bonding difficulties.

Evidence from the Parental Involvement Time Diary suggests that increased paternal antenatal and postnatal symptom level difficulties were associated with lower levels of paternal involvement at 3-to-9-months. In relation to maternal symptom level difficulties, evidence was somewhat mixed. Findings from both tools suggest that fathers whose partner reported increased anxiety and depression symptom level difficulties had increased levels of involvement in child-care related activities antenatally and across the first 6-months. In contrast, there was some indication from the CDS suggesting that partners increased symptom level difficulties from 7-to-12-months were associated with lower levels of involvement.

##### Discriminant Validity (i.e., Known Groups Validity: Parent Clinical Diagnostic Groups, Symptom-Level Groups)—Interview Tools

The CDS extracted Goodman et al. (10) was the only interview tool which demonstrated evidence of discrimination between clinical diagnostic groups:

Evidence from the CDS demonstrated evidence of discrimination between paternal clinical diagnostic groups (i.e., paternal lifetime history v no history of clinical depression)—fathers lifetime history of depression was associated with their level of accessibility to the child and involvement in child-care related activities at 3, 6, and 12-months (*Note*, mean values for each group were not reported since this relationship was examined to test for potential control variables, and was not a main focus of the study).

##### Criterion Validity (Offspring Outcomes)—Interview Tools

There was no reported evidence of predictive or concurrent criterion validity with offspring outcomes across the interview tools, included in this review.

## Discussion

In this systematic search and review, we examined the application and performance of tools used to assess the father-offspring relationship when examined in the context of paternal and maternal psychopathology and/or offspring outcomes, during the perinatal period and up to 24-months postpartum. The review identified 38 unique tools used to assess the father-offspring relationship, extracted from 61 studies. Of these, 10 tools were utilized in the context of paternal psychopathology, three in the context of maternal psychopathology, and seven in the context of both maternal and paternal psychopathology, while nine tools were utilized in the context of offspring outcomes only. The remaining nine tools were used in the context of both parental psychopathology (i.e., paternal, and/or maternal psychopathology) and offspring outcomes.

Evidence is discussed in relation to three key themes: (1) the tools' psychometric robustness across four validity domains (i.e., content validity, internal structure, response process validity and validity based on relations with other variables); (2) the tools' characteristics; and (3) methodological study features in which the tool was utilized. Considerations and recommendations are drawn from the synthesized evidence to help guide tool selection, as well as areas of future research.

### Psychometric Robustness of Tools Used to Assess the Father-Offspring Relationship

The synthesized validity evidence demonstrates that most tools included in this review were originally developed in maternal samples. Of the tools developed in parental and paternal samples, nine tools demonstrated evidence of content validation specific to paternal samples—i.e., two observational (PCAMS, P-PATS), six self-report tools [K-PAFAS; PAAS; PFAS; PI; PPAQ; an unnamed tool (17)] and one interview tool [an unnamed tool (90)]. Moreover, evidence supporting content validity through the inclusion of subject matter experts or item development supported by interviews with fathers was rarely reported—apart from three self-report tools (K-PAFAS; PAAS; PPAQ).

Nonetheless, the majority of observational and self-report tools, as well as one interview-based tool (WMCI), regardless of the sample they were initially developed in, did demonstrate evidence of internal structure—with authors typically establishing adequate levels of inter-rater reliability or internal consistency, when applied to paternal samples in the included studies. However, there was a general lack of evidence across all tools to support their factor and dimensional structure, test re-test reliability, as well as the processes underlying item response and performance in the context of fathers. Only two self-report tools (K-PAFAS, PPAQ) demonstrated additional evidence of internal structure across these three sub-domains, whereas only evidence across two domains (i.e., dimensional structure and test re-test reliability) was demonstrated in one observational tool (NICHD). There was no such evidence in any of the four interview tools included in this review.

While it is encouraging that some research over the last few years has focused on the development of tools with a theoretical focus on the father-offspring relationship, the synthesized evidence suggest that the majority of father-offspring relationship assessments were based on tools originally intended to assess the mother-offspring relationship. Given evidence from some studies suggesting that mothers and fathers differ in the way they understand and interact with their offspring (37), future research is not only needed to support the development of new instruments which are theoretically guided by the paternal literature, but also specific validation studies to assess the application of existing tools developed in maternal samples in terms of their factor and dimensional structure. Particular attention should also focus on illuminating the processes underlying item response and performance—especially regarding observational tools. This is important since observations of parent and infant behavior are often carried out in settings that may be influenced by several demand characteristics (e.g., videotaping, presence of an observer, and structured settings) (117). In addition, given the limited evidence of test re-test reliability, whether measurements of the father-offspring relationship are consistent over multiple time-points requires further exploration.

With regards to the tools' relations to other variables, tools were generally applied in the context of paternal psychopathology—with evidence generally suggesting that the presence of paternal psychopathology (including both clinical and symptom level difficulties) was associated with less optimal father-offspring relationships. This was based on evidence from 14 tools demonstrating evidence of convergence with paternal symptom level difficulties, as well as some evidence discriminating between paternal symptom-level groups—i.e., four observational (CARE-Index; NCATS; PCERA; SVIA), nine self-report [CRPR; FAB; HOME-SF; K-PAFAS; PAAS; PAQ; PBQ; PPAQ; an unnamed tool (28)] and one interview tool [Parental Involvement Time Diary (27)]. To a lesser extent, some tools also demonstrated an ability to discriminate between paternal clinical diagnostic groups—i.e., four observational (GRS; PCAMS; PCERA; P-PATS) and one interview tool (CDS).

In contrast, far fewer tools used to assess father-offspring relationship were utilized in the context of maternal psychopathology, and overall, the extracted evidence utilizing these tools was mixed—i.e., supporting both the “spill over” [e.g., (10, 27, 118)] and “compensatory/buffering” hypotheses [e.g., (9, 10, 119)]. This was based on evidence from seven tools demonstrating evidence of convergence with maternal symptom level difficulties, as well as some evidence discriminating between maternal symptom-level groups—i.e., three observational (CARE-Index; PCERA; NCATS), two self-report (PAAS; PBQ), and two interview tool [CDS; Parental Involvement Time Diary (27)].

Taken together, how tools perform in the context of parental psychopathology is mostly based on their convergence with the severity of psychological symptoms—primarily depressive symptoms assessed across the first postnatal year. However, their ability to detect group differences in clinical populations is less clear. Hence, the application of these tools in the context of clinical level difficulties, requires further testing, before they can be reliably applied across a range of clinical populations.

Tool performance when utilized in the context of other measures of parenting was identified across a relatively small number of the included tools. This including eight tools which demonstrated evidence of convergence with another measure of paternal parenting—i.e., one observational (NICHD), six self-report [FAB; K-PAFAS; PAAS; PI; two unnamed tools (10, 88)] and one interview tool (WMCI); while 10 tools showed evidence of convergence with a measure of maternal parenting—i.e., five observational [CARE-index; GRS; NICHD; PCERA; an unnamed tool (49)] and five self-report tools (PAAS; Parental Involvement Questionnaire; PBQ; PFAS; PPAQ).

Nonetheless, the father-offspring relationship is not only shaped by, for example, the family environment and exposure to psychopathology, but also the degree of coordination between specific parenting behaviors. For example, there is some evidence to suggest that parents who consider themselves as competent and skilled parents are more likely to be involved in child-care (120, 121). Hence, further research is needed to understand the extent to which tools assessing the father-offspring relationship can detect associations with theoretically related measures of the same construct (i.e., paternal parenting).

Similarly, evidence supporting the predictive value of tools utilized in the context of offspring outcomes was generally limited across the tools included in this review. This included six observational (Assessment of Mind-Mindedness; Categorical System for Micro-Analysis of the Early Mother–Child Interaction; CARE-Index; GRS; NCATS; NICHD) and four self-report tools [CRPR; Parental Involvement Questionnaire; PPAQ; an unnamed tool (17)] which demonstrated evidence of predictive criterion validity with offspring outcomes; suggesting that less optimal father-offspring relationships were associated with less optimal offspring outcomes—primarily examined in relation to attachment security, temperament and behavioral problems. Thus, in choosing a particular tool, researchers should consider the stage of offspring development in which the tool would be applied to. Also, if assessing offspring outcomes, the type of outcome requires consideration—given the application of tools have not been widely examined in the context of varied offspring outcomes.

### Performance of Tools Used to Assess the Father-Offspring Relationship in the Context of the Tool's Characteristics

Several important variations potentially influencing tools' performance were identified. These are: (i) differences in the behaviors assessed across tools, (ii) the conceptualization of father-offspring relationship constructs, and (iii) diverse scoring formats applied to these constructs.

The tools included in this review assessed a broad range of behaviors within the father-offspring relationship. While self-report and interview tools generally evaluated overall measures of the father-offspring relationship (e.g., composite scores of paternal involvement, fathers attachment relationship to their infant), observational tools assessed more discrete domains of behaviors on a broader spectrum (e.g., sensitivity, intrusiveness, paternal speech). However, there was considerable variability across the tools in how behaviors within the father-offspring relationship were associated with either offspring outcomes or parental psychopathology—especially in relation to observational tools.

This is partly due to tools examining a range of constructs applied to diverse samples, and in relation to different correlates. For example, in relation to observational measures, paternal displays of negative affect assessed *via*. the GRS were higher in fathers in the depressed group (defined in this study as fathers with a positive SCID and/or BDI/EPDS depressive symptoms above the cut-off), compared to non-depressed, at 2–16 weeks (23). However, in another study no differences were reported between depressed (defined here as fathers with a positive SCID only) and non-depressed groups in the same construct, using the same measure, at 3-months (122). Hence the selection of a specific tool would be determined by the nature of the sample, the construct intended to be measured, as well as the correlates being examined in relation to these constructs.

Furthermore, how specific constructs of the father-offspring relationship have been described and conceptualized, varies across the tools. This is especially relevant for paternal reported measures of involvement. While some tools have measured involvement in terms of overall accessibility, responsibility and engagement in child-care related activities, others have focused on engagement in physical care or enrichment activities, or overall composite measures of these components. Thus, at present, the tools included in this review which have been applied to examine fathers' involvement report mixed findings when examined in relation to parental psychopathology and offspring outcomes. This is likely a result of the evolving framework surrounding the paternal involvement literature [e.g., (54, 123)]—specifically, a shift away from the focus of total father engagement, toward a more integrative approach focused on the quality of fathers' engagement and its importance for child development (54). Hence, an integrated approach in the selection of tools used to assess paternal involvement is recommended. One that includes both the quantity of paternal engagement, as well as the quality—i.e., positive engagement, warmth-responsiveness, control (54).

Differences in scoring formats may also contribute to the tools' performance. Specifically, observational measures of father-infant relationship quality mostly incorporated either frequency or global ratings of behavior. However, there appears to be little consensus about how these rating methods are applied to specific behavioral domains. For instance, in one study, sensitivity was assessed *via*. the Categorical System for Micro-Analysis of the Early Mother–Child Interaction using frequencies and time-durations for a composite of behaviors (loving, close, and vocalizing) (124), whereas other studies (23, 122, 125) have assessed sensitivity through the GRS, which incorporates global rating scales.

Therefore, decisions surrounding the selection of a tool should be guided by the behavior intended to be assessed, the rationale of the study, and should consider the strengths and weakness of the coding frameworks utilized. For example, global ratings are useful for capturing the quality of interactive behaviors and are more time-efficient in comparison to frequency coding (32, 126). On the other hand, frequency coding is a relatively objective measure yielding information about frequencies and duration, as well as permitting sequential time-series analysis. Thus, this type of coding may be more suited to address questions of quantity—although, this coding process is more time consuming in comparison to a global rating and often requires specific event logging software (126).

Likewise, there is variation regarding scoring formats applied to the construct of paternal involvement. For example, self-report tools included in this review primarily assessed “relative” measures of father involvement (i.e., proportion of father involvement relative to the mother), whereas interview measures most often assessed “absolute” values of paternal involvement (e.g., total amount of time spent engaged in child-care related activities).

While adopting “relative” measures to assess paternal involvement may, in part, reduce social desirability bias (i.e., high rating across all aspects of paternal involvement) and indicate levels of involvement within a family, “absolute” measures also need consideration. Hence, adopting both relative and absolute measures may be beneficial, given their potentially differential links with measures of parental psychopathology and offspring outcomes.

### Application and Performance of Tools Used to Assess the Father-Offspring Relationship in the Context of Study Methodologies

Several methodological aspects potentially influencing tools' performance were identified. These include: (i) study setting and sample characteristics, (ii) study design, (iii) interaction setting, and (iv) timing and nature of correlates (i.e., parental psychopathology and offspring outcomes) examined in relation to assessments of the father-offspring relationship.

Most tools used to assess the father-offspring relationship were applied to homogeneous samples—comprising mainly Caucasian fathers from largely high-income settings, well-educated fathers residing in the family home and from two-parent families of middle to high SES. More diverse samples have been relatively unexplored, despite differences in paternal interactive styles across cultures. For example, physical play as an essential hallmark of paternal interactive style is less often evident in non-western samples [for a review, see (50)]. Similarly, fathers of White, Hispanic, and Black ethnicity may differ in their level of involvement, partly explained by cultural factors (127).

Importantly, the majority of tools included in this review were applied to relatively small to modest sample sizes of <200 participants recruited from samples across single hospital or clinic sites (e.g., maternity, antenatal clinics)—with up to one third of included tools in this review applied to samples of <100 participants. Only two observational (NICHD) and six self-report tools [i.e., CRPR; CRQ; HOME-SF; PBQ; PPAQ; an unnamed tool (28)] were applied to larger samples of more than 500 participants. It is possible that small sample sizes can reduce statistical power consequently leading to increased likelihood of Type II errors, as well inflate effect size estimates and lead to low levels of reproducibility (128). Hence, caution is needed when drawing conclusions about the psychometric robustness of these tools, as evidence is primarily based on modest sample sizes.

Most of the tools used to assess the father-offspring relationship included in this review were applied to cross-sectional data analyses. Those tools which were utilized in the context of prospective analyses had relatively short follow-up periods—on average around 10-months. In addition, assessments of the father-offspring relationship and their correlates (i.e., parental psychopathology and offspring outcomes) were mostly assessed at one time-point—especially across studies employing observational tools. Thus, in choosing a particular tool the stage of offspring development should be given careful consideration—given the infants rapid development over the 1st year post-partum and high susceptibility to the quality of the parent-offspring relationship (36, 129).

It is also likely that the tool's performance may depend on the interaction setting. For example, there is some suggestion that free-play sessions may allow more depressive behaviors to emerge, leading to increased displays of parental withdrawal, whereas other settings, such as infant-seat and structured task-based play, require greater involvement from the parent (122, 130). Yet, few tools included were applied within varied interaction settings (i.e., structured v free play; free-play v infant-seat setting). Hence, the interaction context in which a tool is applied should be given consideration—as evidence demonstrates that the identification of specific interactive behaviors may only be observable when assessed under certain interaction contexts (118, 122, 125). This may be determined by the nature of the questions being asked within a research context.

Moreover, most tools used to assess the father-offspring relationship included in this review were examined in relation to paternal psychopathology during the antenatal period and within the first 3-6-months following birth. Yet, there is evidence to suggest that, in comparison to mothers, father's vulnerability to psychopathology may peak later on toward the second half of the 1st year postpartum (2)—with potentially differential effects on the father-offspring relationship (7). Hence, researchers need to be cautious in the application of tools used to assess the father-offspring relationship when examined in relation to paternal psychopathology beyond the first 6-months.

Similarly, the performance of tools used to assess the father-offspring relationship likely depends on the onset and duration of maternal psychopathology. For example, in one study utilizing the CDS to assess paternal levels of involvement, higher levels of maternal depressive symptoms from 4 to 6-months predicted increased levels of paternal-reported weekend engagement and accessibility in child-care at 12-months. In contrast, depressive symptoms from 7 to 12-months predicted lower levels of paternal reported weekend accessibility at 12-months (10). This suggests that fathers may be able to compensate for the potentially negative influence maternal psychopathology—but only up to a point. Given the limited evidence and inconsistent findings, consideration should be given to the onset of psychopathology when choosing a tool to assess the father-offspring relationship in the context of maternal perinatal psychopathology. Importantly, more research is needed to help in identifying a tool sensitive to paternal caregiving in the context of perinatal maternal psychopathology.

Many tools used to assess the father-offspring relationship included in this review were also examined in relation to parental depression, and to a lesser extent, anxiety, and alcohol dependence. In addition, paternal anxiety and depression symptoms may have differential influences on the father-offspring relationship [e.g., (21)]. However, the performance of tools may be influenced by other types of parental psychopathology which may give rise to different interactional patterns. For instance, there is some evidence to suggest that unresolved trauma (for example, trauma following a traumatic childbirth) in fathers impacts upon their ability to bond with their infant (131).

Taken together evidence from this study points toward the validity of tools in relation to depression (i.e. symptom level difficulties). Hence, the application of these tools in the context of other mental health conditions, requires further testing, before they can be reliably applied to different populations.

Finally, the tools included in this review were examined in relation to a range of offspring outcomes domains. This primarily included behavioral problems, attachment security, difficult temperament—and to a lesser extent, infant sleep quality, psychological difficulties, and indices of social and cognitive development. However, most tools assessed the father-offspring relationship at one point in time, around the 1st-year postpartum—with relatively short follow-up periods for infant outcomes (mostly between 2 and 12-months). Hence, further research focused on different developmental stages may help identify and target specific patterns of paternal behavior which have a predive value in terms of developmental outcomes. This would also support the development of objective predictive tools which might be useful in early intervention programs to help fathers better interact with their children.

### Strengths and Limitations

The search strategy for this review was intentionally broad to capture relevant literature and its performance was tested to ensure optimal sensitivity. Furthermore, decisions regarding inclusion of potentially relevant literature were made by two reviewers, to promote the robustness of decision-making. Nonetheless, it is possible that some studies would have been missed—for example, where there was no indication in the abstract of the use of a tool used to assess the father-offspring relationship in relation to parental psychopathology and/or offspring outcomes, during the perinatal and up to 24-months postpartum. Moreover, this review focused specifically on the literature concerning the direct relationship between the father and the offspring—i.e., father's direct interactions with their offspring and their involvement in child-care related activities.

However, other important aspects of the father-offspring relationship, such as offspring attachment security, fathers attitudes and beliefs which guide parenting and predict the future quality of the father-offspring relationship and level of involvement (132, 133), were not examined.

In addition, since one of the main aims of this review was to examine the performance of tools used to assess the father-offspring relationship in the context of parental psychopathology, other determinants which shape the father-offspring relationship were not examined—including, for example, marital satisfaction, parenting stress, and socioeconomic circumstances (36, 134). Therefore, future research would benefit from exploring how tools used to assess the father-offspring relationship perform when examined in relation to these other determinants.

Finally, since there is currently no “gold standard” measurement to assess father-infant interactive processes, over the perinatal period, our eligibility criteria were focused on observational study designs. While future research could examine the effectiveness and robustness of tools used to assess the father-offspring relationship in the context of intervention studies, it is first necessary to conduct further research to assess the psychometric robustness of these tools when applied to the father-offspring relationship in specific contexts (for example, through validation studies with diverse populations).

### Clinical Implications

Based on the synthesis of evidence in this review, recommendations, and clinical implications are outlined to help guide tool selection. This is discussed in relation to the applicability and performance of tools across clinical populations, as well as the feasibility of implementing such assessment tools for use in clinical practice.

First, given the limited evidence of tools examined in the context of a clinical diagnosis in fathers—with most evidence on symptom level difficulties—caution should be taken in their application within a clinical setting. Similarly, based on the tools included in this review, there is no current evidence to support the application of father-infant interaction tools in families where the mother is clinically unwell.

Hence, at present, it is not possible to recommend one specific tool type over another for use within a clinical setting. Moreover, there is limited evidence to assess the clinical relevance of interaction patterns since typical distributions of clinical data and clinically valid cut-off scores have not been widely established. Thus, before such clinical data is available, a multimethod approach utilizing a range of tool types may be beneficial in providing a more accurate assessment—with the potential to compare interaction patterns across the tool types.

Second, these assessment tools may offer a potentially valuable resource in a variety of clinical settings—for example, for use in preventative screening to identify interactional difficulties, to support the formulation of risk assessments, and for the evaluation of treatment outcomes in parent-infant interventions. There are potentially important clinical applications for the identification of father-infant dyads which may benefit from early intervention programs, such as video-feedback interventions which have already provided encouraging results in non-clinical samples (135).

Of note, the implementation of father-offspring relationship tools within routine clinical assessments may depend on feasibility and accessibility for use by health workers from a range of professional backgrounds. For instance, observational tools are often complex and lengthy for clinical use demanding considerable resources—including lengthy assessments, extensive training and experience, reliability maintenance and testing, as well as high training costs. Hence, services will need to consider the best ways to invest in staff training and the maintenance of these skills over time. This may include, for example, the development of standardized online training modules which include examples of interaction assessments (across a variety of normative and clinical populations), integrated video feedback clips and standardized self-assessment reliability tests. This could ease the burden on training and increase access in clinical practice. Crucially, the consideration of a multi method approach for the assessment of the father-offspring relationship would also be beneficial.

### Conclusion

The father-infant relationship is important for optimal offspring outcomes; hence we have reviewed the application and performance of tools used for their assessment. Evidence concerning the psychometric robustness of father-offspring relationship tools, in the context of parental psychopathology and/or offspring outcomes, was generally limited. Hence, tool selection should be guided by the research aims of the study, the intended purpose of the tool and should also consider the tools' performance in terms of its of psychometric properties, the characteristics of the tools and the study methodology within which the tool will be embedded. Furthermore, given the strengths and limitations of each mode of assessment, future studies may benefit from a multimethod approach to assessing the father-offspring relationship, which may provide a more accurate assessment. Future research is also needed, on a large scale, to replicate existing studies which have utilized tools to assess the father-offspring relationship, as well as meta-analytic studies, to validate existing findings. With particular attention on the application of these tools to diverse populations (that may include a range of both symptom level difficulties and diagnosed mental health conditions), larger sample sizes and longer follow-up periods. This will help to elucidate how behaviors within the context of the father-offspring relationship unfold overtime and relate to different offspring developmental stages, together with the influence of parental psychopathology at different stages during the perinatal period and up to 24-months postpartum.

## Author Contributions

VS, ZD, JD, JI, and FB contributed to the conception and design of the study. All authors were involved with screening records for inclusion based on the title and abstract, with JS and VS responsible for deciding which full-text articles were included in the review. Data extraction was undertaken by JS and VS, who led the synthesis process. The synthesis was refined by team discussions (all authors). JS and VS drafted the paper. All authors critiqued the manuscript for important intellectual content.

## Conflict of Interest

The authors declare that the research was conducted in the absence of any commercial or financial relationships that could be construed as a potential conflict of interest.
