# Supplementary material for: The Applicability and Performance of Tools Used to Assess the Father-Offspring Relationship in Relation to Parental Psychopathology and Offspring Outcomes
Source: Front Psychiatry. 2021 Jan 5;11:596857. doi: 10.3389/fpsyt.2020.596857 (PMC7814871; doi:10.3389/fpsyt.2020.596857)
Supplement: Supplementary file 1 [file Table_1.docx]

| **Supplementary Materials_Table 1**  Summary of descriptive characteristics of studies (*n* = 31) utilising observational tools to assess the father-offspring relationship quality | | | | | | | | | | | | | | | | | | | |
| --- | --- | --- | --- | --- | --- | --- | --- | --- | --- | --- | --- | --- | --- | --- | --- | --- | --- | --- | --- |
| **Study characteristics** | |  | **Paternal sample details** | | | |  | **Father-offspring relationship assessment** | | | | |  | | |  | **Correlates examined in relation to the father-offspring relationship** | | |
| Study reference | Country/extracted data analyses |  | *N* | | Paternal socio-demographic details | |  | Tool used to assess the father-offspring relationship | Location/ interaction setting/ duration | Father-offspring relationship construct / behavioural domains | | | Time-point | | |  | Parental psychopathology / offspring outcomes | | Time-point |
| **Studies utilising observational tools to assess father-infant relationship quality (*n* = 16)** | | | | | | | | | | | | | | | | | | | |
|  |  |  |  | |  | |  |  |  |  | | |  | | |  |  | |  |
| **Arnott & Meins (2007)** | UK  (L) |  | 15 | | - 36 years - Mostly white ethnicity, lower-middle class SES, higher education | |  | Assessment of mind-mindedness (Meins et al., 2001) | -Lab  -Free-play  -With toys  -30-mins | ***Father-infant relationship quality:***  *Paternal speech*: mind-related comments | | | 6-m | | |  | ***Offspring outcomes***  Infant-attachment security | | 15-m |
|  |  |  |  | |  | |  |  |  |  | | |  | | |  |  | |  |
| **Beal (1989)** | USA  (CS) |  | 44 | | - 28 years - Mostly white ethnicity, working class SES, 14-years education | |  | Unnamed tool (Pepi, 1981) | -Home  -Infant-seat  -With toys  -2-mins | ***Father-infant relationship quality:*** *Overall quality of father-infant interactions* | | | 8-wk | | |  | ***Offspring outcomes***  Infant difficult temperament | | 2-m |
|  |  |  |  | |  | |  |  |  |  | | |  | | |  |  | |  |
| **Brown & cox (2019)** | USA  (CS) |  | 122 | | - 29 years - 42% had professional / semi-pro occupations - 97% European American | |  | National Institute of Child Health and Human Development coding scales (NICHD; NICHD Early Child Care Research Network, 2000) | -Home  -Free-play  -With toys  -15-mins | ***Father-infant relationship quality:***  *Paternal sensitivity* | | | 12-m | | |  | ***Offspring outcomes***  Infant-attachment security | | 12-m |
|  |  |  |  | |  | |  |  |  |  | | |  | | |  |  | |  |
| **Brown,**  **Mangelsdorf** USA  **& Neff (2012)** (CS) | |  | 115 | | - Mostly European American - 82% had higher education | |  | Competing Demands Task (Smith & Pederson, 1988) | - Lab - Fathers completed a self-report whilst attending to child’s needs - 10-mins | ***Father-infant relationship quality:***  *Paternal sensitivity* | | | 13-m | | |  | ***Offspring outcomes***  Infant-attachment security | | 13-m |
|  |  |  |  | |  | |  |  |  |  | | |  | | |  |  | |  |
| **Cerniglia et al. (2014)** | Italy  (CS) |  | 77 | | - 35 years - Mostly Caucasian, middle class SES, married | |  | Observation Scale for Mother-Infant Interactions during Feeding (SVIA; Chatoor et al., 1997) | - Home - Feeding interaction - 20-mins | ***Father-infant relationship quality:***   - *Paternal interactive behaviours:* interactional conflict - *Paternal affect:* Affective state - *Infant interactive behaviours:* Food refusal behaviours - *Dyadic affect:* Dyadic affective state | | | 24-m | | |  | ***Paternal psychopathology***  Psychological symptoms | | 24-m |
|  |  |  | |  | |  |  |  | |  |  |  | |  |  | | |  |  |
| **Chabrol et al. (1996)** | France  (CS) |  | 20 | | *n/r* | |  | Behaviour-State System (Cohn et al., 1986) | -Home  -Infant-seat  -With toys  -2-mins | ***Father-infant relationship quality:***   - *Paternal interactive behaviours:* elicit, play state - *Paternal affect:* negative state - *Dyadic interactive behaviours:* elicit/attend state. play state - *Dyadic affect**:* negative, neutral state | | | 3-6m | | |  | ***Maternal psychopathology***  Clinical diagnosis of Major Depressive Disorder based on DSM-III-R | | 3-6 m |
|  |  |  |  | |  | |  |  |  |  | | |  | | |  |  | |  |
| **Edhborg et al. (2003)** | Sweden  (L) |  | 25 | | - All fathers present at birth - No differences in paternal age or SES between maternal depression groups | |  | Parent Child Early Relational Assessment Scale (PCERA; Clark, 1985, 1999) | - Home - Free-play / structured play - With toys - 5-mins | ***Father-infant relationship quality***   - *Paternal interactive behaviours:* visual contact*,* structuring environment, reading the child’s cues*,* mirroring, physical positive contact - *Paternal speech,* quality of verbalisation - *Paternal affect,* expressed positive affect*,* enjoyment and pleasure - *Infant interactive behaviours:* alertness/interest*,* quality of explorative play, attentional abilities, robustness*,* persistence*,* communicative competence*,* readability - *Infant affect:* expressed affect*,* happy/cheerful, apathetic/withdrawn, anxious/intense*,* irritable/angry*,* sober/serious | | | 15-18m | | |  | ***Maternal psychopathology***  Depressive symptoms | | 2-m |
|  |  |  |  | |  | |  |  |  |  | | |  | | |  |  | |  |
| **Eiden, Colder, Edwards & Leonard (2009)** | USA  (L) |  | 227 | | - 55% post high-school education or a college degree - 87% Caucasian - Age: 33-years - Three groups: one and both parents with alchol dependence and controls, | |  | Parent Child Early Relational Assessment Scale (PCERA; Clark, 1985, 1999) | -Home  -Free-play  -With toys  -10-mins | ***Father-infant relationship quality***   - *Paternal interactive behaviours:* sensitivity, warmth - *Paternal affect:* negative affect | | | 24-m | | |  | **Paternal psychopathology**  -Depressive symptoms  -Alcohol use / dependence  **Maternal psychopathology**  -Depressive symptoms  ***Offspring outcomes***  **-**Self-regulation  -Externalising problems | | 12-m  18-m  12-m  18-m  24-m  36-m |
|  |  |  |  | |  | |  |  |  |  | | |  | | |  |  | |  |
| **Eiden Chavez & Leonard (1999)** | USA  (CS) |  | 204 | | - Most of middle-class SES  - 89% Caucasian  - 55% post-high school education or completed a college degree  - 88% married  - Age: 33-years | |  | Parent Child Early Relational Assessment Scale (PCERA; Clark et al., 1980) – *unpublished version* | -Lab  -Free-play  -With toys  -5-mins | ***Father-infant relationship quality***   - *Paternal interactive behaviours:* sensitivity - *Paternal speech:* verbalisation - *Paternal affect:* positive and negative affect - *Infant interactive behaviours:* responsiveness - *Infant affect:* positive and negative affect | | | 12-m | | |  | **Paternal psychopathology**  -Depressive symptoms  -Alcohol use / dependence | | 12-m |
|  |  |  |  | |  | |  |  |  |  | | |  | | |  |  | |  |
| **Feldman & Eidelman (2007)** | Israel  (L) |  | 108 | | - 32 years - Mostly middle-class SES, average 14-years education, married | |  | Unnamed tool (Feldman & Eidelman, 2007) | -Home  -Free-play  -No toys  -3-mins | ***Father-infant relationship quality***   - *Paternal interactive behaviours:* father affectionate touch - *Dyadic interactive behaviours:* father-infant gaze synchrony | | | 3-m | | |  | ***Maternal psychopathology***  Depressive symptoms | | NEO |
|  |  |  |  | |  | |  |  |  |  | | |  | | |  |  | |  |
| **Fuertes et al., (2016)** | Portugal  (L) |  | 82 | | - 16% had completed higher college education - All fathers had full-time occupations - Majority were married | |  | CARE-Index (infant form; Crittenden, 2003) | -Home  -Free-play  -With toys  -5-mins | ***Father-infant relationship quality***   - *Paternal interactive behaviours:* sensitivity, control - *Infant interactive behaviours:* passivity | | | 9-m | | |  | ***Offspring outcomes***  Infant attachment security | | 12-m  18-m |
|  |  |  |  | |  | |  |  |  |  | | |  | | |  |  | |  |
| **Goodman (2008)** | USA  (CS) |  | 128 | | - Mostly white ethnicity, higher education, in full-time work | |  | Nursing Child Assessment Teaching Scale (NCATS; Sumner & Spietz, 1994) | - Home - Structured play - 1-5 mins | ***Father-infant relationship quality***  Overall synchrony in father-infant interactions | | | 2-3m | | |  | **Paternal psychopathology**  Depressive symptoms | | 2-3 m |
|  |  |  |  | |  | |  |  |  |  | | |  | | |  |  | |  |
| **Hall et al. (2014)** | Netherlands  (L) | | 150 | | - 35 years - Mostly higher education, first-time fathers, married | |  | National Institute of Child Health and Human Development coding scales (NICHD; NICHD Early Child Care Research Network, 1999) | -Home  -Free-play  -With toys  -15-mins | ***Father-infant relationship quality***  *Paternal interactive behaviours:* sensitivity*,* intrusiveness, withdrawal | | | 24-m | | |  | ***Offspring outcomes***  Infant language development | | 24-m |
|  |  |  |  | |  | |  |  |  |  | | |  | | |  |  | |  |
| **Koch et al. (2019)** | Brazil  (CS) |  | 61 | | - 32-35 years - Mostly completed secondary education, in employment | |  | Global Rating Scales (GRS; Gunning & Murray, 2002) | - Home - Free-play or infant-seat setting - with or w/out toys - 5-mins | ***Father-infant relationship quality***   - *Paternal interactive behaviours:* sensitivity*,* responsiveness - *Paternal affect:* depressive affect - *Infant interactive behaviours:* attention - *Infant affect:* negative affect - *Dyadic behaviours*: quality of interaction | | | 2-16  wk | | |  | ***Paternal psychopathology***  Clinical diagnosis of major depressive disorder | | 2-16 wk |
|  |  |  |  | |  | |  |  |  |  | | |  | | |  |  | |  |
| **Lucassen et al. (2017)** | Netherlands  (CS/L) | | 94 | | - 35 years - Mostly had higher education | |  | Ainsworth 9-point rating scales for sensitivity and cooperation (AMSS; Ainsworth, et al. 1974) | -Lab  -Free-play  -No toys  -5-mins | ***Father-infant relationship quality***  *Paternal interactive behaviours:* sensitivity | | | 14-m | | |  | ***Paternal psychopathology***  History or current depression or anxiety disorder | | ANT |
|  |  |  |  | |  | |  |  |  |  | | |  | | |  |  | |  |
| **Lundy (2003)** | USA  (L) |  | 24 | | - Mean age, 30 years - Majority of white ethnicity - Mostly lower-middle SES - 40% college educated | |  | Assessment of mind-mindedness (Meins et al., 2001) (modified version) | -Lab  -Seat setting  -With toys  -6-mins | ***Father-infant relationship quality***  *Paternal vernal behaviours:* mind-related comments | | | 6-m | | |  | ***Offspring outcomes***  Infant attachment security | | 13-m |
|  |  |  |  | |  | |  |  |  |  | | |  | | |  |  | |  |
| **Magill-Evans & Harrison (1999)** | Canada  (L) |  | 103 | | - Mean age, 32.3 – 31.5 years (preterm, full-term groups) - Education in years: 14.4 – 15.2 years (PT, FT groups | |  | Nursing Child Assessment Teaching Scale (NCATS; Sumner & Spietz, 1994) | -Home  -Structured   play  -1-5 mins | ***Father-infant relationship quality***  Overall quality of father-infant interactions (paternal and infant total NCATS scores) | | | 3-m  12-m | | |  | ***Offspring outcomes***  -Child mental development  -Receptive language  -Expressive language | | 18-m |
|  |  |  |  | |  | |  |  |  |  | | |  | | |  |  | |  |
| **Miller et al., (2019)** | USA  (L) |  | 102 | | - 20% fathers post-graduate education - 84% of White ethnic background | |  | Assessment of mind-mindedness (Meins et al., 2015) | - Home - Snack period and free-play session - With toys - 6/7-mins | ***Father-infant relationship quality***  *Paternal vernal behaviours:* mind-related comments | | | 7-m | | |  | ***Offspring outcomes***  Infant attachment security  Child attachment security | | 15-m  10-y |
|  |  |  |  | |  | |  |  |  |  | | |  | | |  |  | |  |
| **Mills-Koonce et al., (2015)** | USA 1292  (L) | | | | - 1292 parents and their infant - Biological parents - Sample weighted towards low-income families and African American families | |  | National Institute of Child Health and Human Development coding scales (NICHD; NICHD Early Child Care Research Network, 1999) | - Home - Free-play, structured play   -With toys  -10-mins | ***Father-infant relationship quality***   - *Paternal interactive behaviours:* cognitive stimulation, detachment, animation - *Paternal affect:* positive regard   *Note:* individual subscales were composited to form an overall paternal parenting quality | | | 6-m  24-m | | |  | ***Offspring outcomes***  Child cognitive development | | 6-m  15-m  36-m |
|  |  |  |  | |  | |  |  |  |  | | |  | | |  |  | |  |
| **Parfitt et al. (2013)** | UK  (L) |  | 40 | | - 35 years - Mostly of white ethnicity, had higher education and were married | |  | CARE-Index (infant form; Crittenden, 2004) | -Home  -Free-play  -With toys  -3-5 mins | ***Father-infant relationship quality***   - *Paternal interactive behaviours:* sensitivity, controlling*,* unresponsiveness - *Infant interactive behaviours*, difficultly, passivity | | | 3-m | | |  | ***Paternal psychopathology***   - Depressive symptoms - Anxiety symptoms - PTSD symptoms   ***Maternal psychopathology***   - Depressive symptoms - Anxiety symptoms - PTSD symptoms | | ANT 3m  ANT 3m |
|  |  |  |  | |  | |  |  |  |  | | |  | | |  |  | |  |
| **Ramchandani et al. (2013)** | UK  (L) |  | 168 | | - 35 years - Mostly medium to high SES, completed secondary education | |  | Global Rating Scales (GRS; Murray et al. 1996) | -Home  -Floor-mat /   infant-seat  -With toys  -3-mins | **F*ather-infant relationship quality***   - *Paternal interactive behaviours:* sensitivity*,* intrusiveness*,* remoteness - *Paternal affect:* depressive affect | | | 3-m | | |  | ***Offspring outcomes***  Child behaviour problems | | 12-m |
|  |  |  |  | |  | |  |  |  |  | | |  | | |  |  | |  |
| **Rossen et al. (2018)** | AUS  (L) |  | 191 | | - 35 years - Mostly higher education, high SES and were in full-time work | |  | Emotional Availability Scales (EAS; Biringen et al. 2008) | -Home  -Free-play  -With or   w/out toys  -20-mins | ***Father-infant relationship quality***  Overall emotional availability in partner-child interactions | | | 12-m | | |  | ***Paternal psychopathology***   - Depressive symptoms - Stress and anxiety symptoms | | 2-m |
|  |  |  |  | |  | |  |  |  |  | | |  | | |  |  | |  |
| **Sethna et al. (2012)** | UK  (CS) |  | 38 | | - 34-36 years - Mostly white ethnicity, completed diploma or above, married | |  | Paternal Cognitive Attributional Mentalizing Scale (PCAMS; Sethna et al., 2012) | -Home  -Infant-seat  -No toys  -3-mins | ***Father-infant relationship quality***  *Quality of paternal speech:* attentional focus of speech, affective focus of speech, mentalizing comments | | | 3-m | | |  | ***Paternal psychopathology***  Clinical diagnosis of major depressive disorder | | 3-m |
|  |  |  |  | |  | |  |  |  |  | | |  | | |  |  | |  |
| **Sethna et al. (2015)** | UK  (CS) |  | 192 | | - 35 years - Mostly white ethnicity, completed diploma or above, married | |  | Global Rating Scales (GRS; Murray et al. 1996) | -Home  -Floor-mat /   infant-car   seat  -No toys  -3-mins | ***Father-infant relationship quality***   - *Paternal interactive behaviours:* sensitivity*,* intrusiveness*,* remoteness - *Paternal affect:* depressive affect | | | 3-m | | |  | ***Paternal psychopathology***   - Clinical diagnosis of major depressive disorder - Depressive symptoms   ***Maternal psychopathology***  Depressive symptoms | | 3-m  7-wk  3-m |
|  |  |  |  | |  | |  |  |  |  | | |  | | |  |  | |  |
| **Sethna et al. (2018)** | UK  (CS) |  | 38 | | - 34 - 36 years - Mostly white ethnicity, completed higher education, married | |  | Paternal-Physicality, Affect & Touch Scale (P-PATS; Sethna et al. 2018) | -Home  -Free-play  -No toys  -3-mins | ***Father-infant relationship quality***  *Paternal interactive behaviours:* physicality in play*,* playful excitation*,* active engagement, tactile stimulation | | | 3-m | | |  | ***Paternal psychopathology***  Clinical diagnosis of major depressive disorder | | 3-m |
|  |  |  |  | |  | |  |  |  |  | | |  | | |  |  | |  |
| **Sethna et al., (2019)** | UK  (CS) |  | 28 | | - Most held managerial or professional occupations - Majority of white ethnicity - Mean age, 37-years | |  | Global Rating Scales (GRS; Murray et al. 1996) | -Home  -Free-play  -With toys  -5-mins | ***Father-infant relationship quality***  *Paternal interactive behaviours:* sensitivity | | | 3-6 m | | |  | ***Offspring outcomes***  Infant brain development (MRI) | | 3-6 m |
|  |  |  |  | |  | |  |  |  |  | | |  | | |  |  | |  |
| **Tambelli et al. (2015)** | Italy  (CS) |  | 136 | | - 37 years | |  | Observation Scale for Mother-Infant Interactions during Feeding (SVIA; Chatoor et al., 1997) | -Home  -Feeding   interaction  -20-mins | ***Father-infant relationship quality***   - *Paternal interactive behaviours:* interactional conflict - *Paternal affect:* overall affective state - *Infant interactive behaviours:* food refusal behaviours - *Dyadic affect:* dyadic Affective state | | | 3-m | | |  | ***Paternal psychopathology***  Psychological symptoms | | 3-m |
|  |  |  |  | |  | |  |  |  |  | | |  | | |  |  | |  |
| **Tamis-LeMonda et al., (2004)** | USA  (CS/L) |  | 111 | | - 60% European American, 22% African American, 15% Latino and 3% of other ethnic background - 36% college or graduate school - Age range, 19-51 years | |  | National Institute of Child Health and Human Development coding scales (NICHD; NICHD Early Child Care Research Network, 1999) – adapted version | -Home  -Semi-  structured   interaction  -20-mins | ***Father-infant relationship quality***   - *Paternal interactive behaviours:* sensitivity, cognitive stimulation, intrusiveness, detachment - *Paternal affect:* positive and negative regard | | | 24-m | | |  | ***Offspring outcomes***   - Infant mental development - Receptive vocabulary | | 24-m  36-m  36-m |
|  |  |  |  | |  | |  |  |  |  | | |  | | |  |  | |  |
| **Towe-Goodman et al., (2014)** | USA  (L) |  | 620 | | - Fathers mean education in years: 13.15 | |  | National Institute of Child Health and Human Development coding scales (NICHD; NICHD Early Child Care Research Network, 1999) | -Home  -Semi-  structured   interaction  -10-mins | ***Father-infant relationship quality***   - *Paternal interactive behaviours:* sensitivity, cognitive stimulation, intrusiveness, detachment, animation - *Paternal affect:* positive regard   *Note:* individual subscales were composited to form an overall paternal parenting quality | | | 7-m  24-m | | |  | ***Offspring outcomes***  Child executive functioning | | 3-y |
|  |  |  |  | |  | |  |  |  |  | | |  | | |  |  | |  |
| **Trautmann-Villalba et al., (2006)** | Germany  (L) |  | 71 | | - Age range:   30 - 31 years | |  | Categorical System for Micro-Analysis of the Early Mother–Child Interaction (Jorg et al. 1994) | - Lab - Structured play - 5-mins | ***Father-infant relationship quality***   - *Paternal interactive* behaviours: sensitive fathering*.* non-responsiveness - *Paternal affect:* positive emotionality - *Infant affect:* positive, negative emotionality | | | 3-m | | |  | ***Offspring outcomes***  Child behaviour problems | | 8-11y |
|  |  |  |  | |  | |  |  |  |  | | |  | | |  |  | |  |
| **Volling et al., (2019)** | USA  (CS) |  | 195 | | - Mean age, 32-years | |  | National Institute of Child Health and Human Development coding scales (NICHD; NICHD Early Child Care Research Network, 1999) | - Lab - Structured play - 15-mins | ***Father-infant relationship quality***   - *Paternal interactive behaviours:* sensitivity, cognitive stimulation, intrusiveness, detachment - *Paternal affect:* positive and negative regard | | | 12-m | | |  | ***Offspring outcomes***  Infant attachment security | | 12-m |
|  |  |  |  | |  | |  |  |  |  | | |  | | |  |  | |  |
| L = longitudinal; CS = cross-sectional; m = months; wk = weeks; ANT = antenatally; NEO = neonatally | | | | | | | | | | | | | | | | | | | |
