# Supplementary material for: The Applicability and Performance of Tools Used to Assess the Father-Offspring Relationship in Relation to Parental Psychopathology and Offspring Outcomes
Source: Front Psychiatry. 2021 Jan 5;11:596857. doi: 10.3389/fpsyt.2020.596857 (PMC7814871; doi:10.3389/fpsyt.2020.596857)
Supplement: Supplementary file 2 [file Table_2.docx]

| **Supplementary Materials_Table 2**  Summary of descriptive characteristics of studies (*n* = 30) utilising self-report tools to assess the father-offspring relationship – including relationship quality and father involvement | | | | | | | | | | | | |
| --- | --- | --- | --- | --- | --- | --- | --- | --- | --- | --- | --- | --- |
| **Study characteristics** | |  | **Paternal sample details** | |  | **Father-offspring relationship assessment** | | | |  | **Correlates examined in relation to the father-offspring relationship** | |
| Study reference | Country/data analyses extracted |  | *N* | Paternal socio-demographic details |  | Tool used to assess the father-offspring relationship | Self-report items/ time to complete | Father-offspring relationship construct / behavioural domains | Time-point |  | Parental psychopathology / offspring outcomes | Time-point |
| **Studies utilising self-report tools to assess paternal involvement (*n* = 9) and father-offspring relationship quality (*n* = 9)** | | | | | | | | | | | | |
|  |  |  |  |  |  |  |  |  |  |  |  |  |
| **Barry et al. (2011)** | USA  (CS) |  | 152 | - 29 years - Mostly white ethnicity, completed college education, married |  | Unnamed tool (Barnett & Baruch, 1987) | - Items: 15 - Time to complete: *n/r* | ***Father involvement***  Overall involvement in childcare activities | 1-m  12-m |  | ***Paternal psychopathology***  Depressive symptoms | 1-wk  12-m |
|  |  |  |  |  |  |  |  |  |  |  |  |  |
| **Beesley et al., (2019)** | USA  (CS) |  | 166 | - 31 years - Mostly of white ethnicity, higher education, first-time fathers and married |  | Paternal Antenatal Attachment Scale (PAAS; Condon et al., 1993) | - Items: 16 - Time to complete: *n/r* | ***Father-offspring relationship quality***  Fathers overall attachment relationship to the foetus | ANT |  | ***Paternal psychopathology***  -Depressive symptoms  -Anxiety symptoms | ANT |
|  |  |  |  |  |  |  |  |  |  |  |  |  |
| **Brandão et al. (2019)** | Portugal  (CS) |  | 320 | - 33 years - 37% minimum 12-years education |  | Paternal Antenatal Attachment Scale (PAAS; Condon et al., 1993) | - Items: 16 - Time to complete: *n/r* | ***Father-offspring relationship quality***  Fathers overall attachment relationship to the foetus | ANT |  | ***Paternal psychopathology***  -Depressive symptoms  -Anxiety symptoms  ***Maternal psychopathology***  -Depressive symptoms  -Anxiety symptoms | ANT |
|  |  |  |  |  |  |  |  |  |  |  |  |  |
| **Bronte-Tinkew et al., (2007)** | USA  (CS) | 2137 | | - Majority of fathers aged between 17-29 years - 61.3% completed high school education or above - 67.7% of Non-Hispanic Black ethnicity |  | Home Observation for Measurement of the  Environment  (HOME) -Short Form version (Baker & Mott, 1992) | - Items: 8 - Time to complete: *n/r* | ***Father involvement***  Fathers engagement in a range of child care related activities | 12-m |  | ***Paternal psychopathology***  -Depressive symptoms  -Substance use    ***Maternal psychopathology***  -Depressive symptoms | 12-m |
|  |  |  |  |  |  |  |  |  |  |  |  |  |
| **Brown, Mangelsdorf & Neff (2012)** | USA  (CS) |  | 115 | - Mostly European American - 82% had higher education |  | Responsibility Scale (PRS; McBride & Mills, 1993) (adapted version) | - Items: 14 - Time to complete: *n/r* | **Father involvement**  Fathers responsibility in child care activities | 13-m |  | **Offspring outcomes**  Infant-attachment security | 13-m |
|  |  |  |  |  |  |  |  |  |  |  |  |  |
| **Buist et al. (2003)** | Australia  (CS) |  | 225 | - 31 years - Mostly skilled/semi-professional occupations, married |  | Paternal Postnatal Attachment Questionnaire (PPAQ) (unpublished version; Condon & Corkindale, 1998) | - Items: 19 - Time to complete: *n/r* | ***Father-offspring relationship quality***  Fathers overall attachment relationship to the infant | 1-m  4-m |  | ***Paternal psychopathology***  Depressive symptoms | 1-m  4-m |
|  |  |  |  |  |  |  |  |  |  |  |  |  |
| **Condon et al. (2008)** | Australia  (CS) |  | 241 | - Mostly semi / professional occupations, married, employed |  | Paternal postnatal attachment Questionnaire (PPAQ; Condon et al., 2008) | - Items: 19 - Time to complete: *n/r* | ***Father-offspring relationship quality***  Fathers overall attachment relationship to the infant | 6-m  12-m |  | ***Paternal psychopathology***  Depressive symptoms | 6-m  12-m |
|  |  |  |  |  |  |  |  |  |  |  |  |  |
| **Dayton et al., (2019)** | USA  (CS) |  | 51 | - 60% of African American ethnicity  - Majority aged between 18-25-years  - 60% full-time employment |  | Paternal Antenatal Attachment Scale (PAAS; Condon et al., 1993) | - Items: 16 - Time to complete: *n/r* | ***Father-offspring relationship quality***  Fathers overall attachment relationship to the foetus | ANT |  | ***Paternal psychopathology***  -Depressive symptoms  -Anxiety symptoms  -PTSD symptoms  *Note*, an overall composite score (psychiatric distress) of depression, anxiety and PTSD symptoms was examined in relation to fathers attachment | ANT |
|  |  |  |  |  |  |  |  |  |  |  |  |  |
| **de Cock et al., (2017)** | Netherlands  (L) | | 261 | - 85% Dutch  - 68% 9+ years   education  - Mean age, 34  years |  | Paternal Antenatal Attachment Scale (PAAS; Condon et al., 1993)  Paternal postnatal attachment Questionnaire (PPAQ; Condon et al., 2008) | - Items: 16 - Time to complete: *n/r* - Items: 19 - Time to complete: *n/r* | ***Father-offspring relationship quality***   - Fathers overall attachment relationship to the foetus (PAAS) - Fathers overall attachment relationship to the infant (PPAQ) | ANT  6-m 24-m |  | ***Offspring outcomes***  Child executive functioning problems | 24-m |
|  |  |  |  |  |  |  |  |  |  |  |  |  |
| **Edhborg et al. (2005)** | Sweden  (CS) |  | 106 | - 34 years - Mostly higher education, married, 52% first-time fathers |  | Postpartum Bonding Questionnaire (PBQ; Brockington et al., 2001) | - Items: 25 - Time to complete: *n/r* | ***Father-offspring relationship quality***  Father-infant bonding difficulties | 1-wk 8-wk |  | ***Paternal psychopathology***  Depressive symptoms | 2-m |
|  |  |  |  |  |  |  |  |  |  |  |  |  |
| **Feldstein et al. (2004)** | USA  (CS) |  | 38 | - 35 years - Mostly middle-class SES, married |  | Paternal Postnatal Attachment Questionnaire (PPAQ) (unpublished version; Condon & Corkindale, 1998) | - Items: 19 - Time to complete: *n/r* | ***Father-offspring relationship quality***  Fathers overall attachment relationship to the infant | 12-m |  | ***Offspring outcomes***  Infant attachment security | 12-m |
|  |  |  |  |  |  |  |  |  |  |  |  |  |
| **Ferketich & Mercer (1995)** | USA  (CS) |  | 172 | - 30 – 34 years - Most of white ethnicity and married |  | How I feel about my baby now (FAB; Leifers, 1977) | - Items: 10 - Time to complete: *n/r* | ***Father-offspring relationship quality***  Fathers overall attachment relationship to the infant | 1-m  4-m  8-m |  | ***Paternal psychopathology***  -Depressive symptoms  -Anxiety symptoms | 1-m  4-m  8-m |
|  |  |  |  |  |  |  |  |  |  |  |  |  |
| **Fijałkowska & Bielawska-Batorowicz (2018)** | Poland  (CS) | 35 | | - 35 couples  - 37% university  educated  - 29 years |  | Paternal Antenatal Attachment Scale (PAAS; Condon et al., 1993)  Paternal postnatal attachment Questionnaire (PPAQ; Condon et al., 2008) | - Items: 16 - Time to complete: *n/r* - Items: 19 - Time to complete: *n/r* | ***Father-offspring relationship quality***  Fathers overall attachment relationship to the foetus (PAAS)  Fathers overall attachment relationship to the infant (PPAQ) | ANT  2-8 wk |  | ***Paternal psychopathology***  Depressive symptoms | ANT  2-8 wk |
|  |  |  | |  |  |  |  |  |  |  |  |  |
| **Fuertes et al., (2016)** | Portugal  (L) | 82 | | - 16% had completed higher college education - All fathers had full-time occupations - Majority were married |  | Responsibility Scale (PRS; McBride & Mills, 1993) (Portuguese version; Lima, 2005) | - Items: 14 - Time to complete: *n/r* | **Father involvement**  Fathers responsibility in childcare (specifically, involvement in play, primary care and health care) | 12-m  18-m |  | **Offspring outcomes**  Infant-attachment security | 12-m  18-m |
|  |  |  | |  |  |  |  |  |  |  |  |  |
| **Giallo et al. (2015)** | Australia  (CS) | 2662 | | - 34 years - Most born in Australia, medium-high SES, full-time work |  | Child Rearing Questionnaire (CRQ; Paterson & Sanson (1999) | - Items: 21 - Time to complete: *n/r* | ***Father-offspring relationship quality***  Paternal warmth | 3-12  m |  | ***Paternal psychopathology***  Symptoms of psychological distress | 3-12 m |
|  |  |  |  |  |  |  |  |  |  |  |  |  |
| **Goodman et al. (2014)** | USA  (CS/L) |  | 92 | - 36 years - Mostly European American, average of 16 years education, married |  | Unnamed (Goodman et al. 2014) | - Items: 24 - Time to complete: *n/r* | **Father involvement**  Fathers responsibility in childcare | 3-m  6-m 12-m |  | ***Maternal psychopathology***   - Depressive symptoms - Lifetime history of depression or anxiety disorder   ***Paternal psychopathology***   - Lifetime history of depression or anxiety disorder | 3-m  6-m 12-m |
|  |  |  |  |  |  |  |  |  |  |  |  |  |
| **Kerstis et al., (2015)** | Sweden  (CS/L) |  | 727 | *n/r* |  | Postpartum Bonding Questionnaire (PBQ; Brockington et al., 2001) | - Items: 25 - Time to complete: *n/r* | ***Father-offspring relationship quality***  Father-infant bonding difficulties | 6-m |  | ***Paternal psychopathology***  Depressive symptoms  ***Maternal psychopathology***  Depressive symptoms | 6-wk  6-m |
|  |  |  |  |  |  |  |  |  |  |  |  |  |
| **Ip et al., (2018)** | China  (CS/L) |  | 772 | - Most 26 - ≥35y - 65% first time fathers - 55% higher education |  | Paternal postnatal attachment Questionnaire (PPAQ; Condon et al., 2008) | - Items: 19 - Time to complete: *n/r* | ***Father-offspring relationship quality***  Fathers attachment relationship to the infant | 6-wk |  | ***Paternal psychopathology***  Depressive symptoms  ***Offspring outcomes***  Infant social and overall development | 6-wk  6-m |
|  |  |  |  |  |  |  |  |  |  |  |  |  |
| **Mezulis, Hyde & Clark (2004)** | US  (CS/L) |  | 550 | - All couples cohabiting  - All parents in   employment  - English   speaking |  | Childrearing  Practices Report (CRPR; Block, 1965) | - Items: 28 - Time to complete: *n/r* | ***Father-offspring relationship quality***  Fathers report on their parenting style towards their infant | 12-m |  | ***Paternal psychopathology***  Depressive symptoms  ***Maternal psychopathology***  -Depressive symptoms  -Clinical diagnoses  ***Offspring outcomes***  Infant social and overall development | Across infancy |
|  |  |  |  |  |  |  |  |  |  |  |  |  |
| **Noh & Yeom (2017)** | South Korea  (CS) |  | 230 | - 30-39 years - Mostly college educated |  | Korean Paternal-Fetal Attachment Scale (K-PAFAS; Noh & Yeom, 2017) | - Items: 20 - Time to complete: *n/r* | ***Father-offspring relationship quality***  Fathers overall attachment relationship to the foetus | ANT |  | ***Paternal psychopathology***  Depressive symptoms | ANT |
|  |  |  |  |  |  |  |  |  |  |  |  |  |
| **Nugent (1991)** | Ireland  (L) |  | 48 | - 26 years - All working-class SES |  | Unnamed tool (Nugent, 1991) | - Items: n/r - Time to complete: *n/r* | ***Father involvement***  Overall involvement in childcare activities | 1-12 m |  | ***Offspring outcomes***  Cognitive and motor development | 12-m |
|  |  |  |  |  |  |  |  |  |  |  |  |  |
| **Paulson et al. (2006)** | USA  (CS) | 5089 | | - 20-35 years - 57% of white ethnicity, 8% of black, 15% Hispanic, 14% Asian, 6% others, mostly college educate, in full-time work |  | Unnamed tool (Paulson et al., 2006) | - Items: 7 - Time to complete: *n/r* | ***Father involvement***   - *Involvement in enrichment activities*: reading to child, telling stories, singing to child - *Involvement in play activities*: playing peek-a-boo, tickling child, taking child on errands, work/play outside with child | 9-m |  | ***Paternal psychopathology***  Depressive symptoms  ***Maternal psychopathology***  Depressive symptoms | 9-m |
|  |  |  |  |  |  |  |  |  |  |  |  |  |
| **Pisoni et al. (2015)** | Italy  (CS) |  | 80 | - 35 - 37 years - Mostly married |  | Paternal Antenatal Attachment Scale (PAAS; Condon et al., 1993) | - Items: 16 - Time to complete: *n/r* | ***Father-offspring relationship quality***  Fathers overall attachment relationship to the foetus | ANT |  | ***Maternal psychopathology***  -Depressive symptoms  -Anxiety symptoms | ANT |
|  |  |  |  |  |  |  |  |  |  |  |  |  |
| **Ragni et al. (2019** | Italy  (CS) |  | 66 | -36 years  -Two parent   families |  | Unnamed measure - ad hoc item relating to father involvement on the BISQ | - Items: 1 - Time to complete: *n/r* | ***Father involvement***  Fathers level of involvement during bedtime childcare | 8-12m |  | ***Paternal psychopathology***  Overall affective disorders  ***Maternal psychopathology***  Overall affective disorders | 8-12 m |
|  |  |  |  |  |  |  |  |  |  |  |  |  |
| **Seimyr et al. (2009)** | Sweden  (CS) |  | 274 | *n/r* |  | Paternal Fetal Attachment Scale (PFAS; Weaver & Cranley, 1983) | - Items: 23 - Time to complete: *n/r* | ***Father-offspring relationship quality***  Fathers overall attachment relationship to the foetus | ANT |  | ***Maternal psychopathology***  Depressive symptoms | ANT |
|  |  |  |  |  |  |  |  |  |  |  |  |  |
| **Shorey et al. (2018)** | Singapore  (L) | | 201 | - 34 years - 47% Chinese, 34% Malay, 13% Indian, 5% of other ethnicity, most high school education or above, in full-time work |  | Paternal Involvement Scale (PI; Rustia & Abbott, 1993) | - Items: 8 - Time to complete: *n/r* | ***Father involvement***  Overall involvement in childcare activities | 6-m |  | ***Paternal psychopathology***  Depressive symptoms | Birth |
|  |  |  |  |  |  |  |  |  |  |  |  |  |
| **Tikotzky et al. (2010)** | Israel  (L) |  | 56 | - 31 years - Most completed 16 years of education, middle-upper class SES |  | Parental Involvement Questionnaire (Tikotzky et al., 2010) | - Items: 10 - Time to complete: *n/r* | ***Father involvement***  Overall involvement in childcare activities | 1-m  6-m |  | ***Offspring outcomes***  Sleep quality | 1-m  6-m |
|  |  |  |  |  |  |  |  |  |  |  |  |  |
| **Tikotzky et al. (2015)** | Israel  (L) |  | 57 | - 31 years - Most completed 16 years of education, middle-upper class SES |  | Parental Involvement Questionnaire (Tikotzky et al., 2010) | - Items: 10 - Time to complete: *n/r* | ***Father involvement***  Overall involvement in childcare activities | 3-m  6-m |  | ***Offspring outcomes***  Sleep quality | 3-m  6-m |
|  |  |  |  |  |  |  |  |  |  |  |  |  |
| **Wynter et al. (2016)** | Australia  (CS/L) |  | 270 | - 33 years - Mostly university educated, married |  | Parental Attachment Questionnaire (PAQ; Condon & Corkindale, 1998) | - Items: 19 - Time to complete: *n/r* | ***Father-offspring relationship quality***  Fathers overall attachment relationship to the infant | 6-m |  | ***Paternal psychopathology***  Depressive symptoms | 1-m  6-m |
|  |  |  |  |  |  |  |  |  |  |  |  |  |
| **Vreeswijk et al. (2013)** | Netherlands  (CS) | | 301 | - 34 years - Mostly college educated, in employment |  | Paternal Antenatal Attachment Scale (PAAS; Condon, 1993) | - Items: 16 - Time to complete: *n/r* | ***Father-offspring relationship quality***  Fathers overall attachment relationship to the foetus | ANT |  | ***Paternal psychopathology***  -Depressive symptoms  -Anxiety symptoms | ANT |
|  |  |  |  |  |  |  |  |  |  |  |  |  |
| L = longitudinal; CS = cross-sectional; m = months; wk = weeks; ANT = antenatally | | | | | | | | | | | | |
