# Supplementary material for: The Applicability and Performance of Tools Used to Assess the Father-Offspring Relationship in Relation to Parental Psychopathology and Offspring Outcomes
Source: Front Psychiatry. 2021 Jan 5;11:596857. doi: 10.3389/fpsyt.2020.596857 (PMC7814871; doi:10.3389/fpsyt.2020.596857)
Supplement: Supplementary file 3 [file Table_3.docx]

| **Supplementary Materials_Table 3**  Summary of descriptive characteristics of studies (*n* = 4) utilising interview tools to assess the father-offspring relationship – including relationship quality and father involvement | | | | | | | | | | | | |
| --- | --- | --- | --- | --- | --- | --- | --- | --- | --- | --- | --- | --- |
| **Study characteristics** | |  | **Paternal sample details** | |  | **Father-offspring relationship assessment** | | |  |  | **Correlates examined in relation to the father-offspring relationship** | |
| Study reference | Country/extracted data analyses |  | *N* | Paternal socio-demographic details |  | Tool used to assess the father-offspring relationship | Location/ interaction setting/ duration | Father-offspring relationship construct / behavioural domains | Time-point |  | Parental psychopathology / offspring outcomes | Time-point |
| **Studies utilising interview tools to assess paternal involvement (*n* = 3) and father-infant relationship quality (*n* = 1)** | | | | | | | | | | | | |
|  |  |  |  |  |  |  |  |  |  |  |  |  |
| **Frodi et al. (1983)** | Sweden  (L) |  | 51 | - 30 years - Mostly well educated, middle-class SES |  | Unnamed tool (Frodi et al., 1983) | - Home - Interview duration: *n/r* | ***Father involvement***  Overall father involvement in childcare activities | 5-m |  | ***Offspring outcomes***  Infant-attachment security | 11-m  13-m |
|  |  |  |  |  |  |  |  |  |  |  |  |  |
| **Goodman et al. (2014)** | USA  (L) |  | 92 | - 36 years - Mostly European American, completed 16 years of education |  | Child Development Supplement to Panel Study of Income Dynamics Time Diary (CDS; Hofferth et al. 1997) | - Telephone - Interview duration: *n/r* | ***Father involvement***  *Father involvement in positive activities and routine childcare tasks*: average hours of engagement, time spent in one-to-one activity with child, time father is available | 3-m  6-m  12-m |  | ***Maternal psychopathology***   - Depressive symptoms - Lifetime history of depression or anxiety disorder   ***Paternal psychopathology***   - Lifetime history of depression or anxiety disorder | 3-m  6-m  12-m |
|  |  |  |  |  |  |  |  |  |  |  |  |  |
| **Hall et al. (2014)** | Netherlands (L) | | 220 | - 35 years - Mostly completed higher education, first-time fathers |  | Working Model of Child Interview (WMCI; Zeanah et al., 1986) | - Home - Interview duration: *45-mins* | ***Father-offspring relationship quality***  *Paternal attachment representations of the infant:* balance, disengaged, distorted | 6-m |  | ***Offspring outcomes***  Infant language development | 24-m |
|  |  |  |  |  |  |  |  |  |  |  |  |  |
| **Jia et al. (2016)** | USA  (L) |  | 177 | - 30 years - Mostly of white ethnicity, completed higher education, first-time fathers |  | Parental Involvement Time Diary (Jia et al., 2016) | - Home/   phone   - Interview duration: *n/r* | ***Father involvement***   - *Father involvement in enrichment activities*: (e.g., reading, playing, talking) - *Father involvement in routine childcare activities*: (e.g., feeding, diaper changing, giving baths) | 3-m  6-m  9-m |  | ***Paternal psychopathology***  -Dysphoria symptoms  -Anxiety symptoms  ***Maternal psychopathology***  -Dysphoria symptoms  -Anxiety symptoms | ANT  3-m  6-m  9-m |
|  | | | | | | | | | | | | |
| L = longitudinal; CS = cross-sectional; m = months; ANT = antenatally | | | | | | | | | | | | |
