# Supplementary material for: The Applicability and Performance of Tools Used to Assess the Father-Offspring Relationship in Relation to Parental Psychopathology and Offspring Outcomes
Source: Front Psychiatry. 2021 Jan 5;11:596857. doi: 10.3389/fpsyt.2020.596857 (PMC7814871; doi:10.3389/fpsyt.2020.596857)
Supplement: Supplementary file 4 [file Table_4.docx]

| **Supplementary Materials_Table 4**  Descriptive characteristics of studies utilising observational tools to assess father-infant relationship quality | | | | | | |
| --- | --- | --- | --- | --- | --- | --- |
| **Publication / Country/ Aim(s)** | **Sample details** | **Observational tool** | **Type of exposure variable(s)**  *(Paternal and /or maternal psychopathology, father-infant relationship quality)* | **Type of outcome variable(s)**  *(Father-infant relationship quality, offspring outcomes)* | **Data analysis/ Results /Limitations** | |
|  | | | | | | |
| **Observational tools (*n* = 16) extracted from *n* = 31 publications** | | | | | | |
|  | | | | | | |
| **1.Arnott & Meins (2007)**  United Kingdom  **Aim(s):** To investigate relations among parents’ attachment representations during the last trimester of pregnancy, interaction-based mind-mindedness at 6 months postpartum, and infant–parent attachment security. | **Sample**  *Recruitment:*   - 25 mothers and partners (biologically related) - Recruitment in the third trimester of pregnancy - Community sample - Extracted analyses based on 15 father-infant dyads   *Father socio-demographics*   - All of white ethnicity - Majority were of lower-middle class SES - Mean age, 36 years - 94% had higher education | **Tool extracted**  Assessment of mind-mindedness (Meins et al., 2001)  **Main tool domain:** parental mind-mindedness  *Interaction setting:*   - Location: laboratory - Set-up: free play session - Use of toys: with toys - Duration: 30-minutes - Timepoint: 6-months   *Scoring format:*   - Frequency counts   *Rater characteristics:*  -Trained researcher | **Father-infant relationship quality**  Paternal speech  *(6-months)*  Proportion of paternal mind-related comments (Assessment of mind-mindedness; Meins et al., 2001)   - Appropriate mind-related comments referring to the infant’s likely internal state - Inappropriate mind-related comments relating to the misinterpretation of the infant’s internal state | **Offspring outcomes**  Infant-attachment security  *(15-months)*  Infant attachment styles were classified during observations of infant–father strange situations  *Infant attachment styles*  -Secure attachment style  -Insecure attachment style  **Measure:** Strange Situation Procedure (SSP; Ainsworth et al., 1978) | Longitudinal data anlayses  *9-month follow-up from extracted exposure (6-months) and outcome variables (15 months)*  Results  **Father-infant relationship quality and offspring outcomes**  *Paternal speech*   - Fathers of securely attached infants (15-months) displayed higher proportions of appropriate paternal mind–related comments (6-months), compared to fathers of insecurely attached infants   (*t* (13) = 2.14, *p* < .05) (*d* = 1.15)   - Non-significant difference in the proportion of inappropriate paternal mind-related comments during father-infant interactions between attachment groups   Study limitations   - Lack of generalizability (homogenous sample, SES) - 10 father-infant dyads lost to follow-up – extracted analyses based on 15 father-infant dyads | |
|  | | | | | | |
| **2.Beal (1989)**  USA  **Aim(s):** To investigate whether demonstrating the Neonatal Behavioural Assessment Scale (NBAS) to fathers’ influences their interactions with their infants | **Sample**  *Recruitment:*  - 44 first-time fathers  - Community sample   - Fathers recruited during partners pregnancy who attended prenatal classes and present at delivery   *Father socio-demographics*  - Majority of white ethnicity  - Majority working-class  - Mean age, 28 years   - Majority had an average of 14 years of education | **Tool extracted**  Unnamed tool (Pepi, 1981)  **Main tool domain:** quantity and quality of father-infant interactions  *Interaction setting:*   - Location: home - Set-up: infant seat setting - Use of toys: with toys - Duration: 2-minutes - Timepoint: 8-weeks   *Scoring format:*  -Frequency counts  -Likert scale, 3-points  *Rater characteristics:*   - Trained paediatric student nurse (trained up to a degree of reliability, 0.85) | **Father-infant relationship quality**  Overall quality of father-infant interactions *(8-weeks)*  The frequency of paternal behaviours (i.e. visual orientation, body positioning, facial expression, vocalization and touch) per 15-second block rated as high, medium, or low quality, and matched with similar infant behaviours (i.e. visual orientation, body positioning, facial expression, vocalization) (Pepi, 1981) | **Offspring outcomes**  Infant difficult temperament  (*8-weeks)*  Fathers reported on the degree of difficult temperament of his infant (total scores)  **Measure:** Infant Characteristic Questionnaire (Bates et al., 1979) | Cross-sectional data analyses  *Extracted exposure and outcome variables measured at ~ 8 weeks*  Results  **Father-infant relationship quality and offspring outcomes**   - Non-significant association between the overall quality of father-infant interaction and paternal reports of infant difficult temperament (8-weeks)   Study limitations   - Small sample size (as reported by the authors) - Lack of generalizability (homogenous sample, SES) - Potential confounding factors not controlled for: father’s previous experience with children, his relationship with his own father, and with his wife - Researcher present during observation: potential interaction effect between parent/researcher | |
|  |  |  |  |  |  | |
| **3. Brown & Cox (2019)**  USA  **Aim(s): T**o examine relations between father-child attachment  security and both paternal sensitivity and fathers’ pleasure in  parenting. | **Sample**  *Recruitment:*   - 122 father-infant dyads   - Sample of low-middle SES    *Father socio-demographics:*   - 42% professional/semi-professional occupations - Mean age: 29-years - Mean education: 14-years - 97% European American | **Tool extracted**  National Institute of Child Health and Human Development coding scales (NICHD; NICHD Early Child Care Research Network, 2000)  **Main tool domain:** Quality of parent-infant interactions  *Interaction setting:*   - Location: home - Set-up: free-play session - Equipment: with toys - Duration: 15-minutes - Time point: 12-months   *Scoring format:*   - Likert scale: 7-points   *Rater characteristics:*  -Researcher | **Father-infant relationship quality**  Quality of father-infant interactions *(12-months)*  The degree to which parent responses are well timed and appropriate to the child's signals (NICHD)  *Paternal interactive behaviours*  -Sensitivity  *Sensitivity was examined in relation to offspring outcomes and defined as a composite score for several behaviours (i.e., sensitivity, positive regard, stimulation, dyadic mutuality, detachment)* | **Offspring outcomes**  Infant-attachment security  *(12-months)*  Infant attachment styles were classified during observations of infant–father strange situations  *Infant attachment styles*  -Secure attachment style  -Insecure attachment style  **Measure:** Strange Situation Procedure (SSP; Ainsworth et al., 1978) | Cross-sectional data analyses  *Extracted exposure and outcome variables measured at 12-months*  Results  **Father-infant relationship quality and offspring outcomes**   - Non-significant association between paternal sensitivity and infant attachment security   Study limitations   - Relatively small sample size (as reported by the authors) - Lack of generalizability (homogenous sample, SES) - Father-child relationship may differ when the father is the primary caregiver - Cross-sectional data analyses - causality cannot be inferred | |
|  |  |  |  |  |  | |
| **4. Brown, Mangelsdorf & Neff (2012)**  USA  **Aim(s):** To examine  concurrent and longitudinal associations among father involvement, paternal sensitivity, and father-child  attachment security at 13 months and 3 years of age | **Sample**  *Recruitment:*   - 115 father-infant dyads (103 dyads at 13-months, 71 dyads at 3-years).   - Community sample   - Recruitment through local community centres, retail outlets and mailing lists   *Father socio-demographics:*   - Mostly European American - 82% had higher education | **Tool extracted**  Competing demands task (Smith & Pederson, 1988)  **Main tool domain:** Paternal sensitivity  *Interaction setting:*   - Location: lab - Set-up: fathers completed questionairre whilst attending to childs needs in the same room - Use of toys: n/a - Duration: 10-minutes - Timepoint: 13-months   *Scoring format:*   - Likert scale: 5-points   *Rater characteristics:*  -Trained raters | **Relationship quality**  Paternal sensitivity  *(13-months)*  Fathers participated in a 10-minute competing demands task whereby the father completed a questionnaire while their infant  was left in the same room (Smith & Pederson, 1988) | **Offspring outcomes**  Infant-attachment security  *(13-months)*  Infant attachment styles were classified during observations of infant–father strange situations  *Infant attachment styles*  -Secure attachment style  -Insecure attachment style  **Measure:** Strange Situation Procedure (SSP; Ainsworth et al., 1978) | Cross-sectional analyses extracted  *Extracted exposure and outcome variables measured at 13-months*  Results  **Father-infant relationship quality and offspring outcomes**   - Non-significant difference in paternal sensitivity between securely and insecurely attached infants   Study limitations   - Lack of generalizability (homogenous sample, SES) - Cross-sectional data analyses - causality cannot be inferred - Exclusive reliance on sensitivity – this may overlook some aspects of fathering behaviour that could contribute to security. | |
|  |  |  |  |  |  | |
| **5.Cerniglia et al. (2014)**  Italy  **Aim(s):** To examine characteristics of mother–infant and father–infant interactions during feeding, considering the possible influence of psychological symptoms in parents, child temperament, and parental involvement**.** | **Sample**  *Recruitment:*   - 77 parents and their infant   - Community sample   - Recruited from pre-schools   *Father socio-demographics:*   - All were caucaisan - All were middle class - Mean age, 35 years - 91% were married | **Tool extracted**  Observation Scale for Mother-Infant Interactions during Feeding (SVIA; Chatoor et al., 1997)  **Main tool domain:** Quality of parent-infant feeding interactions  *Interaction setting:*   - Location: home - Set-up: lunchtime period - Use of toys: n/a - Duration: 20-minutes - Time point: 24-months   *Scoring format:*   - Likert scale: 4-points   *Rater characteristics:*  -Trained rater | **Paternal psychopathology**  Psychological symptoms  *(24-months)*  **Measure:** Symptom Checklist 90-Revised (SCL-90-R; Derogatis, 1994) | **Father-infant relationship quality**  Quality of father-infant feeding interactions  *(24-months)*  The degree to which interactive behaviours are present or absent during father-infant feeding interactions (SVIA)  *Paternal interactive behaviours*   - Interactional conflict   *Paternal affect*   - Affective state   *Infant interactive behaviours*   - Food refusal behaviours   *Dyadic affect*   - Dyadic affective state | Cross-sectional data analyses  *Extracted exposure and outcome variables both measured at 24-months*  Results  **Paternal psychopathology and father-infant relationship quality**  *Paternal interactive behaviour*   - Non-significant association between paternal psychological symptoms and paternal interactional conflict during father-infant feeding interactions (24-months) - Non-significant association between paternal psychological symptoms and paternal affective   *Infant interactive behaviour*   - Non-significant association between paternal psychological symptoms and infant food refusal behaviours   *Dyadic affect*   - Non-significant association between paternal psychological symptoms and dyadic affective state   Study limitations   - Lack of generalizability (homogenous sample, SES) - Confounding factors not controlled for: emotional/ adaptive profiles of the children, except regarding their temperaments - No objective measures of psychological symptoms or child temperament | |
|  |  |  |  |  |  |  |
| **6.Chabrol et al. (1996)**  France  **Aim(s):** To examine mother-infant and father-infant interactions in depressed and non-depressed mothers. | **Sample**  *Recruitment:*   - 20 couples and their infant - Community sample - Families recruited at obstetric clinics and a mother and baby health centre - Mothers screened for depression at recruitment (EPDS) - scores > 9 were included; of which ten met DSM-III criteria for MDD - Two groups: depressed (*n*=10), non-depressed mothers (*n*=10)   *Father socio-demographics:*  - Majority were middle-class | **Tool extracted**  Behaviour-State System (Cohn et al., 1986)  **Main tool domain:** time spent in parent and infant behavioural states  *Interaction setting:*   - Location: home - Set-up: infant-seat setting - Use of toys: yes - Duration: 2-minutes - Time point: 3-6 months   *Scoring format:*   - Time durations (secs)   *Rater characteristics:*  -Trained research assistant | **Maternal psychopathology**  Clinical diagnosis of Major Depressive Disorder based on DSM-III-R  *(3-6 months)*  **Measure:** DSM-III-R  (American Psychiatric Association, 1987) | **Father-infant relationship quality**  Quantity of paternal and dyadic interactive behavioural states  *(3-6 months)*  Time spent in paternal interactive behavioural states and the time spent in infants shared behavioural state with the father (Behaviour-State System)  *Paternal interactive behaviours*   - Elicit state - Play state   *Paternal affect*   - Negative state   *Dyadic interactive behaviours*   - Elicit/attend state - Play state   *Dyadic affect*   - Negative state - Neutral state | Cross-sectional data analyses  *Extracted exposure and outcome variables both measured between 3-6 months*  Results  **Maternal psychopathology and father-infant relationship quality**  *Paternal interactive behaviours*   - Non-significant difference in fathers time spent in an eliciting or play state during father-infant interactions between maternal diagnostic groups (3-6 months)   *Paternal affect*   - Non-significant difference in fathers time spent in a negative, state during father-infant interactions between maternal diagnostic groups   *Dyadic behaviours*   - Non-significant difference in infants time spent in a shared eliciting or play state with the father during father-infant interactions between maternal diagnostic groups   *Dyadic affect*   - Non-significant difference in infants time spent in a shared negative or neutral state with the father during father-infant interactions between maternal diagnostic groups   Study limitations   - Low participation rate – most severe cases of depression may have refused to participate - Small sample size | |
|  |  |  |  |  |  |  |
| **7. Edhborg et al. (2003)**  Sweden  **Aim(s):** To examine parent-child interactions at 15–18 months postpartum, in families with and without, maternal postnatal depressive symptoms. | **Sample**  *Recruitment:*   - 25 parents and their infants - Community sample - Recruited at 2-3 months - Mothers screened for depressive symptoms at recruitment (EPDS) - Two groups: Index group (11 families with mothers EPDS > 12), comparison group (14 families with mothers EPDS < 10)   *Father socio-demographics:*  - All fathers present at birth   - No significant differences in paternal age or SES between maternal depression groups | **Tool extracted**  Parent Child Early Relational Assessment Scale (PCERA; Clark, 1985, 1999)  **Main tool domain:** quantity and quality of parent and infant interactive behaviours  *Interaction setting:*   - Location: home - Set-up: (i) strucutral play, (ii) free-play interactions - Use of toys: with toys - Duration: 5-minutes each - Time point: 15-18 months   *Scoring format:*   - Likert scale: 5-points   *Rater characteristics:*   - Senior psychologists trained authorised Swedish trainer in the use of the PCERA | **Maternal psychopathology**  Depressive symptoms  *(2-months)*  **Measure:** Edinburgh Postnatal Depression Scale (EPDS; Cox et al., 1987) | **Father-infant relationship quality**  Paternal and infant interactive behaviour and affect  *(15-18-months)*  The quantity of paternal positive involvement behaviours and infant behaviours and mood (PCERA)  *Paternal interactive behaviours*   - Visual contact - Structuring environment - Reading the child’s cues - Mirroring - Physical positive contact   *Paternal speech:*   - Quality of verbalisation   *Paternal affect*   - Expressed positive affect - Enjoyment and pleasure   *Infant interactive behaviours*   - Alertness/interest - Quality of explorative play - Attentional abilities - Robustness - Persistence - Communicative competence - Readability   *Infant affect*   - Expressed affect - Happy/cheerful - Apathetic/withdrawn - Anxious/intense - Irritable/angry - Sober/serious   *Note: composites measures of the sub-scales, as well as the individual sub-scales were examined in relation to maternal psychopathology* | Longutudial data analyses  *13-16 month follow-up from extracted exposure (2-months) to outcome variables (15-18 months)*  Results  **Maternal psychopathology and father-infant relationship quality**  *Paternal interactive behaviours*   - Non-significant difference in overall paternal interactive behaviours during father-infant interactions, between maternal symptom level groups.   *Paternal affect*   - Fathers in the group whose partner reported higher depressive symptoms (2-months) (EPDS > 12) displayed increased levels of enjoyment and pleasure during father-infant *structured play interactions* only (15-18 months) (*F* = 7.39, *p* <0.02), compared to fathers in the group whose partner reported lower symptoms (EPDS < 10).   *Infant interactive behaviours*   - Non-significant difference in overall infant interactive behaviours during father-infant interactions, between maternal symptom level groups   *Infant affect*   - Fathers in the group whose partner reported higher depressive symptoms (2-months) had infants who displayed lower levels of negative affect when interacting with their father during *structured play* interactions only (15-18 months) (*F* = 4.16, *p* = 0.05), compared to infants of fathers whose partner reported lower symptoms. - Non-significant difference in overall infant interactive affect during father-infant *free play interactions*, between maternal symptom level groups.   Study limitations   - Measurement of paternal depression was not reported - Maternal depression reported at 2-months | |
|  |  |  |  |  |  |  |
| **8. Eiden, Colder, Edwards & Leonard (2009)**  USA  **Aim(s):** To examine a conceptual model predicting children's social competence in a sample of children with alcoholic and non-alcoholic parents | **Sample**  *Recruitment:*   - 227 parents and their 12-month old infants - Community sample - Biological parents - All parents screened at recruitment for alcohol dependence use (self-report) - Three groups: 96 families with one parent with alcohol problems (including 90 fathers), 97 where both parents had alcohol problems and a control group   *Father socio-demographics:*   - 55% completed post high-school education or a college degree - 87% Caucasian - Mean age: 33-years | **Tool extracted**  Parent Child Early Relational Assessment Scale (PCERA; Clark 1985, 1999)  **Main tool domain:** Quality of parent and infant interactive behaviours  *Interaction setting:*   - Location: home - Set-up: free-play session - Use of toys: with toys - Duration: 10-minutes - Time point: 24-months   *Scoring format:*   - Likert scale: 5-points   *Rater characteristics:*   - Trained raters | **Paternal psychopathology**  Alcohol abuse and dependence  (*12, 18-months)*  **Measure:** DSM-IV (American Psychiatric Association, 1994)  Depressive symptoms  *(12, 18-months)*  **Measure:** CES-D (Radloff, 1977)  **Maternal psychopathology**  Depressive symptoms  *(12, 18-months)*  **Measure:** CES-D (Radloff, 1977) | **Father-infant relationship quality**  Paternal and infant interactive behaviour and affect  *(24-months)*  The quality of paternal interactive behaviours and affect (PCERA)  *Paternal interactive behaviours*   - Sensitivity - Warmth   *Paternal affect*   - Negative affect   *Note: composites measures of the sub-scales, as well as the individual sub-scales were examined in relation to outcome variables*  **Offspring outcomes**  Child self-regulation  *(24, 36-months)*  An observational  measure of the internalization of paternal rules.  **Measure:** three tasks developed  by Kochanska, Murray, Jacques, Koenig, and Vandegeest (1996)  and Kochanska and Knaack (2003)  Child externalizing problems  *(24, 36-months)*  Parent rated their child externalising behaviour problems  **Measure:** Child Behavior Checklist (Achenbach, 1992) | Cross-sectional and longitudinal data analyses  *Up to 12-month follow-up from extracted exposure (12, 18-mionths) and outcome variables (24-months)*  Results  **Paternal psychopathology and father-infant relationship quality**  *Paternal interactive behaviours*   - Increased levels of paternal depressive symptoms (12-18 months) were associated with lower levels of paternal warmth (24-months) (*r* = *-*.14, *p* = < .05) - Presence of paternal alcohol dependence (12-18 months) was associated with lower levels of warmth/ sensitivity (24-months) (*β* = -.30, *p* = < .01) - Non-significant association between paternal depressive symptoms (12-18 months) and sensitivity (24-months)   *Paternal affect*   - Non-significant association between paternal depressive symptoms (12-18 months) and paternal negative affect (24-months)   **Maternal psychopathology and father-infant relationship quality**  *Paternal interactive behaviours*   - Increased levels of maternal depressive symptoms (12-18 months) were associated with lower levels of paternal sensitivity (*r* = *-*.19, *p* = < .05) and warmth (*r* = *-*.22, *p* = < .05) at 24-months   *Paternal affect*   - Increased levels of maternal depressive symptoms (12-18 months) were associated with lower levels of paternal negative affect (*r* = -.21, *p* = < .05) at 24-months   **Father-infant relationship quality and offspring outcomes**   - Non-significant association between paternal interactive behaviours (i.e., sensitivity and warmth) and affect (i.e., negative affect) and child self-regulation (24-months) and externalising behavioural problems (24, 36-months) (*Bivariate associations extracted)*   Study limitations   - Generalizability of results may be limited to the population of higher functioning families who may be more likely to respond to open letters of recruitment about participation in research - Role of maternal alcohol problems could not be examined independent of fathers’ alcohol | |
|  |  |  |  |  |  |  |
| **9. Eiden Chavez & Leonard (1999)**  USA  **Aim(s):** To examine the relationship between fathers’ alcoholism and the quality  of parent–infant interactions during free play. | **Sample**  *Recruitment:*   - 204 parents and their infant - Two groups: 104 alcoholic fathers, 100 controls - Recruited from New York State birth records   *Father socio-demographics*  - Most of middle-class SES  - 89% Caucasian  - 55% post-high school education or completed a college degree  - 88% married  - Mean age, 33-years | **Tool extracted**  Parent Child Early Relational Assessment Scale (PCERA; Clark, 1980) – *unpublished version*  **Main tool domain:** quality of parent and infant interactive behaviours  *Interaction setting:*   - Location: lab - Set-up: free play session - Use of toys: with toys - Duration: 5-minutes - Time point: 12-months   *Scoring format:*   - Likert scale: 5-points   *Rater characteristics:*  -Trained coders | **Paternal psychopathology**  Alcohol abuse and dependence  (*12-months)*  **Measure:** self-report  instrument based on the UM-CIDI interview (Anthony et al., 1994; Kessler et al.,  1994)  Depressive symptoms  *(12-months)*  **Measure:** CES-D (Radloff, 1977) | **Father-infant relationship quality**  Paternal and infant interactive behaviour and affect  *(12-months)*  The quality of paternal and infant interactive behaviours and affect (Clark et al., 1980)  *Paternal interactive behaviours*   - Sensitivity   *Paternal speech:*   - Verbalisation   *Paternal affect*   - Positive affect - Negative affect   *Infant interactive behaviours*   - Responsiveness   *Infant affect*   - Positive affect - Negative affect   **Offspring outcomes**  Infant fussy-difficult temperament  *(12-months)*  Fathers reported on the degree of difficult temperament of his infant  **Measure:** ICQ (Bates et al., 1979) | Cross-sectional data analyses  *Extracted exposure and outcome variables assessed at 12-months*  Results  **Paternal psychopathology and father-infant relationship quality**  *Paternal interactive behaviours*   - Fathers in the alcoholic group displayed lower levels of sensitivity towards their infant during father-infant interactions, compared to fathers in the control group   (*p* = .008, *d* = 0.38)   - Increased levels of paternal depressive symptoms were associated with lower levels of paternal sensitivity   (*r* = -.18, *p* < .01)  *Paternal speech*  Fathers in the alcoholic group displayed lower levels of verbalisation towards their infant during father-infant interactions, compared to fathers in the control group  (*p* = .003, *d* = 0.42)   - Non-significant association between paternal levels of verbalisation and paternal depressive symptoms   *Paternal affect*  Fathers in the alcoholic group displayed lower levels of affect (i.e., positive and negative affect) towards their infant during father-infant interactions, compared to fathers in the control group (positive: *p* = .007, *d* = 0.38; negative: *p* = 0.48, *d* = 0.28)   - Non-significant association between paternal levels of affect (i.e., positive and negative affect) and paternal depressive symptoms   *Infant interactive behaviours*   - Infants of fathers in the alcoholic group displayed lower levels of responsiveness towards their father during father-infant interactions, compared to infants of fathers in the control group   (*p* = .045, *d* = 0.26)   - Non-significant association between infant responsiveness and paternal depressive symptoms   *Infant affect*   - Non-significant association between infant affect (i.e., positive and negative affect) and paternal alcohol status or depressive symptoms   **Father-infant relationship quality and offspring outcomes**   - Non-significant association between paternal interactive behaviours, speech and affect and infant fussy-difficult temperament   Study limitations   - Generalizability of results may be limited to the population of higher functioning families who may be more likely to respond to open letters of recruitment about participation in research - Utilization of self-report data with respect to parental alcohol problems and psychopathology - Cross-sectional data analyses - causality cannot be inferred | |
|  |  |  |  |  |  |  |
| **10.Feldman & Eidelman (2007)**  Israel  **Aim(s):** To examine relations between maternal postpartum behaviour and the emergence of parent-infant relatedness as a function of infant autonomic maturity in premature infants and full-term infants. | **Sample**  *Recruitment:*   - 108 parents and their infant - Community sample - Families recruited at nursery (term) and neonatal units (pre-term) - Two groups: 56 pre-term infants, 52 term infants   *Father socio-demographics:*  - All middle-class SES  - Majority had an average of 14-years education  - 107 families employed  - 86% had full employment  - All were married to partner   - Mean age, 32 years | **Tool extracted**  Unnamed tool (Feldman & Eidelman, 2007)  **Main tool domain:** quantity of parent interactive behaviours and dyadic synchrony  *Interaction setting:*   - Location: home - Set-up: free play session - Use of toys: without toys - Duration: 5-minutes - Time point: 3-months   *Scoring format:*   - Frequency counts / proportions   *Rater characteristics:*  -Trained research assistant | **Maternal psychopathology**  Depressive symptoms  *(early neonatal period)*  **Measure:** Beck Depression Inventory (BDI; Beck, 1978) | **Father-infant relationship quality**  Quantity of paternal interactive behaviours and dyadic synchrony *(3-months)*  The proportion of paternal behaviours and time spent in synchronized social gaze with the infant (Unnamed, Feldman et al., 2004)  *Paternal interactive behaviours*   - Father affectionate touch   *Dyadic interactive behaviours*   - Father-infant gaze synchrony | Longitudinal data analyses  *2-3 month follow-up from extracted exposure (neonatal period) and outcome variables (3-months)*  Results  **Maternal psychopathology and father-infant relationship quality**  *Paternal interactive behaviours*   - Non-significant association between maternal depressive symptoms (neonatal period) and fathers affectionate touch (3-months) *(total sample, bivariate analyses reported)*   Study limitations:   - Lack of generalizability (homogenous sample, SES) - No objective measure of depressive symptoms | |
|  |  |  |  |  |  | |
| **11. Fuertes et al., (2016)**  Portugal  **Aim(s):** To evaluate maternal and paternal contributions to infant–mother and  infant–father attachment at 12 and 18 months in an understudied  Portuguese sample. | **Sample**  *Recruitment:*   - 82 parents and their infant - Recruited at the Santo Espírito Hospital, Azores, Portugal.     *Father socio-demographics:*   - 16% had completed higher college education - All fathers had full-time occupations - Majority were married | **Tool extracted**  CARE-Index (infant form; Crittenden, 2003)  **Main tool domain:** quality of parent-infant interactions  *Interaction setting:*   - Location: home - Set-up: free-play session - Use of toys: with toys - Duration: 5 mins - Time point: 9, 15-months   *Scoring format:*   - Likert scale: 14 points   *Rater characteristics:*  -Trained coder | **Father-infant relationship quality**  Quality of father-infant interactions *(9-months)*  The quality of parent and infant behaviours (CARE-Index)  *Paternal interactive behaviours*  -Sensitivity  -Control  *Infant interactive behaviours*  -Passivity | **Offspring outcomes**  Infant-attachment security  *(12, 18-months)*  Infant attachment styles were classified during observations of infant–father strange situations  *Infant attachment styles*  -Secure attachment style  -Insecure attachment style  **Measure:** Strange Situation Procedure (SSP; Ainsworth et al., 1978) – adapted version | Longitudinal data analyses  *3-9 month follow-up from extracted exposure (9-months) and outcome variables (12, 18-months)*  Results  **Father-infant relationship quality and offspring outcomes**  *Paternal interactive behaviours*   - Fathers of securely attached infants displayed increased levels of paternal sensitivity (*p* < .01) and lower levels of paternal control (*p* < .05) during father-infant interactions (9-months), compared to fathers of insecurely attached infants at 12-months. - Paternal sensitivity at 9-months predicted infant secure attachment towards the father at 12-months (logistic regression model; *β =* 16.84, CI = 0.000 – 0.001) - Non-significant association between paternal interactive behaviours (i.e., sensitivity and control) (9-months) and infant attachment security at 18-months   *Infant interactive behaviours*   - Non-significant association between infant passivity during father-infant interactions and infant attachment security at 12-months   Study limitations   - Potentially lack of generalizability across all populations - Relatively small sample size | |
|  |  |  |  |  |  | |
| **12.Goodman (2008)**  USA  **Aim(s):** To examine the influence of maternal postpartum depression on fathers and identified maternal and paternal factors associated with father–infant interaction in families with depressed as compared with nondepressed mothers. | **Sample**  *Recruitment:*   - 128 parents and their infant - Community sample - Maternal sample screened for depressive symptoms – with the aim to include ~50% of mothers scoring over the cut-off for suspected mild or major depression (EPDS >10) - Recruited during stay at postpartum units of a teaching hospital   *Father socio-demographics:*   - 88% had higher education - 83% were of white ethcniciy, 6% of black ethnicity - 90% were in full-time work | **Tool extracted**  Nursing Child Assessment Teaching Scale (NCATS; Sumner & Spietz, 1994)  **Main tool domain:** quality of parent-infant interactions  *Interaction setting:*   - Location: home - Set-up: structured play, parents teach their children an age-appropiate skill - Use of toys: n/a - Duration: 1-5 mins (based on time to complete tasks) - Time point: 2-3 months   *Scoring format:*   - Binary rating: presence / absence of behaviour   *Rater characteristics:*   - Trained researcher (completed comprehensive training and successful attainment of at least 90% reliability for research certification) | **Paternal psychopathology**  Depressive symptoms  *(2-3 months)*  **Measure:** EPDS (Cox et al., 1987)  **Maternal psychopathology**  Depressive symptoms  *(2-3 months)*  **Measure:** EPDS (Cox et al., 1987) | **Father-infant relationship quality**  Synchronous father-infant interactions *(2-3 months)*  The presence or absence of the dyads ability to participate in synchronous mutual interaction (NCATS)  *Paternal interactive behaviours*  *-*Sensitivity to infant cues  -Sooth distressed infant  -Communicate warmth  -Fostering opportunities  *Infant interactive behaviours*   - Ability to provide clear cues - Ability to respond to fathers’ communicative attempts   *Total scores of father-infant interactions (composite of father and infant domains) were examined in relation to psychopathology* | Cross-sectional data analyses  *Extracted exposure and outcomes both measured between 2-3 months*  Results  **Paternal psychopathology and father-infant relationship quality**   - Increased paternal depressive symptoms (EPDS total scores) were associated with lower overall scores in father-infant interactions (*r* = -.22, *p* < .05) *(bivariate analyses reported)*   **Maternal psychopathology and father-infant relationship quality**   - Fathers in the group whose partner reported higher depressive symptoms (EPDS > 10) displayed lower overall scores (i.e., less optimal) in father-infant interactions, compared to fathers in the group whose partner reported lower symptoms (EPDS < 10)   (*β* = -.26, *p* < .05)   - Increased maternal depressive symptoms (EPDS total scores) were associated with lower overall scores in father-infant interactions (*r* = -.32, *p* < .001) *(bivariate analyses reported)*   Study limitations:   - Lack of generalizability (homogenous sample, SES) - No objective measure of psychopathology - Unclear whether mothers were depressed prenatally | |
|  |  |  |  |  |  | |
| **13.Hall et al. (2014)**  Netherlands  **Aim(s):** To examine whether the quality of early paternal representations is associated with later quality of paternal and infant interactive behavior, and whether paternal interactive behavior mediates the relation between paternal representations and infant's development. | **Sample**  *Recruitment:*   - 220 parents and their infants - Recruited at local hospitals - Two groups: 71-full term infants, 118 preterm infants - Extracted analyses based on 150 fathers (62 term, 88 pre-term infants)   *Father socio-demographics:*   - 77% had completed higher secondary education - Mean age, 35-years - 64% were first-time fathers | **Tool extracted**  National Institute of Child Health and Human Development coding scales (NICHD; NICHD Early Child Care Research Network, 1999)  **Main tool domain:** quality of parent-infant interactions  *Interaction setting:*   - Location: home - Set-up: free-play session - Equipment: with toys - Duration: 15-minutes - Time point: 24-months   *Scoring format:*   - Likert scale: 4-points   *Rater characteristics:*   - Trained coder (standardized training with practice tapes until 80% reliability was reached with a second coder) | **Father-infant relationship quality**  ***Mediator variable:***  Quality of father-infant interactions *(24-months)*  The degree to which parent responses are well timed and appropriate to the child's signals (NICHD)  *Paternal interactive behaviours*  -Sensitivity  -Intrusiveness  -Withdrawal  Paternal attachment representations of the infant  *(6-months)*  Fathers report on their subjective experiences and perceptions of their child, caregiving behaviour and relationship with child (WMCI)  *Representation classifications*  -Balanced representations  -Disengaged representations  -Distorted representations | **Offspring outcomes**  Infant language development  *(24-months)*  **Measure:** Peabody Picture Vocabulary Test **(**PPVT; Dunn & Dunn, 2004) | Longitudinal data analyses  *18-month follow-up from extracted exposure (6-months) to outcome variables (24-months)*  Results  **Father-infant relationship quality and offspring outcomes**  **.**  *Paternal interactive behaviours*   - Paternal sensitivity, intrusiveness, and withdrawal (24-months) mediated the association between   paternal balanced representations of their infant (6-months) and higher scores in infant vocabulary (24-months)  (*β* = 1.22; CI = 0.05, 2.94)  Study limitations:   - Lack of generalizability (homogenous sample, SES) - Attrition at first measurement (6-months), 14% of fathers had dropped out - A further 39 fathers were lost to follow-up at 24-months. - Extracted analyses based on 150 fathers (62 term, 88 pre-term infants) | |
|  |  |  |  |  |  | |
| **14.Koch et al. (2019)**  Brazil  **Aim(s):** To investigate whether recognition of facial expressions acts as a mediator of the effects of postpartum depression on father–infant interactions. | **Sample**  *Recruitment:*   - 64 couples and their infant - Community sample - Recruitment, maternity wards, health centres - Fathers screened at recruitment - inclusion positive SCID and/or BDI (≥15) EPDS (≥10) - Two groups: 17 depressed, 44 control fathers (3 fathers were lost to follow-up)   *Father socio-demographics:*   - Mean age 32-35 years - Majority had completed secondary education or above and were in employment | **Tool extracted**  Global Rating Scales (GRS; Gunning & Murray, 2002)  **Main tool domain:** quality of parent-infant interactions  *Interaction setting:*   - Location: home - Set-up: free-play interaction or infant-seat setting - Use of toys: with or w/out - Duration: 5-minutes - Time point: 2-16 weeks   *Scoring format:*   - Likert scale: 5-points   *Rater characteristics:*  -Researcher | **Paternal psychopathology**  Clinical diagnosis of major depressive disorder  *(2-16 weeks)*  **Measure:** DSM-IV-R (SCID; First et al., 1997) | **Father-infant relationship quality**  Quality of father-infant interactions *(2-16 weeks)*  The quality of paternal, infant, and dyadic behaviours (GRS)  *Paternal interactive behaviours*  -Sensitivity  -Responsiveness  *Paternal affect*  -Depressive affect  *Infant interactive behaviours*  -Attention to father  *Infant affect*  -Negative affect  *Dyadic behaviours*  -Quality of dyadic interaction | Cross-sectional data analyses  *Extracted exposure and outcomes measured at the same time between 2 and 16-weeks*  Results  **Paternal psychopathology and father-infant relationship quality**  *Paternal interactive behaviours*   - Fathers in the clinically depressed group displayed lower mean scores in responsive and sensitive behaviours during father-infant interactions, compared to non-depressed fathers (2-16 weeks) (*p* < 0.05)   *Paternal affect*   - Fathers in the clinically depressed group displayed higher scores in depressive affect during father-infant interactions, compared to non-depressed fathers (2-16 weeks) (*p* < 0.05)   *Infant interactive behaviours*   - Fathers in the clinically depressed group had infants who displayed lower mean scores in attention towards the father during father-infant interactions, compared to infants of non-depressed fathers (2-16 weeks) (*p* < 0.05)   *Infant affect*   - Non-significant difference in infant negative affect between paternal diagnostic groups   *Dyadic behaviours*   - Non-significant difference in the quality of dyadic interaction between paternal diagnostic groups   Study limitations:   - Lack of generalizability (homogenous sample, SES) - Maternal depression was not examined in relation to fathers-interactions | |
|  |  |  |  |  |  | |
| **15. Lucassen et al. (2017)**  Netherlands  **Aim(s):** To explore the relation between lifetime prevalence of depression or anxiety in fathers with infant–father attachment security and mediating roles of sensitivity and perceived family stress. | **Sample**  *Recruitment:*  - 94 father-infant dyads   - Community sample of primary caregivers - Recruited during pregnancy - Fathers screened for history or current depression or anxiety (clinical measure)   *Father socio-demographics:*   - Mean age, 35 years - 74% had higher education | **Tool extracted**  Ainsworth 9-point rating scales for sensitivity and cooperation (AMSS; Ainsworth, et al. 1974)  **Main tool domain:** parental sensitivity  *Interaction setting:*   - Location: laboratory - Set-up: free-play session - Use of toys: without toys - Duration: 5-minutes - Time point: 14-months   *Scoring format:*  -Likert scale: 9-points  *Rater characteristics*  - Coders trained to  reliability (no cut-off  statistic, n/r) | **Paternal psychopathology**  History or current depression or anxiety disorder  *(measured antenatally)*  **Measure:** Composite International Diagnostic Interview (CIDI; WHO, 1990)  ***Mediator variable:***  **Father-infant relationship quality**  Paternal sensitivity  *(14-months)*  Paternal ability to perceive the infant's signals accurately and respond promptly and appropriately (AMSS)  *Paternal interactive behaviours*  -Sensitivity  -Cooperation | **Offspring outcomes**  Infant-attachment security  *(14-months)*  Infant styles of attachment during of infant–father strange situations were classified using continuous scores based on the interactive scales (Richters et al., 1988)  **Measure:** Strange Situation Procedure (SSP; Ainsworth et al., 1978)  *Total score for infant attachment was examined in relation to the father-infant relationship* | Cross-sectional and longitudinal data analsyes  *Extracted exposure measured antenatally and outcome/mediator variables at 14-months*  Results  **Paternal psychopathology and father-infant relationship quality** *(longitudinal association)*   - Non-significant association between paternal history of depression/anxiety and paternal sensitivity (14-months) - Non-significant association between paternal antenatal depression/anxiety and paternal sensitivity (14-months)   **Father-infant relationship quality and offspring outcomes** *(concurrent association)*   - Non-significant association between paternal sensitivity and infant attachment scores (14-months) - Non-significant mediation effect of paternal sensitivity (14-months) on the association between paternal history of depression/anxiety and infant-attachment security (14-months)   Study limitations:   - Lack of generalizability (homogenous sample, SES) - Fathers primary caregiver - e.g., highly involved fathers | |
|  |  |  |  |  |  | |
| **16. Lundy (2003)**  USA  **Aim(s):** To explore the relations among fathers’ and mothers’ appropriate mind-related comments during interactions with their 6-month-old infants, and subsequent infant attachment security. | **Sample**  *Recruitment:*   - 24 parent couples - Community sample - Recruitment through local paediatricians, day care facilities, newspaper adverts and an infant subject pool   *Socio-demographics:*   - Mean age, 30 years (father) - Majority of white ethnicity - Mostly lower-middle SES - 40% college educated | **Tool extracted**  Assessment of mind-mindedness (Meins et al., 2001) (modified version)  **Main tool domain:** parental mind-mindedness  *Interaction setting:*   - Location: laboratory - Set-up: infant-seat setting - Use of toys: with toys - Duration: 6-minutes - Timepoint: 6-months   *Scoring format:*   - Frequency counts   *Rater characteristics:*  -Trained researcher | **Paternal psychopathology**  Depressive symptoms  *(6-months)*  **Measure:** CES-D (Radloff, 1977) | **Father-infant relationship quality**  Paternal speech *(6-months)*  Proportion of paternal mind-related comments (Assessment of mind-mindedness; Meins et al., 2001)  *Paternal speech:*   - Thoughts, knowledge, desires comments - Problem solving comments - Emotional engagement comments   **Offspring outcomes**  Infant attachment security  *(13-months)*  Parents perception of infant behaviours which are characteristics of the infant related to attachment security (AQS; Waters, 1987) | Cross-sectional and longitudinal data analsyes  *Up to 7-month follow-up from extracted exposure (6-months) to outcome variable (6 and 13-months)*  Results  **Paternal psychopathology and father-infant relationship quality**   - Non-significant association between paternal depressive symptoms and mind-related comments (i.e., thought processes, emotional engagement and problem solving) (6-months)   **Father-infant relationship quality** **and offspring outcomes**   - Increased comments related to the infants thought processes (6-months) were associated with higher infant attachment scores (13-months) (*r =* .64, *p* < .005). - Increased comments related to the infants emotional engagement (6-months) were associated with higher infant attachment scores (13-months) (*r* = .41, *p* = .06; marginal). - Non-significant association between problem solving comments and infant attachment security   Study limitations:   - Lack of generalizability (homogenous sample, SES) - Relatively small sample size - No objective measure of psychopathology | |
|  |  |  |  |  |  | |
| **17. Magill-Evans & Harrison (1999)**  Canada  **Aim(s):** To identify the factors (mother’s interactions, mother’s perceptions of  parenting stress, father’s interactions, father’s perceptions of parenting stress, child’s interactions with each parent, whether the child was born preterm,  and family SES) or combination of factors that predicted  the development of healthy preterm and full-term children | **Sample**  *Recruitment:*   - 103 two-parent families - Two groups: 49 preterm (PT) and 54 fullterm (FT) infants) - Recruitment part of a wide longitudinal study following children from birth to 4-y   *Paternal socio-demographics:*   - Mean age, 32.3 – 31.5 years (preterm, full-term groups) - Education in years: 14.4 – 15.2 years (PT, FT groups) | **Tool extracted**  Nursing Child Assessment Teaching Scale (NCATS; Sumner & Spietz, 1994)  **Main tool domain:** quality of parent-infant interactions  *Interaction setting:*   - Location: home - Set-up: structured play, parents teach their children an age-appropiate skill - Use of toys: n/a - Duration: 1-5 mins (based on time to complete tasks) - Time point: 3, 12-months   *Scoring format:*   - Binary rating: presence / absence of behaviour   *Rater characteristics:*   - Coders trained until attainment of at least 85% agreement | **Father-infant relationship quality**  Quality of father-infant interactions *(3, 12-months)*  The degree to which parent responses are well timed and appropriate to the child's signals (NCATS)  *Paternal interactive behaviours*  *-*Sensitivity to infant cues  -Sooth distressed infant  -Communicate warmth  -Fostering opportunities  *Infant interactive behaviours*   - Ability to provide clear cues - Ability to respond to fathers’ communicative attempts   *Total scores of father-infant interactions (composite scores of father domains and infant domains) were examined in relation to outcomes* | **Offspring outcomes**  Infant mental development  *(18-months)*  **Measure:** Mental scale of the Bayley Scales of Infant Development (Bayley, 1969)  Infant receptive language  *(18-months)*  **Measure:** The Receptive Communication Age of the Sequenced Inventory of Communication  Development-Revised (SICD-R) (Hedrick, Prather, & Tobin,  1984**)**  Infant expressive language  *(18-months)*  **Measure:** MacArthur Communicative Development Inventory (MCDI) (Fenson et al., 1991) | Longitudinal data analsyes  *Up to 15-month follow-up from extracted exposure (3,12-months) to outcome variable (18-months)*  Results  **Father-infant relationship quality** **and offspring outcomes**   - Higher quality father-infant interactions at 3-months (total paternal NCATS scores, total sample) was a significant predictor of child receptive language at 18-months (increased scores; *β* = .19, *p* < .05) - Non-significant association between the quality of father-infant interactions (father and infant total scores) (12-months) and child receptive language at 18-months - Non-significant association between the quality of father-infant interactions (father and infant total scores) (3, 12-months) and child scores on Bayley Mental Development scale and expressive language development (18-months)   Study limitations:   - Relatively small sample size - Selective attrition rate – with more families of preterm infants withdrawing from the project - Child development assessed at 18-months - subtle differences in may not be apparent until preschool age or above | |
|  |  |  |  |  |  | |
| **18. Miller et al., (2019)**  USA  **Aim(s):** To examine mechanisms that account for associations between parents’ early mind-mindedness and children’s future attachment security, using robust behavioral measures. | **Sample**  *Recruitment:*   - 102 two-parent families - Community sample - Biological parents cohabiting   *Father socio-demographics:*   - 20% fathers post-graduate education - 84% of White ethnic background | **Tool extracted (1)**  Assessment of mind-mindedness (Meins et al., 2015)  **Main tool domain:** parental mind-mindedness  *Interaction setting:*   - Location: Home - Set-up: snack period and free play session - Use of toys: with toys - Duration: 7, 6-mins - Timepoint: 7-months   *Scoring format:*   - Frequency counts   *Rater characteristics:*  -Trained raters | **Father-infant relationship quality**  Paternal speech *(7-months)*  Proportion of paternal mind-related comments (Assessment of mind-mindedness; Meins et al., 2015)   - Appropriate mind-related comments referring to the infant’s likely internal state - Non-attuned mind-related comments | **Offspring outcomes**  Infant attachment security  *(24-months)*  Parents perception of infant behaviours which are characteristics of the infant related to attachment security (AQS; Waters, 1987)  Child attachment security  *(10-years)*  Assesses parent-child attachment in middle childhood, based on lengthy observations of the parent-child dyad, interacting across diverse contexts  **Measure:** Iowa Attachment Behavioral Coding (IABC; Boldt, Kochanska, Grekin, & Brock, 2016). | Longitudinal data analsyes  *Up to 9.5-year follow-up from extracted exposure (7-months) to outcome variables (24-months, 10-years).*  Results  **Father-infant relationship quality and offspring outcomes**   - Increased paternal mind-minded (7-months) appropriate comments were associated with more secure offspring attachment security at 24-months (*r* = .30, *p* < .01) and 10-years (*r* = .25, *p* < .05) - Non-significant association between non-attuned mind-related comments (7-months) and offspring attachment security - 24-months, 10-years   Study limitations:   - Relatively small sample size (as reported by the authors) - Lack of generalizability (homogenous sample, SES) - Attachment security assessed through parent report – no objective measures | |
|  |  |  |  |  |  | |
| **19. Mills-Koonce et al., (2015)**  USA  **Aim(s):** To examine associations between maternal and paternal sensitive parenting and child cognitive development across the first 3 years of life | **Sample**  *Recruitment:*   - 1292 parents and their infant - Biological parents - Part of a wider longitudinal study, Family Life project - Sample weighted towards low-income families and African American families   *Father socio-demographics:*  Father socio-demographics n/r | **Tool extracted**  National Institute of Child Health and Human Development coding scales (NICHD; Early Child Care Research Network, 1999)  **Main tool domain:** Quality of parent-infant interactions  *Interaction setting:*   - Location: home - Set-up: free-play and semi-structured play - Equipment: with toys - Duration: 10-minutes - Time point: 6, 24-months   *Scoring format:*   - Likert scale: 5, 7-points   *Rater characteristics:*  -Trained raters (until reliabity met at ICC > .80) | **Father-infant relationship quality**  Quality of father-infant interactions *(6, 24-months)*  The degree to which parent responses are well timed and appropriate to the child's signals (NICHD)  *Paternal interactive behaviours*  -Cognitive stimulation  - Detachment  - Animation in interaction  *Paternal affect*  - Positive regard  *Note:* individual subscales were composited to form an overall measure of paternal sensitive parenting | **Offspring outcomes**  Child cognitive development  *(6, 15-months)*  **Measure:** The Mental scale of the BSID-II (Bayley,  1993)  Child cognitive development  *(36-months)*  **Measure**: Wechsler Preschool and Primary Scales of Intelligence (WPPSI; Wechsler, 2002). | Longitudinal data analsyes  *Up to 30-month follow-up from extracted exposure (6 and 24-months) to outcome variables (6, 15, 36-months).*  Results  **Father-infant relationship quality and offspring outcomes**   - Non-significant direct effects between early paternal sensitive parenting (6-months) and early child cognitive development (6, 15-months) - Non-significant direct effects between later paternal sensitive parenting (24-months) and later child cognitive development (36-months)   Study limitations:   - Lack of generalizability (little representation of step-fathers and non-resident fathers) - Inclusion of biological mothers’ and fathers’ – gene-environment may account for variations in cognitive outcomes | |
|  |  |  |  |  |  | |
| **20.Parfitt et al. (2013)**  United Kingdom  **Aim(s):** To examine the effect of fathers’ and mothers’ pre and postnatal mental health on mother–infant and father–infant interactions. | **Sample**  *Recruitment:*   - 44 mothers, 40 fathers   (from 45 families expecting their first child)   - Community sample - Recruited antenatally through prenatal classes   *Father socio-demographics:*   - Mean age, 35 years - 94% had higher education - 64% were married - Marjority were caucasian | **Tool extracted**  CARE-Index (infant form; Crittenden, 2004)  **Main tool domain:** quality of parent-infant interactions  *Interaction setting:*   - Location: home - Set-up: free-play session - Use of toys: with toys - Duration: 3-5 mins - Time point: 3-months   *Scoring format:*   - Likert scale: 7-14 points   *Rater characteristics:*  -Trained reliable coder | **Paternal psychopathology**  Depressive and anxiety symptoms *(antenatally and at 3-months)*  **Measure:** The Hospital Anxiety and Depression Scale (HADS; Zigmond & Snaith, 1983)  Post-traumatic stress symptoms *(3-months)*  **Measure:** The Post-traumatic Stress Diagnostic Scale (PDS; Foa, Cashman, Jaycox, & Perry, 1997)  **Maternal psychopathology**  Depressive and anxiety symptoms *(antenatally and 3-months)*  **Measure:** HADS (Zigmond & Snaith, 1983)  Post-traumatic stress symptoms *(3-months)*  **Measure:** PDS (Foa, Cashman, Jaycox, & Perry, 1997) | **Father-infant relationship quality**  Quality of father-infant interactions *(3-months)*  The quality of parent and infant behaviours (CARE-Index)  *Paternal interactive behaviours*  -Sensitivity  -Controlling  -Unresponsiveness  *Infant interactive behaviours*  -Difficultly  -Passivity | Cross-sectional and longitudinal data analyses  *Up to 3-month follow-up from exposure (antenatally and at 3-months) and outcome variables (3-months)*  Results  **Paternal psychopathology and father-infant relationship quality**  *Paternal interactive behaviours*   - Increased paternal antenatal depressive and anxiety symptoms (total score; HADS) were associated with lower scores in paternal controlling behaviours (*β* = -.66, *p < .*001), and higher scores in unresponsiveness (*β* = .54, *p < .*01) during father-infant interactions (3-months) - Non-significant association between paternal antenatal depressive and anxiety symptoms (total score; HADS) and paternal sensitivity during father-infant interactions (3-months) - Non-significant association between paternal postnatal depressive and anxiety symptoms at 3-months (total score; HADS) and paternal behaviours during father-infant interactions (3-months) - Non-significant association between paternal postnatal PTSD symptoms at 3-months and paternal behaviours during father-infant interactions (3-months)   *Infant interactive behaviours*   - Increased paternal antenatal depressive and anxiety symptoms (total scores; HADS) were associated with lower scores in infant difficulty (*β* = -.38, *p < .*05), and higher scores in infant passivity (*β* = .49, *p < .*01) during father-infant interactions (3-monhts) - Increased paternal postnatal depressive and anxiety symptoms at 3-months (total scores; HADS) were associated with increased scores in infant difficulty (*β* = .61, *p < .*01) during father-infant interactions (3-months) - Non-significant association between paternal postnatal depressive and anxiety symptoms at 3-months (total scores; HADS) and infant passivity father-infant interactions (3-months) - Non-significant association between paternal postnatal PTSD symptoms at 3-months and infant behaviours during father-infant interactions (3-monhts)   **Maternal psychopathology and father-infant relationship quality**  *Paternal interactive behaviours*   - Increased maternal postnatal depressive and anxiety symptoms at 3-months (total score; HADS) were associated with lower scores in paternal controlling behaviours (*β* = -45., *p < .*05), and higher scores in unresponsiveness (*β* = .52, *p < .*05) during father-infant interactions (3-months) - Non-significant associations between maternal antenatal depressive and anxiety symptoms (total score; HADS) and paternal behaviours during father-infant interactions (3-months) - Non-significant associations between maternal postnatal PTSD symptoms at 3-months and paternal behaviours during father-infant interactions (3-months)   *Infant interactive behaviours*   - Non-significant associations between maternal antenatal or postnatal depressive and anxiety symptoms (total score; HADS) and infant behaviours during father-infant interactions (3-months) - Non-significant associations between maternal postnatal PTSD symptoms and infant behaviours during father-infant interactions (3-months)   Study limitations   - Lack of generalizability (homogenous sample, SES) - Parents generally reported low depressive and anxiety symptoms – generalizability to clinical samples with caution. | |
|  |  |  |  |  |  | |
| **21. Ramchandani et al. (2013)**  United Kingdom  **Aim(s):** To examine whether father–infant interactions at age 3 months independently predict child behavioural problems at 12 months. | **Sample**  *Recruitment:*   - 192 families - Recruited at pregnancy from postnatal maternity wards - Community sample - Fathers screened for postnatal depression during pregnancy (at recruitment). - All fathers with an EPDS above 10 were included, in addition to a random 1 in 4 sample of low scoring - Final study sample based on 168 families with data on exposure and outcome   *Father socio-demographics:*   - 88% completed A-level/ college or above education - 83% medium to high SES - Mean age, 35 years | **Tool extracted**  Global Rating Scales (GRS; Murray et al. 1996)  **Main tool domain:** quality of parent-infant interactions  *Interaction setting:*   - Location: home - Set-up: floor-mat and infant seat setting - Use of toys: with toys - Duration: 3-mins (x2) - Time point: 3-months   *Scoring format:*   - Likert scale: 5-points   *Rater characteristics:*  -Trained researcher | **Father-infant relationship quality**  Quality of father-infant interactions (3-months)  The quality of father, infant, and dyadic behaviours (GRS)  *Paternal interactive behaviours*  -Sensitivity  -Intrusiveness  -Remoteness  *Paternal affect*  -Depressive affect | **Offspring outcomes**  Child behaviour problems  *(12-months)*  **Measure:** Child Behavior CheckList (CBCL; Achenbach & Rescorla, 2000) | Longitudinal data analyses  *9-month follow-up from extracted exposure (3-months) to outcome variables (12- months)*  Results:  **Father-infant relationship quality and offspring outcomes**  *Paternal interactive behaviours*   - Infants of fathers who displayed more disengaged (non-intrusive) (OR = 5.33, CI = 1.39, 20.40) and remote behaviours (OR = 3.32, CI = .92, 12.05) during father-infant floor-mat interactions (3-months) were at increased risk of developing early externalising behavioural problems (12-months) - Non-significant association between paternal sensitivity during father-infant floor-mat interactions and infant behavioural problems - Non-significant association between paternal behaviours during father-infant seat interactions and infant behavioural problems   *Paternal affect*   - Non-significant association between paternal depressive affect during father-infant floor-mat and infant behavioural problems   Study limitations:   - Lack of generalizability (sample of fathers somewhat older with a higher level of education) - 24 father-infant dyads lost to follow-up at 12-months. | |
|  |  |  |  |  |  | |
| **22. Rossen et al. (2018)**  Australia  **Aim(s):** To examine the influence of mother–infant and partner–infant bonding, mental ill-health, and substance use at 8 weeks post birth on mother–infant and partner–infant emotional ability at 12 months post birth. | **Sample**  *Recruitment:*  - 191 mother-partner dyads   - Mother or partners were primary caregivers - Community sample - Recruited at pregnancy through antenatal clinics   *Father socio-demographics:*   - 72% higher education - 53% australian, 22% other English speaking country, 20% non-english speaking - 85% full-time work - Mean age, 35 years - 70% families high SES | **Tool extracted**  Emotional Availability Scales (EAS; Biringen et al. 2008)  **Main tool domain** Emotional availability in parent-child interactions  *Interaction setting:*   - Location: home - Set-up: free-play session - Use of toys: with or without own toys - Duration: 20-minutes - Time point: 12-months   *Scoring format:*   - Frequency counts   *Rater characteristics:*  -Trained researchers | **Paternal psychopathology**  *Depressive symptoms*  *(8-weeks)*  **Measure:** EPDS (Cox et al., 1987)  *Stress and anxiety* symptoms (*8-weeks)*  -Stress symptoms  -Anxiety symptoms  **Measure:** Depression and Anxiety Stress Scales (DASS-21; Lovibond & Lovibond, 1995) | **Father-infant relationship quality**  Emotional availability in partner-child interactions  *(12-months)*  The quality of emotional communication and interaction in partner–infant dyads (typically fathers) (EAS)  *Paternal interactive behaviours*  -Sensitivity  -Structuring  -Non-intrusiveness  -Hostility  *Infant interactive behaviours*  *-*Responsiveness  -Involvement  *Total father emotional availability (composite of parent and infant sub-domains) was examined in relation to psychopathology* | Longitdudinal data analyses  *10-month follow-up from extracted exposure (8-weeks) to outcome variables (12-months)*  Results  **Paternal psychopathology and father-infant relationship quality**   - Non-significant association between paternal depressive symptoms (8-weeks; EPDS) and fathers’ emotional availability during father-infant interactions (12-months) - Non-significant association between paternal stress (DASS sub-scale) and anxiety symptoms (DASS sub-scale) (8-weeks) and fathers’ emotional availability during father-infant interactions   Study limitations:   - Observational measurements prone to bias such as the “halo effect,” – i.e., coding all dimensions similarly - Lack of generalizability, sample mainly Anglo-Saxon Australians | |
|  |  |  |  |  |  | |
| **23. Sethna et al. (2012)**  United Kingdom  **Aim(s):**  To examine  the speech of depressed and non-depressed fathers during father–infant interactions at 3 months. | **Sample**  *Recruitment:*   - 38 father-infant dyads - Community sample - Recruited at birth from postnatal maternity wards - All fathers screened for depression at 7-weeks (EDPS), followed by the SCID at 3-months - Two matched groups: 19 depressed (EDPS > 10, positive SCID for MDD); 19 non-depressed fathers (EDPS < 10, no current/ past diagnosis)   *Father socio-demographics:*   - Majority had completed a diploma or above - All fathers were married - Majority of white ethnicity - Age range, 34 – 36 years | **Tool extracted**  Paternal Cognitive Attributional Mentalizing Scale (PCAMS; Sethna et al., 2012)  **Main tool domain:** paternal speech during father-infant interactions  *Interaction setting:*   - Location: home - Set-up: infant-seat setting - Use of toys: without toys - Duration: 3-mins - Time point: 3-months   *Scoring format:*   - Frequency counts of occurrence / proportion - Total time / proportion of time (secs)   *Rater characteristics:*  -Trained researcher | **Paternal psychopathology**  Clinical diagnosis of major depressive disorder  *(3-months)*  **Measure:** DSM-IV Axis 1 (SCID; First et al., 1997) | **Father-infant relationship quality**  Quality of paternal speech  *(3-months)*  Proportion of total paternal speech utterances during father-infant interactions (PCAMS)  -Attentional focus of speech  -Affective focus of speech  -Mentalizing comments | Cross-sectional data analyses  *Extracted exposure and outcome variables both measured at 3-months*  Results  **Paternal psychopathology and father-infant relationship quality**  *Paternal speech*   - Fathers in the clinically depressed group displayed higher proportion of paternal speech focused on the paternal experience (Z = -2.17, *p* = .01) and less on the infants’ (Z = -2.37, *p* = .008), and more infant-directed negativity (Z = -2.01, *p* = .002), during father-infant interactions, compared to fathers in the non-depressed group. - Non-significant difference in paternal mentalizing speech between paternal clinical diagnostic groups   Study limitations:   - Lack of generalizability (homogenous sample, SES) - Large number of tests conducted, possible risk of type I errors | |
|  |  |  |  |  |  | |
| **24. Sethna et al. (2015)**  United Kingdom  **Aim(s):**  To investigate the link between paternal major depressive  disorder and the quality of father–infant interactions at three-months postpartum, using an interview assessment of depression and an observed measure of parenting in two interactive settings. | **Sample**  *Recruitment:*   - 192 father-infant dyads - Community sample - Recruited at birth from postnatal maternity wards - All fathers screened for depression at 7-weeks (EDPS), followed by the SCID at 3-months - Two groups: 54 depressed (EDPS > 10, positive SCID for MDD); 99 non-depressed fathers (EDPS < 10, no current or past diagnosis) - Depression groups, sample for group analysis - Total sample (*n* = 192), included in analysis of continuous depressive scores   *Father socio-demographics:*   - 76% had an education level of dipoma or above - 191 fathers were married - 92% of white ethnicity - Mean age, 35 years   *(based on total sample)* | **Tool extracted**  Global Rating Scales (GRS; Murray et al. 1996)  **Main tool domain:** quality of parent-infant interactions  *Interaction setting:*   - Location: home - Set-up: infant car-seat and floor-mat interactions - Use of toys: without toys - Duration: 3-mins (x2) - Time point: 3-months   *Scoring format:*   - Likert scale: 5-points   *Rater characteristics:*  -Trained researcher | **Paternal psychopathology**  Clinical diagnosis of major depressive disorder  *(3-months)*  **Measure:** DSM-IV Axis 1 (SCID; First et al., 1997)  *Depressive symptoms*  *(7-weeks)*  **Measure:** EPDS (Cox et al., 1987)  **Maternal psychopathology**  *Depressive symptoms*  *(3-months)*  **Measure:** EPDS (Cox et al., 1987)  *Note:* maternal depressive symptoms were including as a main potential covariate variable – with bi-variate correlations conducted in relation to father-infant interactions | **Father-infant relationship quality**  Quality of father-infant interactions (3-months)  Paternal interactive behaviours examined during father-infant interactions (GRS)  *Paternal interactive behaviours*  -Sensitivity  -Intrusiveness  -Remoteness  *Paternal affect*  -Depressive affect | Mainly cross-sectional data analyses  *Up to 5-week follow-up from extracted exposure (7-weeks and 3-months) and outcome variables (3-months)*  Results  **Paternal psychopathology and father-infant relationship quality**  *Paternal interactive behaviours*   - Fathers in the clinically depressed group (3-months) displaying lower mean scores in intrusiveness, compared to fathers in the non-depressed group (*t* = -1.98, *p* = 0.05) *(marginal significance)* during father-infant interactions floor-mat interactions only (3-months) - Non-significant difference in paternal remoteness or sensitivity between clinical diagnostic groups - Increased depressive symptoms (EPDS) (7-weeks) were associated with less intrusiveness (higher scores) (*r* (176) = .15, *p* = .043) during father-infant floor-mat interactions only (3-months)   *Paternal affect*   - Increased depressive symptoms (EPDS) (7-weeks) were associated with increased displays of depressive affect (*r* (176) = .15, *p* = .053; *marginal*) during father-infant floor-mat interactions only - Non-significant difference in paternal interactive depressive affect between clinical diagnostic groups     **Maternal psychopathology and father-infant relationship quality**   - Non-significant associations between maternal depressive symptoms and paternal interactive behaviours or affect during father-infant floor-mat or infant seat interactions (3-months)   Study limitations:   - Lack of generalizability (homogenous sample, SES) - Magnitude of the differences between depressed and non-depressed fathers was not large | |
|  |  |  |  |  |  | |
| **25. Sethna et al. (2018)**  United Kingdom  **Aim(s):**  To examine links between diagnosed paternal depression in the postnatal period and playfulness in father-infant interactions. | **Sample**  *Recruitment:*   - 38 father-infant dyads - Community sample - Recruited at birth from postnatal maternity wards - All fathers screened for depression at 7-weeks (EDPS), followed by the SCID at 3-months - Two groups: 19 depressed, 19 non-depressed fathers   *Father socio-demographics:*   - Most had higher education - All were married - Majority of white ethnicity - Age range, 34 – 36 years | **Tool extracted**  Paternal-Physicality, Affect & Touch Scale (P-PATS; Sethna et al. 2018)  **Main tool domain:** playful behaviours during father-infant interactions  *Interaction setting:*   - Location: home - Set-up: free-play - Use of toys: without toys - Duration: 3-minutes - Time point: 3-months   *Scoring format:*   - Frequency and proportions (playful excitation and active engagement) - Time duration (secs) (quantity of touch) - Proportion of time   (quality of touch)   - Likert scale, 5-points (physicality in play)   *Rater characteristics:*  -Trained researcher | **Paternal psychopathology**  Clinical diagnosis of major depressive disorder  *(3-months)*  **Measure:** DSM-IV Axis 1 (SCID; First et al., 1997) | **Father-infant relationship quality**  Quantity and quality of paternal playful behaviours (P-PATS) (3-months)  *Paternal interactive behaviours*   - Physicality in play - Playful excitation - Active engagement - Tactile stimulation - including the overall time spent in touch (quantity), and time spent in gentle and vigorous touch – quality | Cross-sectional data analyses  *Extracted exposure and outcome variables both measured at 3-months*  Results  **Paternal psychopathology and father-infant relationship quality**  *Paternal interactive behaviours*   - Fathers in the clinically depressed group displayed lower episodes playful excitation (*p* = .005) and active engagement (*p* = .044), and spent less time engaged in gentle touch (*p* = .02), compared to fathers in the non-depressed group (3-months) - Non-significant differences in episodes of physicality in play, or the overall duration of touch (quantity) and time spent in vigorous touch (quality), between clinical diagnostic groups   Study limitations:   - Lack of generalizability (homogenous sample, SES) - No formal power analysis in advance of the study conducted | |
|  |  |  |  |  |  | |
| **26. Sethna et al. (2019)**  UK  **Aim(s):** To investigate the relationship between observed father–infant interactions,  specifically paternal sensitivity, and regional brain volumes in a community sample of 3-to-6-month-old infants | **Sample**  *Recruitment:*   - 28 father-infant dyads - Community sample   *Father socio-demographics:*   - Most held managerial or professional occupations - Majority of white ethnicity - Mean age, 37-years | **Tool extracted**  Global Rating Scales (GRS; Gunning & Murray, 2002)  **Main tool domain:** Quality of parent-infant interactions  *Interaction setting:*   - Location: home - Set-up: infant-seat setting - Use of toys: without toys - Duration: 5-minutes - Time point: 3-6 months   *Scoring format:*   - Likert scale: 5-points   *Rater characteristics:*  -Trained raters | **Father-infant relationship quality**  Quality of father-infant interactions (3-6 months)  Paternal interactive behaviours examined during father-infant interactions (GRS)  *Paternal interactive behaviours*  -Sensitivity | **Offspring outcomes**  Infant brain development (subcortical and midbrain)  *(3-6 months)*  **Measure:** Magnetic Resonance Imaging | Cross-sectional data analyses  *Extracted exposure and outcome variables both measured at 3-6 months*  Results  **Father-infant relationship quality and offspring outcomes**   - Increased levels of paternal sensitivity were associated with smaller infant cerebellar volumes (*β* = -.38, *p* = 0.048) - Non-significant association between paternal sensitivity and subcortical grey matter volumes   Study limitations:   - Relatively small sample size (as reported by the authors) - Lack of generalizability (homogenous sample, SES) - Cross-sectional data analyses - causality cannot be inferred | |
|  |  |  |  |  |  | |
| **27. Tambelli et al. (2015)**  Italy  **Aim(s):** To assess the quality of parental interactions  during feeding in families with mothers with early traumatic experiences. | **Sample**  *Recruitment:*  - 136 families   - Three groups: (A) mothers with past physical/sexual abuse, (B) mothers with past emotional abuse, (C) No previous trauma   - First-time parents  - Community sample   - Recruitment at pregnancy through gynaecologic services   *Note:* the extracted analyses focused on the total sample of fathers which examined associations between paternal psychopathology and father-infant interactions - not in relation to partners of mothers in the separate risk groups  *Father socio-demographics:*  -Mean age, 37 years | **Tool extracted**  Observation Scale for Mother-Infant Interactions during Feeding (SVIA; Chatoor et al., 1997)  **Main tool domain:** quality of parent-infant feeding interactions  *Interaction setting:*   - Location: home - Set-up: observed during a lunchtime period - Use of toys: n/a - Duration: 20-minutes - Time point: 3-months   *Scoring format:*   - Likert scale: 4-points   *Rater characteristics:*  -Trained psychologists | **Paternal psychopathology**  Psychological symptoms  *(3-months)*  *Symptom sub-domains:*  -Somatization  -Obsessive compulsive  -Interpersonal sensitivity  -Depression  -Anxiety  -Hostility  -Phobic anxiety  -Paranoid ideation  -Psychoticism.  **Measure:** SCL-90-R (Derogatis, 1994) | **Father-infant relationship quality**  Quality of father-infant feeding interactions  *(3-months)*  The degree to which interactive behaviours are present or absent during feeding periods (SVIA)  *Paternal interactive behaviours*  -Interactional conflict  *Paternal affect*  -Overall affective state  *Infant interactive behaviours*  -Food refusal behaviours  *Dyadic affect*  -Dyadic Affective state | Cross-sectional data analyses  *Extracted exposure and outcomes variables were both measured at 3-months*  Results  **Paternal psychopathology and father-infant relationship quality**  *Paternal interactive behaviours*   - Non-significant association between paternal psychological symptom sub-domains (3-months) and paternal interactive behaviours during father-infant feeding interactions (3-months)   *Infant interactive behaviours*   - Increased paternal anxiety symptoms were associated with increased scores in infant food refusal behaviours (*ß* = .32, *p* = .01) - Non-significant associations between the remaining psychological symptom domains and infant food refusal   *Dyadic affect*   - Non-significant association between paternal psychological symptom sub-domains and dyadic affective state   Study limitations   - Infant’s emotional/behavioural functioning not examined which may have weight on parental interaction styles - Lack of generalizability (homogenous sample, SES) | |
|  |  |  |  |  |  | |
| **28. Tamis-LeMonda et al., (2004)**  USA  **Aim(s):** To compare the engagements of low-income fathers and  mothers at play with their 2- and 3-year-olds, and to determine how fathers' engagements, in particular, relate to children's language and cognitive development | **Sample**  *Recruitment:*   - 111 fathers and their partners - Community sample - Biological resident fathers   *Paternal socio-demographics:*   - 60% European American, 22% African American, 15% Latino and 3% of other ethnic background - 36% completed college or graduate school - Age range, 19-51 years | **Tool extracted**  National Institute of Child Health and Human Development coding scales (NICHD; Early Child Care Research Network, 1999) – adapted version  **Main tool domain:** quality of parent-infant interactions  *Interaction setting:*   - Location: home - Set-up: semi-strucutred free-play session - Equipment: with toys - Duration: 10-minutes - Time point: 24-months   *Scoring format:*   - Likert scale: 7-points   *Rater characteristics:*  -Researcher | **Father-infant relationship quality**  Quality of father-infant interactions *(24-months)*  The degree to which parent responses are well timed and appropriate to the child's signals (NICHD)  *Paternal interactive behaviours*  -Sensitivity  -Positive regard  -Cognitive stimulation  - Intrusiveness  - Detachment  - Negative regard  *Individual sub-scales for parent behaviour as well as a compote score of fathers supportive behaviour (i.e., sensitivity, positive regard, and cognitive stimulation) were examined in relation to offspring outcomes.* | **Offspring outcomes**  Infant mental development  *(24, 36-months)*  **Measure:** The Mental scale of the BSID-II (Bayley,  1993)  Receptive vocabulary  *(36-months)*  **Measure:** PPVT-III (Dunn & Dunn, 1997) | Cross-sectional and longitudinal data analyses  *Up to 12-month follow-up from extracted exposure (24-months) and outcomes variables (24, 36-months)*  Results  **Father-infant relationship quality and offspring outcomes**  *Paternal interactive behaviours*   - Increased levels of paternal sensitivity and cognitive stimulation (24-months) were associated with higher scores in infant MDI scores at 24-months (*r* = .21, *r* = .28 (*p* < .01_ respectively) and at 36-months (*r* = .30, *r* = .30 (*p* < .01), respectively) and infant PPVT scores at 36-months (*r* = .26, *r* = .25 (*p* < .01), respectively) - Increased levels of paternal detachment were associated with lower infant MDI scores at 36-months (*r* = -.17, *p* < .05) - Increased levels of paternal intrusiveness were associated with lower infant MDI scores at 24- (*r* = -.15, *p* <.05) and 36-months (*r* = -.18, *p* < .05) - Increased scores in paternal supportive behaviours (composite scores) were associated with higher infant MDI scores at 24 and 36-months (*ß* = .14, *p* > .05; *ß* = .17, *p* < .01, respectively), and infant PVVT scores at 36-months (*ß* = .25, *p* < .01) - Non-significant association between paternal levels of detachment and infant MDI scores at 24-months and PPVT scores at 36-months - Non-significant association between paternal intrusiveness and infant PPVT scores at 36-months   *Paternal affect*   - Increased levels of paternal positive regard (24-months) were associated with higher scores in infant MDI scores at 24-months (*r* = .21, *p* < .01) and 36-months (*r* = .22, *p* < .01) and PPVT scores at 36-months (*r* = .25, *p <* .01) - Non-significant association between paternal negative regard and infant MDI scores at 24- and 36-months and PPVT scores at 36-months   Study limitations   - Lack of generalizability (homogenous sample, SES) - Observations assessed during free-play – potentially limiting the ability for negative parenting - Offspring outcome measures assessed using standardised assessments – potentially limiting their ability to capture the rich variation in outcomes | |
|  |  |  |  |  |  | |
| **29. Towe-Goodman et al., (2014)**  USA  **Aim(s):** To examine longitudinal links between fathers’ sensitive parenting in infancy and toddlerhood and children’s early executive functioning. | **Sample**  *Recruitment:*   - 620 mothers, fathers and their infants recruited from local hospitals at birth - Part of a larger longitudinal study, Family Life Project - Sample weighted towards low-income and African-American families   *Paternal socio-demographics:*   - Fathers mean education in years: 13.15 | **Tool extracted**  National Institute of Child Health and Human Development coding scales (NICHD; Early Child Care Research Network, 1999)  **Main tool domain:** Quality of parent-infant interactions  *Interaction setting:*   - Location: home - Set-up: semi-strct play - Equipment: with toys - Duration: 10-minutes - Time point: 7, 24-months   *Scoring format:*   - Likert scale: 5-points   *Rater characteristics:*  -Trained raters (until reliabity met at ICC > .80) | **Father-infant relationship quality**  Quality of father-infant interactions *(7, 24-months)*  The degree to which parent responses are well timed and appropriate to the child's signals (NICHD)  *Paternal interactive behaviours*  -Sensitivity  -Cognitive stimulation  - Intrusiveness  - Detachment  - Animation in interaction  *Paternal affect*  - Positive regard  *Note: individual subscales were composited to form an overall measure of paternal sensitive parenting* | **Offspring outcomes**  Child executive functioning  (*3-years*)  Child executive functioning as assessed using various tasks administered in a flip-book format (Willoughby et al., 2010) | Longitudinal data analyses  *Up to 2.5-year follow-up from extracted exposure (7- and 24-months) and outcomes variables (3-years)*  Results  **Father-infant relationship quality and offspring outcomes**   - Paternal sensitive parenting (7-months) was not a significant predictor of child executive functioning (3-years); *ns* - Increased paternal sensitive parenting (24-months) significantly predicted higher scores in child executive functioning (3-yerars)   (*ß* = 5.44, p = <.01)  Study limitations   - Sensitive parenting observed during one context, not multiple - Sample weighted towards families residing in poor rural counties – potentially lack of generalizability to more advantaged families | |
|  |  |  |  |  |  | |
| **30.Trautmann-Villalba et al., (2006)**  Germany  **Aim(s):**  To examine the extent to which fathers’ and infants’ interaction behavior are related to children’s externalizing behavior problems at age 8 and 11 years. | **Sample**  *Recruitment:*   - 362 infants born at 8 hospitals in Germany between 1986 and 1988 - Community sample - 91 fathers participated in the 3-month video assessment. - Final sample included 71 fathers who met inclusion criteria at 8-11 years   *Paternal socio-demographics:*   - Mean age range at childbirth, 31 years (fathers of children with low scoring externalizing), 30 years (fathers of children with high scoring externalizing) | **Tool extracted**  Categorical System for Micro-Analysis of the Early Mother–Child Interaction (Jorg et al. 1994)  **Main tool domain:** micro-analysis of parent and infant interactive behaviours  *Interaction setting:*   - Location: lab setting - Set-up: standardized nursing and play situation - Use of toys: n/a - Duration: 5-minutes - Time point: 3-months   *Scoring format:*   - Time duration (secs) - Frequency counts   *Rater characteristics:*  -Trained raters  *Note:* authors describe that the tool was adapted for use in father-infant interactions | **Father-infant relationship quality**  Father-infant interactions behaviours *(3-months)*  The frequency of paternal and infant interactive behaviours (Categorical System for Micro-Analysis of the Early Mother–Child Interaction)  *Paternal interactive behaviours*  -Sensitive fathering  -Non-responsiveness  *Paternal affect*  -Positive emotionality  *Infant affect*  -Positive emotionality  -Negative emotionality | **Offspring outcomes**  Child behaviour problems  *(8-11-years)*  **Measure:** Child Behavior Checklist (CBCL; Achenbach & Rescorla, 2000) | Longitudinal data analyses  *8-11 year follow-up from exposure (3-months) to outcome variables (8-11-years)*  Results  **Father-infant relationship quality and offspring outcomes**  *Paternal interactive behaviours*   - Children scoring high in externalizing behaviours (CBCL > 60) (8-11 years) were exposed to lower levels of sensitive (F (1,67) = 8.24, *p* = .005) (*d* = 4.2) and responsive behaviours (F (1,67) = 5.17, *p* = .026) (*d* = 3.2) during father-infant interactions (3-months), compared to children scoring low in externalising behaviours (CBCL scores < 60)   *Paternal affect*   - Non-significant difference in positive emotionality during father-infant interactions and child externalizing behaviour   *Infant affect*   - Children scoring high in externalizing behaviours (CBCL > 60) displayed increased levels of positive emotionality towards the father during father-infant interactions (3-months), compared to children scoring low in externalising behaviours (CBCL scores < 60) (F(1,67) = 4.71, *p* = 0.034) (*d* = 3.1) - Non-significant difference in infant negative emotionality during father-infant interactions and child externalizing behaviour   Study limitations   - Observations in a lab setting – restricted conditions might reflect natural or typical interaction behaviour of the participants. | |
|  |  |  |  |  |  | |
| **31. Volling et al., (2019)**  USA  **Aim(s):** To explore whether fathers and mothers engaged in a form of activation parenting (i.e., sensitivity, cognitive stimulation, and moderate intrusiveness) with their secondborn, 12-month-old infants during a 15-min challenging teaching task, and to determine if this type of interaction was more common among fathers. | **Sample**  *Recruitment:*   - 195 families (mothers, fathers and their infants) - Community sample - Recruitment during the mothers pregnancy   *Father socio-demographics:*   - Mean age, 32-years | **Tool extracted**  National Institute of Child Health and Human Development coding scales (NICHD; Early Child Care Research Network, 1999)  **Main tool domain:** Quality of parent-infant interactions  *Interaction setting:*   - Location: Lab - Set-up: structured play - Equipment: with toys - Duration: 15-minutes - Time point: 12-months   *Scoring format:*   - Likert scale: 7-points   *Rater characteristics:*  -Trained rater | **Father-infant relationship quality**  Quality of father-infant interactions *(12-months)*  The degree to which parent responses are well timed and appropriate to the child's signals (NICHD)  *Paternal interactive behaviours*  -Sensitivity  -Cognitive stimulation  - Intrusiveness  - Detachment  *Paternal affect*  - Positive regard | **Offspring outcomes**  Infant-attachment security  *(12-months)*  Infant attachment styles were classified during observations of infant–father strange situations  *Infant attachment styles*  -Secure attachment style  -Insecure attachment style  **Measure:** Strange Situation Procedure (SSP; Ainsworth et al., 1978) | Cross-sectional data analyses  *Extracted exposure and outcome variables measured at 12-months*  Results  **Father-infant relationship quality and offspring outcomes**   - Non-significant association between paternal interactive behaviours or paternal affect and infant attachment security   Study limitations   - Observations of behaviours may be limited to the context (i.e., challenging teaching context) - Cross-sectional data analyses - causality cannot be inferred - Lack of generalizability (homogenous sample, SES) | |
|  | | | | | | |
