# Supplementary material for: The Applicability and Performance of Tools Used to Assess the Father-Offspring Relationship in Relation to Parental Psychopathology and Offspring Outcomes
Source: Front Psychiatry. 2021 Jan 5;11:596857. doi: 10.3389/fpsyt.2020.596857 (PMC7814871; doi:10.3389/fpsyt.2020.596857)
Supplement: Supplementary file 5 [file Table_5.docx]

| **Supplementary Materials_Table 5**  Descriptive characteristics of studies utilising self-report tools to assess the father-offspring relationship – including relationship quality and father involvement | | | | | | |
| --- | --- | --- | --- | --- | --- | --- |
| **Publication / Country / Aim(s)** | **Sample details** | **Self-report tool** | **Type of exposure variable(s)** *(Paternal and /or maternal psychopathology,*  *Relationship quality, Father-involvement)* | **Type of outcome variable(s)**  *(Relationship quality, Father-involvement, Offspring outcomes)* | **Data analysis/ Results /Limitations** | |
|  | | | | | | |
| **Self-report tools (*n* = 18) extracted from *n* = 30 publications** | | | | | | |
|  | | | | | | |
|  |  |  |  |  |  | |
| **1. Barry et al. (2011)**  USA  **Aim(s):** To examine how perceived skill is  established and associated with paternal involvement in childcare, breastfeeding,  maternal gatekeeping, mothers’ work hours, fathers’ depressive symptoms,  and fathers’ beliefs about responding to a crying new-born. | **Sample**  *Recruitment:*  - 152 expectant couples   - First-time parents, dual earner couples   - Community sample   - Recruitment at pregnancy through prenatal classes   *Father socio-demographics*  -Mean age, 29 years  -90% were of white ethnicity  -78% were married  -53% had vocational or college education | **Tool extracted**  An unnamed tool (Barnett & Baruch, 1987)  **Main tool domain:** Parental engagement in routine childcare tasks  *Time point:* 1, 12-months  *Time to complete:* n/r  *Total items:* 15  *Scoring format:*  -Likert scale: 5-points | **Paternal psychopathology**  Depressive symptoms  *(1 and 12-months)*  **Measure:** Epidemiologic Studies Depression Scale (CES-D; Radloff, 1977) | **Father involvement**  Fathers involvement in child care activities (*1 and 12-months)*  Involvement in 15 childcare tasks (e.g., feeding, bathing, take child on outings – museum/park, take child to doctors etc.) - a measure of fathers’ involvement in proportion to maternal involvement (unnamed tool, Barnett & Baruch, 1987)  *Total scores of father involvement were examined in relation to paternal psychopathology* | Cross-sectional analyses extracted  *Extracted exposure and outcome variables both measured at 1 and 12-months*  Results  **Paternal psychopathology**  **and father involvement**     - Non-significant difference in paternal overall involvement in childcare activities between higher (CES-D > 16) *vs.* lower (CES-D < 16) depressive symptoms in fathers (1 and 12-months) - Non-significant association between depressive symptoms (total scores) and overall involvement in child care tasks     Study limitations   - No objective measures of paternal involvement – subject to rater bias - No absolute measure of fathers’ involvement – father involvement measured in proportion to maternal involvement | |
|  |  |  |  |  |  | |
| **2. Beesley et al., (2019)**  USA  **Aim(s):** To analyse the correlates of anxiety, depression, and the paternal-foetal attachment in expectant fathers. | **Sample**  *Recruitment:*  - 166 expectant fathers  - Community sample  - Recruited through social  media and websites  *Father socio-demographics:*   - 79% had an undergraduate degree or above - Mean age, 31-years - 92% in full employment - 92% first-time fathers - 83% were married - 87% were of white ethnicity | **Tool extracted**  Paternal Antenatal Attachment Scale (PAAS; Condon et al., 1993)  **Main tool domain:** Father-foetal attachment  *Time point:* antenatally  *Time to complete:* n/r  *Total items:* 16  *Scoring format:*  -Likert scale: 5-points | **Paternal psychopathology**  Depressive symptoms  *(measured antenatally)*  **Measure:** EPDS (Cox et al., 1987)  Anxiety symptoms *(antenatally)*  **Measure:** The Generalized Anxiety Disorder (GAD-7; (Spitzer, Kroenke, Williams, & Lowe, 2006) | **Relationship quality**  Fathers attachment to the foetus  *(measured antenatally)*  The feelings, attitudes, and behaviours towards the foetus.  (PAAS)  *Paternal behaviours*  -Quality of attachment  -Intensity of preoccupation  *Total scores of paternal behaviours related to fathers attachment to the foetus were examined in relation to paternal psychopathology* | Cross-sectional analyses extracted  *Extracted exposure and outcome variables both measured antenatally*  Results  **Paternal psychopathology and fathers attachment to the foetus**     - Non-significant association between paternal antenatal depressive or anxiety symptoms and total scores in fathers attachment to the foetus   Study limitations   - Lack of generalizability (homogenous sample, SES) - No objective measures of parenting– subject to rater bias - Use of categories with cut-off scores for anxiety and depression only indicating a likelihood | |
|  |  |  |  |  |  | |
| **3. Brandão et al. (2019)**  Portugal  **Aim(s):** To explore the intrapersonal and interpersonal effects of mothers' and fathers' anxiety and depressive symptoms on their own and their partners' antenatal attachment to the foetus. | **Sample**  *Recruitment:*  - 320 expectant couples   - Population sample - Recruitment during pregnancy at private and public hospital clinics   *Father socio-demographics:*   - 37% had a minimum 12-years of education - Mean age, 33-years | **Tool extracted**  Paternal Antenatal Attachment Scale (PAAS; Condon et al., 1993)  **Main tool domain:** father-foetus attachment  *Time point:* antenatally  *Time to complete:* n/r  *Total items:* 16  *Scoring format:*  -Likert scale: 5-points | **Paternal psychopathology**  Anxiety and depressive symptoms  *(measured antenatally)*  **Measure:** Hospital Anxiety and Depression Scale (HADS; Zigmond & Snaith, 1983)  **Maternal psychopathology**  Anxiety and depressive symptoms *(antenatally)*  **Measure:** Hospital Anxiety and Depression Scale (HADS; Zigmond & Snaith, 1983) | **Relationship quality**  Fathers attachment to the foetus  *(measured antenatally)*  The feelings, attitudes, and behaviours towards the foetus.  (PAAS)  *Paternal behaviours*  -Quality of attachment  -Intensity of preoccupation  *Total scores of paternal behaviours related to fathers attachment to the foetus were examined in relation to paternal psychopathology* | Cross-sectional analyses extracted  *Extracted exposure and outcome variables both measured antenatally*  Results  **Paternal psychopathology and fathers attachment to the foetus**   - Increased paternal antenatal depressive symptoms were associated with lower total scores in fathers attachment to the foetus (*ß* = -.38, *p* < .001) - Increased paternal antenatal anxiety symptoms were associated with higher total scores in fathers attachment to the foetus (*ß* = .16, *p* < .05)   **Maternal psychopathology and fathers attachment to the foetus**   - Increased maternal antenatal depressive symptoms were associated with higher paternal reported total scores in his attachment to the foetus - Increased maternal antenatal anxiety symptoms were associated with lower paternal reported total scores in his attachment to the foetus   Study limitations   - Lack of generalizability (homogenous sample with regards SES) - No objective measures of parenting – self-reports subject to rater bias - Cross-sectional study design – limiting inferences of temporality | |
|  |  |  |  |  |  | |
| **4. Bronte-Tinkew et al., (2007)**  USA  **Aim(s):** To examine the sociodemographic correlates of symptoms of depression and  how depression is associated with father involvement | **Sample**  *Recruitment:*   - 2137 resident fathers and their partners   - Community sample   - Recruitment through a larger longitudinal cohort study, Fragile Families and Child Wellbeing Study   *Father socio-demographics:*   - Majority of fathers aged between 17-29 years - 61.3% completed high school education or above - 67.7% of Non-Hispanic Black ethnicity | **Tool extracted**  Home Observation for Measurement of the  Environment  (HOME-SF) -Short Form version (Baker & Mott, 1992)  **Main tool domain:** Fathers engagement in child care related activities  *Time point:* 12-months  *Time to complete:* n/r  *Total items:* 8  *Scoring format:*   - Likert scale: 7-points | **Paternal psychopathology**  Depressive symptoms  *(12-months)*  **Measure:** Composite International Diagnostic Interview–Short  Form (CIDI-SF; Kessler, Andrews, Mroczek, Utsun, & Wittchen,  1998)  Substance use/dependence  *(12-months)*  **Measure:** fathers self-report (fathers indicated whether they had 5 or more drinks in any one day in the past month)  **Maternal psychopathology**  Depressive symptoms  *(12-months)*  **Measure:** CIDI-SF | **Father involvement**  Father engagement in child care related activities  *(12-months)*  Fathers indicated the extent to which they engage in a range of engagement activities (HOME-SF) | Cross-sectional analyses extracted  *Extracted exposure and outcome variables measured at 12-months*  Results  **Paternal psychopathology and father involvement**   - Presence of paternal depression (symptoms scoring above the cut-off) were associated with a lower level of engagement in child care related activities (*β* = -1.10, *p* < 0.05)   **Maternal psychopathology and father involvement**   - Presence of maternal depression (symptoms scoring above the cut-off) were associated with a higher level of engagement in child care related activities (*β* = 0.33, *p* < 1.0; trend significance)   Study limitations   - Response rate was modest – potential bias across non-respondents - Cross-sectional study design – limiting inferences of temporality - Psychopathology assessed via self-report – no objective measure | |
|  |  |  |  |  |  | |
| **5. Brown, Mangelsdorf & Neff (2012)**  USA  **Aim(s):** To examine  concurrent and longitudinal associations among father involvement, paternal sensitivity, and father-child  attachment security at 13 months and 3 years of age | **Sample**  *Recruitment:*   - 115 father-infant dyads (103 dyads at 13-months, 71 dyads at 3-years).   - Community sample   - Recruitment through local community centres, retail outlets and mailing lists   *Father socio-demographics:*   - Mostly European American - 82% had higher education | **Tool extracted**  Responsibility Scale (PRS; McBride & Mills, 1993) (adapted version)  **Main tool domain:** parental responsibility in in child care tasks  *Time point:* 3-months  *Time to complete:* n/r  *Total items:* 14  *Scoring format:*  -Rating scale: *n/r* | **Father involvement**  Responsibility in child care  *(13-months)*  Fathers indicated who has primary responsibility (mother or father) for a range of childcare tasks (PRS) | **Offspring outcomes**  Infant-attachment security  *(13-months)*  Infant attachment styles were classified during observations of infant–father strange situations  *Infant attachment styles*  -Secure attachment style  -Insecure attachment style  **Measure:** Strange Situation Procedure (SSP; Ainsworth et al., 1978) | Cross-sectional analyses extracted  *Extracted exposure and outcome variables measured at 13-months*  Results  **Father involvement and offspring outcomes**   - Non-significant difference in paternal responsibility between securely and insecurely attached infants   Study limitations   - Lack of generalizability (homogenous sample with regards SES) - Cross-sectional data analyses - causality cannot be inferred - Father involvement was limited to the dimension of parenting responsibility | |
|  |  |  |  |  |  | |
| **6. Buist et al. (2003)**  Australia  **Aim(s):** To assess what anxieties men expressed with the transition to parenthood and  what personal factors were associated with this anxiety, and to examine which of factors affect  parental competence and attachment to their infant | **Sample**  *Recruitment:*   - 225 first-time fathers with their pregnant partners   - Community sample   - Recruitment during pregnancy at hospitals   *Father socio-demographics:*   - Mean age, 31-years   -76% were married   - 79% had skilled or semi-professional occupations | **Tool extracted**  Paternal Postnatal Attachment Questionnaire (PPAQ)  *Note*, the authors describe use of an updated unpublished version of the PAQ for fathers – the PPAQ. This scale has since been published (Condon et al., 2008), although sub-scales differ between unpublished and published versions of the PPAQ. Initial source of the PAQ: Condon & Corkindale, 1998)  **Main tool domain:** father-infant attachment  *Time point:* 1, 4-months  *Time to complete:* n/r  *Total items:* 19  *Scoring format:*  -Likert scale: 5-points | **Paternal psychopathology**  Depressive symptoms  *(1 and 4-months)*  **Measure:** EPDS (Cox et al., 1987) | **Relationship quality**  Fathers attachment to the infant  *(1 and 4-months)*  The emotional bond or tie of affection experienced by  the parent towards the infant (PPAQ)  *Paternal behaviours*  -Quality of interaction  -Pleasure in interaction  -Absence of hostility | Cross-sectional data analyses extracted  *Up to 3-month follow-up from extracted exposure (1 and 4-months) and outcome variables (1 and 4-months): cross-sectional data extracted at each time-point*    Results  **Paternal psychopathology and fathers attachment to the infant**   - Fathers in the group reporting increased depressive symptoms (EPDS > 10) showed higher scores in hostility towards the infant (*p* < .05), and lower scores in quality of interaction (*p* < .05) at 1-month (*F* = 4.8, *p* < 0.05) and 4-months (*F* = 4.5, *p* < 0.05), compared fathers in the group reporting lower depressive symptoms (EPDS < 10). - Non-significant difference in paternal reported scores in pleasure in interaction sub-scale between paternal symptom level groups (1 and 4-months)   Study limitations   - Lack of generalizability (homogenous sample, SES) - No objective measures of parenting, self-report measures subject to rater bias | |
|  |  |  |  |  |  | |
| **7. Condon et al. (2008)**  Australia  **Aim(s):**  To demonstrate the feasibility of utilising a self-report approach for measuring father-infant attachment in both research and clinical settings | **Sample**  *Recruitment:*  - 241 first-time fathers  - Community sample   - Recruitment during partners pregnancy from antenatal clinics   *Father socio-demographics:*  - 69% had either professional, semi-professional or skilled professions  - 85% were in employment  - 76% were married | **Tool extracted**  Paternal postnatal attachment Questionnaire (PPAQ; Condon et al., 2008)  **Main tool domain:** father-infant attachment  *Time point:* 6, 12-months  *Time to complete:* n/r  *Total items:* 19  *Scoring format:*  -Likert scale: 5-points | **Paternal psychopathology**  Depressive symptoms  *(6 and 12-months)*  **Measure:** EPDS (Cox et al., 1987) | **Relationship quality**  Fathers attachment to the infant  *(6 and 12-months)*  The emotional bond or tie of affection experienced by  the parent towards the infant  (PPAQ)  *Paternal behaviours*  -Patience and tolerance  -Pleasure in interaction  -Affection and pride  *Total scores of paternal behaviours related to fathers attachment to the infant were examined in relation to psychopathology and offspring outcomes*  **Offspring outcomes**  Infant difficult temperament  *(6 and 12-months)*  Fathers reported on the degree of difficult temperament of his infant.  **Measure:** Infant Characteristic Questionnaire (ICQ; Bates et al., 1979) | Cross-sectional analyses extracted  *Up to 6-month follow-up from extracted exposure (6 and 12-months) and outcome variables (6 and 12-months): cross-sectional analyses extracted at each time-point*  Results  **Paternal psychopathology and fathers attachment to the infant**   - Increased paternal depressive symptoms were associated with lower total scores in fathers attachment to the infant at 6-months (*r* = -.24, *p* < .001) and 12-months (*r* = -.27, *p* < .001)   **Fathers attachment with the infant and offspring outcomes**   - Increased total scores in fathers attachment to the infant were associated with lower scores in infant difficult temperament at 6-months (*r* = -.37, *p* < .001)   Study limitations:   - Lack of generalizability (homogenous sample, SES) - No objective measures of parenting - 25 fathers lost to follow-up | |
|  |  |  |  |  |  | |
| **8. Dayton et al., (2019)**  USA  **Aim(s):** To examine parental risk and resilience factors on the prenatal parental-fetal bond in a sample of expectant mothers and fathers who reported high levels of exposure to contextual adversity | **Sample**  *Recruitment:*   - 51 expectant couples - Recruited from Detroit, Michigan and considered at risk for the development of harsh parenting due to exposure to environmental   *Father socio-demographics:*  - 60% of African American ethnicity  - Majority aged between 18-25-years  - 60% full-time employed  - 91% in a relationship | **Tool extracted**  Paternal Antenatal Attachment Scale (PAAS; Condon et al., 1993)  **Main tool domain:** father-foetal attachment  *Time point:* antenatally  *Time to complete:* n/r  *Total items:* 16  *Scoring format:*  -Likert scale: 5-points | **Paternal psychopathology**  Depressive symptoms  *(measured antenatally)*  **Measure:** EPDS  Anxiety symptoms  *(measured antenatally)*  **Measure:** STAI  PTSD symptoms  *(measured antenatally)*  **Measure:** Post-traumatic  Symptom Disorder Checklist-Civilian (PCL-C; Weathers  et al. 1993)  *Note*, an overall composite score (psychiatric distress) of depression, anxiety and PTSD symptoms examined in relation to fathers attachment to the foetus | **Relationship quality**  Fathers attachment to the foetus  *(measured antenatally)*  Fathers feelings, attitudes, and behaviours towards the foetus  (PAAS)  *Paternal behaviours*  -Quality of attachment  -Time in attachment | Cross-sectional analyses extracted  *Extracted exposure and outcome variables both assessed antenatally*  Results  **Paternal psychopathology and fathers attachment to the foetus**   - Non-significant association between paternal psychiatric distress and paternal quality of attachment or time in attachment   Study limitations:   - Relatively small sample size (as reported by the authors) - Cross-sectional data analyses - limiting inferences on causation | |
|  |  |  |  |  |  | |
| **9. de Cock et al., (2017)**  Netherlands  **Aim(s):** To examine the associations between parental bonding  (i.e., the affective tie from parent to child), parenting  stress, and child executive functioning. | **Sample**  *Recruitment:*   - 261 fathers and partners - Community sample recruited at pregnancy   *Father socio-demographics:*  - 85% were Dutch  - 68% 9+ years education  - Mean age, 34 years | **Tool(s) extracted**  Paternal Antenatal Attachment Scale (PAAS; Condon et al., 1993)  **Main tool domain:** father-foetal attachment  *Time point:* antenatally  *Time to complete:* n/r  *Total items:* 16  *Scoring format:*  -Likert scale: 5-points  Paternal postnatal attachment Questionnaire (PPAQ; Condon et al., 2008)  **Main tool domain:** father-infant attachment  *Time point:* 6, 24-months  *Time to complete:* n/r  *Total items:* 19  *Scoring format:*  -Likert scale: 5-points | **Relationship quality**  Fathers attachment to the foetus  *(measured antenatally)*  Fathers feelings, attitudes, and behaviours towards the foetus  (PAAS)  *Paternal behaviours*  -Quality of attachment  -Time in attachment  Fathers attachment to the infant  *(6 and 24-months)*  The emotional bond or tie of affection experienced by  the parent towards the infant (PPAQ)  *Paternal behaviours*  -Patience and tolerance  -Pleasure in interaction  -Affection and pride  *Total scores of paternal behaviours related to fathers attachment relationship towards the foetus / infant were examined in relation to offspring outcomes* | **Offspring outcomes**  Child executive functioning problems *(24-months)*  Fathers reported on the levels of executive functioning problems of their child  **Tool:** Behavior Rating Inventory of Executive Function-Preschool Version (BRIEF-P; Gioia et al. 2003) | Cross-sectional and longitudinal analyses extracted  *Up to 24-month follow-up from extracted exposure (antenatally, 6 and 24-months) and outcome variables (24-months)*  Results  **Fathers attachment to the foetus and offspring outcomes**   - Non-significant association between fathers attachment to the foetus and child executive functioning problems (24-months)   **Fathers attachment to the infant and offspring outcomes**   - Non-significant association between fathers attachment to the infant (6 and 24-months) and child executive functioning problems (24-months)   Study limitations:   - Utilised self-report measures of parenting and offspring outcomes –risk of bias / social desirability | |
|  |  |  |  |  |  | |
| **10. Edhborg et al. (2005)**  Sweden  **Aim(s):**  To investigate associations between blues, bonding, perception of the child’s temperament and depressive symptoms two  months postpartum in both parents. | **Sample**  *Recruitment:*   - 106 couples - Community sample recruited from maternity clinics at birth   *Father socio-demographics:*  - 50% completed college / university education  - 57% were married  - Mean age, 34 years  - 52% first-time fathers | **Tool extracted**  Postpartum Bonding Questionnaire (PBQ; Brockington et al., 2001)  **Main tool domain:**  father-infant bonding  *Time point:* 1, 8-weeks  *Time to complete:* n/r  *Total items:* 25  *Scoring format:*   - Likert scale: 5-points | **Paternal psychopathology**  Depressive symptoms  *(8-weeks)*  **Measure:** EPDS  **Maternal psychopathology**  Depressive symptoms  *(8-weeks)*  **Measure:** EPDS (Cox et al., 1987) | **Relationship quality**  Father-infant bonding difficulties *(8-weeks)*  The level of bonding difficulties between the parent and infant *(PBQ)*  *Paternal behaviours*  -Impaired bonding  -Rejection and anger  *Total scores of paternal behaviours related to fathers bonding difficulties with their infant were examined in relation to parental psychopathology and offspring outcomes*  **Offspring outcomes**  Infant difficult temperament  *(8-weeks)*  Fathers reported on the degree of difficult temperament of his infant.  **Tool:** Infant Characteristic Questionnaire (ICQ; Bates et al., 1979) | Cross-sectional analyses extracted  *Extracted exposure and outcome variables both measured 8-weeks*  Results  **Paternal psychopathology and father-infant bonding**     - Increased paternal depressive symptoms were associated with increased total scores in fathers perceived bonding difficulties with their infant (*β* = 0.82, *p* < 0.001)   **Maternal psychopathology and father-infant bonding**   - Non-significant association between maternal depressive symptoms and total scores in paternal reported bonding difficulties with their infant   **Father-infant relationship quality and offspring outcomes**   - Increased total scores in fathers perceived bonding difficulties with their infant were associated with higher scores in dull (*β* = 2.49, *p* = 0.002)   and unpredictable infant temperament (*β* = 1.81, *p* = 0.006)   - Non-significant association between total scores in paternal reported bonding difficulties with their infant and fussy-difficult infant temperament   Study limitations   - No objective measures of parenting reported - History of depression assessed via-self report | |
|  |  |  |  |  |  | |
| **11. Feldstein et al., (2004)**  USA  **Aim(s):** To investigate the relationship between self-reported father-infant attachment and infant attachment security at 12-months | **Sample**  *Recruitment:*   - 38 fathers, 59 mothers and their infant - (extracted analyses based on 38 fathers)   - Community sample   - Recruited from maternity centres, telephone calls, community papers   *Father socio-demographics*  - All were middle-class SES  - All were married  -Mean age, 35 years | **Tool extracted**  Paternal postnatal attachment Scale (PPAQ)  *Note*, the authors describe use of an updated unpublished version of the PAQ for fathers – the PPAQ. This scale has since been published (Condon et al., 2008), although sub-scales differ between unpublished and published versions of the PPAQ. Initial source of the PAQ: Condon & Corkindale, 1998)  **Main tool domain:** father-infant attachment  *Time point:* 12-months  *Time to complete:* n/r  *Total items:* 19  *Scoring format:*   - Likert scale: 5-points | **Relationship quality**  Fathers attachment to the infant  *(12-months)*  The emotional bond or tie of affection experienced by  the parent towards the infant (PPAQ)  *Paternal behaviours*  -Quality of interaction  -Pleasure in interaction  -Absence of hostility  *Total scores and sub-scales of paternal behaviours related to fathers attachment to their infant were examined in relation to offspring outcomes* | **Offspring outcomes**  Infant attachment security  *(12-months)*  Parents perception of infant behaviours which are characteristics of the infant related to attachment security (AQS) | Cross-sectional analyses extracted  *Exposure and outcomes both measured at 12-months*  Results  **Fathers attachment to the infant and offspring outcomes**   - Increased total scores in fathers attachment to their infant were associated with more secure infant attachment behaviours (*F* (1,26) = .67, *p* = .016, *r*_sp_ = 0.44) - Increased scores in fathers pleasure in interaction were associated with more secure infant attachment behaviours (*F* (1,35) = 6.77 (*p =* 0.014, *r*_sp_ = 0.40) - Non-significant association between neither fathers quality of interaction, nor and absence of hostility, and infant attachment behaviours   Study limitations:   - Lack of generalizability (homogenous sample, SES) - No objective measures of paternal parenting | |
|  |  |  |  |  |  |  |
| **12. Ferketich & Mercer (1995)**  USA  **Aim(s):**  To examine paternal-infant attachment of experienced and inexperienced fathers during early infancy | **Sample**  *Recruitment:*  - 172 fathers   - Two groups: 93 inexperienced, 79 experienced fathers (i.e., one or more child) - Community sample - Recruited during partners pregnancy   *Father socio-demographics*  - ~60% were professionals  - ~60% of white ethnicity  - ~90% were married   - Mean age 30 – 34 years | **Tool extracted**  How I feel about my baby now (FAB; Leifers, 1977).  **Main tool domain:** father-infant attachment  *Time point:* birth, 1, 4 and  8-months  *Time to complete:* n/r  *Total items:* 10  *Scoring format:*   - Likert scale: 4-points | **Paternal psychopathology**  Depressive symptoms  *(birth, 1, 4, 8-months)*  **Measure:** CES-D (Radloff, 1977)  Anxiety symptoms  *(birth, 1, 4, 8-months)*  **Measure:** State Anxiety Scale (Spielberger et al., 1983) | **Relationship quality**  Fathers attachment to the infant  *(birth, 1, 4, 8-months)*  Positive and negative statements reflecting parents present feelings towards infant (FAB) | Cross-sectional data analyses  *Exposure and outcome variables both measured at birth, 1, 4 and 8-months: cross-sectional analyses extracted at each time-point*  Results  **Paternal psychopathology and fathers attachment to the infant**   - *First-time fathers:*   Increased paternal depressive symptoms were associated with lower scores in fathers attachment to the infant at 1-month (*ß* = -.26, *p* < .001) and 4-months (*ß* = -.29, *p* = .013)   - Non-significant association between depressive symptoms and fathers attachment to the infant   (birth and 8-months)   - Non-significant association between anxiety symptoms and fathers attachment to the infant   (birth, 1, 4 and 8-months)  *Multiparous fathers:*   - Increased depressive symptoms were associated with lower scores in fathers attachment to the infant at birth (*ß* = -.28, *p* < .001) and 8-months (*ß* = -.47, *p* = .001) - Non-significant association between depressive symptoms and fathers attachment to the infant attachment (1 and 4-months) - Increased anxiety symptoms were associated with lower scores in fathers attachment to the infant at 1-month (*ß* = -.28, *p* < .001) and 4-months (*ß* = -.43, *p* = .002) - Non-significant association between anxiety symptoms and fathers attachment to the infant (birth and 8-months)   Study limitations:   - Lack of generalizability (homogenous sample, SES) - No objective measures of paternal parenting | |
|  | | | | | | |
| **13. Fijałkowska &   Bielawska-  Batorowicz (2019)**  Poland  **Aim(s):** To identify factors associated with parental pre- and postnatal attachment to a child | **Sample**  *Recruitment*  - 35 expectant couples  *Father socio-demographics:*    - 37% university educated  - Mean age, 29 years | **Tool extracted**  Paternal Antenatal Attachment Scale (PAAS; Condon et al., 1993)  **Main tool domain:** father-foetal attachment  *Time point:* antenatally  *Time to complete:* n/r  *Total items:* 16  *Scoring format:*  -Likert scale: 5-points  Paternal postnatal attachment Questionnaire (PPAQ; Condon et al., 2008)  **Main tool domain:** father-infant attachment  *Time point:* 2-8 weeks  *Time to complete:* n/r  *Total items:* 19  *Scoring format:*  -Likert scale: 5-points | **Paternal psychopathology**  Depressive symptoms  *(antenatally and at 2-8 weeks)*  **Measure:** EPDS (Cox et al., 1987) | **Relationship quality**  Fathers attachment to the foetus  *(measured antenatally)*  Fathers feelings, attitudes, and behaviours towards the foetus  (PAAS)  *Paternal behaviours*  -Quality of attachment  -Intensity of preoccupation  Fathers attachment to the infant  *(2-8 weeks)*  The emotional bond or tie of affection experienced by  the parent towards the infant (PPAQ)  *Paternal behaviours*  -Patience and tolerance  -Pleasure in interaction  -Affection and pride  *Total scores of paternal behaviours related to fathers attachment to the foetus / infant were examined in relation to psychopathology* | Cross-sectional data analyses  *Up to 4-month follow-up from extracted exposure and outcome variables (third trimester and at 2-8 weeks): cross-sectional data points extracted at each time-point*  Results  **Paternal psychopathology and father attachment to the foetus and the infant**   - Increased paternal antenatal depressive symptoms were associated with lower father-foetus attachment (*r* = -.36, *p* = <.05) - Increased paternal postnatal depressive symptoms were associated with lower father-infant attachment (2-8 weeks)   (*r* = -.45, *p* = <.01)  Study limitations:   - Relatively small sample size - Sample collected in one area of the country - Postnatal questionnaires collected at 2-8 weeks, not one time-point | |
|  |  |  |  |  |  | |
| **14. Fuertes et al., (2016)**  Portugal  **Aim(s):** To evaluate maternal and paternal contributions to infant–mother and  infant–father attachment at 12 and 18 months in an understudied  Portuguese sample. | **Sample**  *Recruitment:*   - 82 parents and their infant - Recruited at the Santo Espírito Hospital, Azores, Portugal.     *Father socio-demographics:*   - 16% had completed higher college education - All fathers had full-time occupations   -Majority were married | **Tool extracted**  Responsibility Scale (PRS; McBride & Mills, 1993) (Portuguese version; Lima, 2005)  **Main tool domain:** parental responsibility in in child care tasks  *Time point:* 12, 18-months  *Time to complete:* n/r  *Total items:* 14  *Scoring format:*  -Rating scale: 5-points | **Father involvement**  Responsibility in child care  *(12, 18-months)*  Fathers indicated who has primary responsibility (mother or father) for a range of childcare tasks (PRS) – these were further categorised in the current study into the following three involvement areas:   - Playing with the infant - Primary care - Health care | **Offspring outcomes**  Infant-attachment security  *(12, 18-months)*  Infant attachment styles were classified during observations of infant–father strange situations  *Infant attachment styles*  -Secure attachment style  -Insecure attachment style  **Measure:** Strange Situation Procedure (SSP; Ainsworth et al., 1978) – adapted version | Cross-sectional analyses extracted  *Exposure and outcome variables assessed at 12 and 18-months: cross-secitonal and longitudinal data extracted at each time-point*  Results  **Father involvement and offspring outcomes**   - Fathers of securely attached infants displayed increased levels of involvement in play at 12-months (*p* < .01) and 18-months (*p* < .001), compared to fathers of insecurely attached infants - Paternal involvement in play predicted infant secure attachment towards the father at 12-months (*β =* 8.74, CI = 0.000 – 0.079) and 18-months *β =* 17.03, CI = 0.000 – 0.001) (logistic regression models) - Fathers of securely attached infants displayed increased levels of involvement in primary care at 18-months only (*p* < .001), compared to fathers of insecurely attached infants - Paternal involvement in primary care predicted infant secure attachment towards the father at 18-months (*β =* 5.96, CI = 0.062 – 2.13) (logistic regression model) - Non-significant association between fathers involvement in health care and infant attachment security at 12 or 18-months   Study limitations:   - Findings may not be generalizable across all populations - Cross-sectional data analyses limiting inferences on causation | |
|  | | | | | | |
| **15. Giallo et al. (2015)**  Australia  **Aims(s):** To explore the longitudinal relationships between fathers’ psychological distress and parenting warmth, hostility, and consistency across early childhood. | **Sample**  *Recruitment:*  - 2662 fathers   - Primary/secondary caregiver - Biological fathers - Community sample - Families with an infant aged 3-12 months recruited based on a random selection of postcodes   *Father socio-demographics:*    - 78% were born in Australia  - 75% had a med-high SES  - 91% had full-time work  - Mean age, 34 years | **Tool extracted**  Child Rearing Questionnaire (CRQ; Paterson & Sanson (1999)  **Main tool domain:** parent behaviour towards the child  *Time point:* *3-12 months, 2-3, 4-5, 6-7 and 8-9-years*  *Time to complete:* n/r  *Total items:* 21  *Scoring format:*   - Likert scale: 5-points | **Paternal psychopathology**  Symptoms of psychological distress *(3-12 months)*  **Measure:** Kiessler-6  (K6; Kessler et al., 2003) | **Relationship quality**  Paternal warmth *(3-12 months)*  Parents indicates how often they express warmth and affection towards the child (CRQ)  *Paternal behaviours*  -Paternal warmth | Cross-sectional analyses extracted  *Extracted exposure and outcome variables both measured at 3-12 months*  Results  **Paternal psychopathology and father-infant relationship quality**   - Non-significant association between paternal symptoms of psychological distress and paternal warmth (3-12 months)   Study limitations:   - No objective measures of paternal parenting - Self-reports potentially influenced by negative perceptions about their own parenting | |
|  |  |  |  |  |  | |
| **16. Goodman et al. (2014)**  USA  **Aims(s):** To examine concurrent and prospective associations between maternal depression and father involvement and evaluate support for the spillover model – i.e., higher depressive symptom levels associated with lower father involvement, and the compensatory buffering model i.e., higher depressive symptom levels associated with higher father involvement | **Sample**  *Recruitment:*   - 129 families with consenting fathers - Women recruited at pregnancy through referrals of local obstetrical and mental health practitioners - Potential participants completed checklist and telephone interview - At enrolment, women were included if they fulfilled diagnostic criteria for lifetime history of depression or anxiety. - Their partners were invited to participate (50% response) - Final sample included 92 fathers participated with data 3-months, 85 at 6-months, and 74 at 12-months   *Father socio-demographics:*   - Completed an average of 16 years education   - 94% were married   - 90% European America, 9% African American   -Mean age, 36 years | **Tool extracted**  Unnamed (Goodman et al. 2014)  Tool devised by the authors by combining two published tools: The Parental Responsibility Scale (PRS; McBride & Mills, 1993) and the Child Care Activity Questionnaire (Montague & Walker-Andrews, 2002)  **Main tool domain:** parental responsibility in in childcare tasks  *Time point:*3, 6, 12-months  *Time to complete:* n/r  *Total items:* 24  *Scoring format:*  -Rating scale: 5-points  *Rater characteristics:*   - Trained researcher | **Maternal psychopathology**  Depressive symptoms  *(birth, 3, 6 and 12-months)*  **Measure:** BDI (Beck, 1978)  Lifetime history of depression or anxiety disorder *(measured at enrolment)*  **Measure:** DSM-IV-R (SCID; First et al., 2002)  **Paternal psychopathology**  Lifetime history of depression or anxiety disorder  *(measured at enrolment)*  **Measure:** DSM-IV-R (SCID; First et al., 2002)  *Note:* paternal and maternal history of depression or anxiety were not main exposure variables, but examined included as potential covariates examined in relation to paternal parenting measures | **Father involvement**  Responsibility in child care  *(3, 6 and 12-months)*  Fathers indicate who has primary responsibility (mother or father) for a range of childcare tasks (unnamed, Goodman et al., 2014) | Cross-sectional and longitudinal analyses extracted  *Up to 12-months follow-up from extracted exposure (birth, 3, 6 and 12-months) and outcomes variables (3 ,6 and 12-months)*  Results  **Paternal psychopathology and father involvement**   - Non-significant difference in paternal scores of responsibility in child care between fathers with a lifetime history of depression/ anxiety disorder *vs.* no such history (3, 6, 12-months)   **Maternal psychopathology and father involvement**   - Non-significant association between maternal depressive symptoms (continuous scores) and paternal reported responsibility (birth through 3-months, 3- through 6-months, 6- through 12-months) - Non-significant difference in paternal scores of responsibility in child care between mothers with a history of depression/anxiety disorder *vs.* no such history   (all time-points)  Study limitations:   - 47 fathers completed all measures up to 12-months - Lack of generalizability (homogenous sample, SES) - No absolute measure of fathers’ involvement (fathers’ responsibility measured in proportion to mother’s involvement) - No measure of paternal symptom levels concurrent with the paternal responsibility measure | |
|  |  |  |  |  |  | |
| **17. Ip et al. (2018)**  China  **Aim(s):** To investigate the risk factors for paternal postpartum depression and its  potential impact on father-infant attachment and infant development. | **Sample**  *Recruitment:*   - 772 expectant couples - Community sample - Recruitment at pregnancy from antenatal clinics   *Father socio-demographics:*   - Age, 91% 26 - ≥35 - 65% first time fathers - 55% higher education | **Tool extracted**  Paternal postnatal attachment Questionnaire (PPAQ; Condon et al., 2008)  **Main tool domain:** father-infant attachment  *Time point:* 6-weeks  *Time to complete:* n/r  *Total items:* 19  *Scoring format:*  -Likert scale: 5-points | **Paternal psychopathology**  Depressive symptoms  *(6-weeks)*  **Measure:** EPDS (Cox et al., 1987) | **Relationship quality**  Fathers attachment to the infant  *(6-weeks)*  The emotional bond or tie of affection experienced by  the parent towards the infant (PPAQ)  *Paternal behaviours*  -Patience and tolerance  -Pleasure in interaction  -Affection and pride  **Offspring outcomes**  Infant social and overall development *(6-months)*  **Measure:** Communication and Symbolic Behavior Scales Developmental Profile Infant-Toddler Checklist (CSBS–DP) (Wetherby & Prizant, 2002) | Cross-sectional and longitudinal analyses extracted  *Up to 2-month follow-up from extracted exposure (6-weeks) and outcomes variables 6-weeks, 6-months)*  Results  **Paternal psychopathology** **and father attachment to the infant**   - Increased paternal depressive symptoms were associated with lower scores in father-infant attachment sub-scales, including patience and tolerance (*β* = -.43, *p* < .001), pleasure in interaction (*β* = -.21, *p* = .002), and affection and pride at 6-weeks   (*β* = -.34, *p* < .001)  **Father attachment to the infant and offspring outcomes**   - Increased scores in fathers patience and tolerance (6-weeks) were associated with higher scores in infant overall development at 6-months   (*β* = .30, *p* = .02)   - Increased scores in fathers pleasure in interaction (6-weeks) were associated with higher scores in infant social (*β* = .29, *p* = .01) and overall development (*β* = .30, *p* = .02) at 6-months - Increased scores in fathers affection and pride (6-weeks) were associated with higher scores in infant social (*β* = .28, *p* = .02) and overall development (*β* = .31, *p* = .03) at 6-months - Non-significant association between fathers patience and tolerance sub-scale and infant social development   Study limitations   - Self-reported data – no use of direct clinical assessments and structured interviews - Retention rate was not high – increasing risk of bias - Relatively short follow-up period from exposure to outcome | |
|  |  |  |  |  |  | |
| **18. Kerstis et al., (2015)**  Sweden  **Aim(s):** To evaluate  associations between maternal and paternal depressive symptoms  and impaired bonding with their infant. | **Sample**  *Recruitment:*   - 727 couples - Community sample - Recruitment at pregnancy for women given birth at Uppsala University Hospital   *Father socio-demographics:*   - No paternal demographics were available as reported by the authors | **Tool extracted**  Postpartum Bonding Questionnaire (PBQ; Brockington et al., 2001)  **Main tool domain:**  Parent-infant bonding difficulties  *Time point:* 6-months  *Time to complete:* n/r  *Total items:* 25  *Scoring format:*   - Likert scale: 5-points | **Paternal psychopathology**  Depressive symptoms  *(6-weeks and 6-months)*  **Measure:** EPDS (Cox et al., 1987)  **Maternal psychopathology**  Depressive symptoms  *(6-weeks and 6-months)*  **Measure:** EPDS (Cox et al., 1987) | **Relationship quality**  Father-infant bonding difficulties *(6-months)*  The level of bonding difficulties between parent and infant *(PBQ)*  *Paternal behaviours*  -Impaired bonding | Cross-sectional and longitudinal analyses extracted  *4.5 month follow-up from exposure (6-weeks and 6-months) and outcomes variables (6-months)*  Results  **Paternal psychopathology** **and father-infant relationship quality**   - Increased paternal depressive symptoms at 6-weeks were associated with fathers impaired bonding with their infant at 6-months (*OR* = 1.16, CI = 1.07, 1.26) - Increased paternal depressive symptoms at 6-months were associated with fathers impaired bonding with their infant at 6-months (*OR =* 2.01, CI = 1.60–2.54*)*   **Maternal psychopathology** **and father-infant relationship quality**   - Increased maternal depressive symptoms at 6-weeks were associated with fathers impaired bonding with their infant at 6-months (*OR* = 1.45, CI = 1.10–1.90) - Increased maternal depressive symptoms at 6-months were associated with fathers impaired bonding with their infant at 6-months (*OR* 1.35, CI = 1.02–1.77)   Study limitations   - No objective measures of parenting – self-reports subject to rater bias | |
|  |  |  |  |  |  | |
| **19. Noh & Yeom (2017)**  South Korea  **Aim(s):**  To develop the Korean Paternal-Fetal Attachment Scale (K-PAFAS) to measure the level of attachment between the father and the expected baby, and to examine its validity and reliability | **Sample**  *Recruitment:*   - 230 men and their pregnant partners - Community sample - Recruitment at pregnancy through maternity clinics   *Father socio-demographics:*   - Age range, 80% 30-39 years - 94% were college educated | **Tool extracted**  Korean Paternal-Fetal Attachment Scale (K-PAFAS; Noh & Yeom, 2017)  **Main tool domain:** father-foetal attachment  *Time point:* antenatally  *Time to complete:* n/r  *Total items:* 20  *Scoring format:*   - Likert scale: 5-points | **Paternal psychopathology**  Depressive symptoms  *(measured antenatally)*  **Measure:** CES-D (Radloff, 1977) | **Relationship quality**  Fathers attachment to the foetus  *(measured antenatally)*  The strength of attachment between father and foetus  *(K-PAFAS)*  *Paternal behaviours*  -Bonding  -Paternal role  -Expectation  -Behavioural change  *Total scores of paternal behaviours related to fathers attachment to the foetus were examined in relation to parental psychopathology* | Cross-sectional analyses extracted  *Extracted exposure and outcome variables measured antenatally*  Results  **Paternal psychopathology and fathers attachment to the foetus**   - Higher paternal depressive symptoms were associated with lower total scores in fathers attachment to the foetus (*r* = -.18, *p* = .011)   Study limitations:   - Involvement measured via parent self-report – subject to rater bias - Convenience sample recruited from maternity hospitals holding prenatal education courses – families likely normal functioning and their responses may be biased | |
|  |  |  |  |  |  | |
| **20. Mezulis, Hyde & Clark (2004)**  USA  **Aim(s):** To explore whether father involvement in infancy may reduce or exacerbate  the well-established adverse effect of maternal depression during a child’s infancy on  behavior problems in childhood. | **Sample**  *Recruitment:*   - 550 mothers and partners - Community sample (mothers assessed for presence/absence depression at enrolment) - Recruited during pregnancy through antenatal clinics   *Father socio-demographics*  - All couples cohabiting  - All parents in employment  - English speaking | **Tool extracted**  Childrearing  Practices Report (CRPR; Block, 1965)  **Main tool domain:** parenting styles  *Time point:* 12-months  *Time to complete:* n/r  *Total items:* 28 (based on two factors, warmth and control)  *Scoring format:*   - Likert scale: 7-points | **Paternal psychopathology**  Depressive symptoms  *(1, 4, 12-months)*  **Measure:** CES-D (Radloff, 1977)  **Maternal psychopathology**  Clinical diagnosis of Major Depressive Disorder based on DSM-III-R *(1, 4, 12-months)*  **Measure:** DSM-III-R  (American Psychiatric Association, 1987)  Depressive symptoms  *(1, 4, 12-months)*  **Measure:** CES-D (Radloff, 1977)  *Note,* composite scores of psychopathology across infancy were used in the analysis | **Father involvement**  Quantity if time fathers spent interacting with their infant  *(12-months)*  Total hours spent with their infant, and in the absence of the mother (*Note,* measure was not an independent tool, but part of an external general questionnaire - not extracted)  **Relationship quality**  Parenting styles  *(12-months)*  Fathers reported on their parenting styles towards their infant (CRPR)  *Paternal behaviours*  -Warmth  -Control  **Offspring outcomes**  Child behaviour problems  *(Pre-school)*  **Measure:** Health and  Behavior Questionnaire (HBQ; Essex et al., 2002) | Longitudinal analyses extracted  *Up to 4+ year follw-up period from extracted exposure (1, 4, 12-months) and outcome variables (12-months – pre-school age)*  Results  **Paternal psychopathology and father involvement**   - Non-significant association between paternal depressive symptoms (across infancy) and father involvement (12-months)   **Paternal psychopathology and father-infant relationship quality**   - Increased levels of warmth (12-months) (*r* = .13, *p* = <.05) and control (*r* = .11, *p* < .05) were associated with higher paternal depressive scores across infancy   **Maternal psychopathology and father involvement**   - Non-significant association between maternal depression (diagnostic status and symptoms across infancy) and paternal involvement (12-months)   **Maternal psychopathology and father-infant relationship quality**   - Non-significant association between maternal depression (diagnostic status and self-report symptoms across infancy) and paternal warmth or control (12-months).   **Father involvement and offspring outcomes**   - Non-significant association between paternal depressive symptoms (across infancy) and father involvement (12-months)   **Father-infant relationship quality and offspring outcomes**   - Increased levels of paternal warmth (12-months) were associated with lower scores in child internalizing problems at pre-school (*r* = -.12, *p* < .05) - Non-significant association between paternal control (12-months) and child internalizing and externalizing behavioural problems at pre-school, and between paternal warmth (12-months) and child externalizing problems at pre-school   Study limitations   - CRPR originally intended for use with parents of toddlers and older children – not well validated in younger infants | |
|  |  |  |  |  |  | |
| **21. Nugent (1991)**  Republic of Ireland  **Aim(s):** To cxamine the involvement of a sample of working-class Irish fathers in infant caretaking  from the pre-, peri-, and postnatal periods to the end of the first year of life. Addionally,to study the relationship between paternal caretaking and infant cognitive development | **Sample**  *Recruitment:*   - 48 Irish fathers, their spouses, and infants - Community sample - recruited at birth at the National Maternal Hospital   *Father socio-demographics*  - All working-class SES  - Mean age, 26 years | **Tool extracted**  Unnamed (Nugent, 1991)  Analyses of father involvement in relation to offspring outcomes was examined using a combination of summary scores derived from interview data and items from the Father Caretaking Inventory (Nugent, 1987) at 1 and 12-months (Unnamed, Nugent, 1991)  **Main tool domain:** paternal involvement in child and childcare tasks  *Time point:* 1-12-months  *Time to complete:* n/a  *Total items:* n/a  *Scoring format:*   - Frequency counts | **Father involvement**  Fathers involvement child care activities *(1-12-months)*  Frequency of involvement in childcare activities (Unnamed, Nugent, 1991)  *Paternal behaviours*  -Attendance at prenatal class  -Presence at labour/delivery  -Fathers perception of role  -Fathers lifestyle change  -Involvement in feeding over  first month  -Involvement in nappy-changing in first month  *Total scores of father involvement were examined in relation to offspring outcomes* | **Offspring outcomes**  Cognitive and motor development *(12-months)*  **Measure:** Bayley Mental (MDI) and Motor (PDI) Scales of Infant Development (Bayley, Nancy, 1969) | Longitudinal analyses extracted  *11-month follow-up from extracted exposure (1 - 12-months) and outcome variables (12-months)*  Results  **Father involvement and offspring outcomes**   - Higher scores in fathers involvement in care giving over the first 12-months (combined score of data derived from 1 and 12-months) was associated with increased infant scores on the Bayley Mental Development Index at (12-months) year (*ß* = -.14, *p* = .04) - Non-significant association between father involvement (1-12 months) and infant scores on the Motor sub-scale of the Bayley (12-months)   Study limitations   - Relatively young sample of fathers – lack of generalizability | |
|  |  |  |  |  |  | |
| **22. Paulson et al. (2006)**  USA  **Aim(s):** To examine the effects of maternal and  paternal depression on parenting behaviors consistent with anticipatory guidance  recommendations. | **Sample**  *Recruitment:*   - 5089 mothers, fathers, and their infants - Community sample - recruited as part of a national birth cohort study   *Father socio-demographics:*   - Majority of fathers were between 20 - 35 years - 57% of white ethnicity, 8% of black, 15% Hispanic, 14% Asian, 6% others - 60% had college education or above - 86% in full employment | **Tool extracted**  Unnamed, Paulson et al. (2013)  **Main tool domain:**  Father engagement in childcare activities  *Time point:* 9-months  *Time to complete:* n/r  *Total items:* 7  *Scoring format:*   - Dichotomous scoring | **Paternal psychopathology**  Depressive symptoms  *(9-months)*  **Measure:** CES-D (Radloff, 1977)  **Maternal psychopathology**  Depressive symptoms  *(9-months)*  **Measure:** CES-D (Radloff, 1977) | **Father involvement**  Fathers positive engagement in activities (*9-months)*  Fathers report on how often they engage in enrichment and play activities with their child over the past week and past month (unnamed, Paulson et al, 2013)  *Paternal behaviours:*   - Enrichment activities   Reading to child  Telling stories  Singing to child   - Play activities   Playing peek-a-boo  Tickling child  Taking child on errands  Work/play outside with child  *Total scores of enrichment and play activities, as well as sub-scales of these behaviours (i.e., reading, playing peek-a-boo) were examined in relation to psychopathology* | Cross-sectional analyses extracted  *Extracted exposure and outcome variables measured at 9-months*  Results  **Paternal psychopathology and father involvement**   - Increased paternal depressive symptoms (continuous scores) were associated with a lower overall scores in fathers engagement in enrichment activities with their child (*ß* = -0.01, *p* < 0.05) - Non-significant association between paternal depressive symptoms and fathers overall engagement in play activities with their child - Fathers in the group who reported increased levels of depressive symptoms (CES-D cut-off) were less likely to play outside with their children, compared fathers in the group who reported decreased symptoms (*OR* = 1.42, *p* < .01) - Non-significant difference in how often fathers read, tell stories, sing, run errands or play peek-a-boo between fathers in the groups reporting increased *vs.* decreased depressive symptoms.   **Maternal psychopathology and father involvement**   - Non-significant association between maternal depressive symptoms and fathers overall engagement in either play or enrichment activities with their child - Fathers in the group whose partner reported increased levels of depressive symptoms (CES-D cut-off) were less likely to engage in singing with their child on a daily basis, compared fathers in the group whose partner reported decreased symptoms (*OR* = 1.39, *p* < .05). - Non-significant difference in how often fathers read, tell stories, run errands, play peek-a-boo or play outside with their child between mothers in the groups reporting increased *vs.* decreased depressive symptoms.   Study limitations   - No affirmed clinical diagnosis to measure depression - Parenting behaviours self-report – subject to rater bias - Narrow range of response options may provide a generally weak measure of the association between depression and parenting - Cross-sectional data analyses limiting inferences on causation | |
|  |  |  |  |  |  | |
| **23. Pisoni et al. (2015)**  Italy  **Aim(s):** To examine the links between parents’ prenatal attachment and psychosocial perinatal factors, such as maternal depression, anxiety and social support. | **Sample**  *Recruitment:*   - 80 couples - Two groups: 43 couples at risk of preterm delivery and 37 couples with no-risk pregnancy   *Father socio-demographics*  - Age range, 35 – 37-years  - Majority were married | **Tool extracted**  Paternal Antenatal Attachment Scale (PAAS; Condon et al., 1993)  **Main tool domain:** father-foetal attachment  *Time point:* antenatally  *Time to complete:* n/r  *Total items:* 16  *Scoring format:*  -Likert scale: 5-points | **Maternal psychopathology**  Depressive symptoms  *(measured antenatally)*  **Measure:** (CES-D; Radloff, 1977)  Anxiety symptoms  *(measured antenatally)*  **Measure:** State/Trait Anxiety Inventory (STAI; Spielberger et al., 1983) | **Relationship quality**  Fathers attachment to the foetus  *(measured antenatally)*  Fathers feelings, attitudes, and behaviours towards the foetus  (PAAS)  *Paternal behaviours*  -Quality of attachment  -Intensity of preoccupation  *Total scores of paternal behaviours related to fathers attachment to the foetus were examined in relation to psychopathology* | Cross-sectional analyses extracted  *Extracted exposure and outcomes both measured antenatally*  Results  **Maternal psychopathology and fathers attachment to the foetus**   - *Parents at risk for pre-term delivery group*: increased maternal depressive symptoms were associated with lower paternal scores in fathers attachment to the foetus (*trend significance, r* = -.28, *p* = 0.095) - *Parents experiencing a physiologic pregnancy group:* non-significant association between maternal depressive symptoms and paternal scores in fathers attachment to the foetus      - *Parents at risk for pre-term delivery group:* non-significant association between maternal anxiety symptoms paternal scores in fathers attachment to the foetus - *Parents experiencing a physiologic pregnancy group:* non-significant association between maternal anxiety symptoms paternal scores in fathers attachment to the foetus   Study limitations   - Lack of generalizability (homogenous sample, SES) - Paternal psychopathology was not measured - Cross-sectional data analyses limited inferences of causation | |
|  |  |  |  |  |  | |
| **24. Ragni et al. (2019)**  Italy  **Aim(s):** to explore the combined  effect of infants’ factors (temperament and sleep onset problems), and parental factors (parental mental  health in terms of post-partum affective disorders, consistency of bedtime routines and fathers’ involvement  at bedtime), on infants’ bedtime difficulties. | **Sample**  *Recruitment:*   - 66 two-parent families   *Father socio-demographics*  - Age range, 36 years | **Tool extracted**  Unnamed measure (ad hoc item relating to father involvement on the brief infant sleep questionnaire [BISQ])  **Main item domain:** father involvement in bedtime child care  *Time point:* 8-12 months  *Time to complete:* n/r  *Total items:* 1  *Scoring format:*   - Likert scale: 5-points | **Paternal psychopathology**  Overall level of affective disorders *(8-12 months)*  **Measure:** Perinatal assessment of paternal affectivity (PAPA; Baldoni et al., 2016)  **Maternal psychopathology**  Overall level of affective disorders *(8-12 months)*  **Measure:** Perinatal assessment of maternal affectivity (PAMA; Baldoni et al., 2016) | **Father involvement**  Fathers involvement in bedtime child care (*8-12 months)*  Fathers’ report on their level of involvement during bedtime child care (ad-hoc item attached to the Brief Infant Sleep  Questionnaire (BISQ; Sadeh et al., 2009))  **Offspring outcomes**  Infant sleep difficulties  *(8-12-months)*  Infant sleep difficulties as perceived by the father through self-report (BISQ; Sadeh et al., 2009)  Infant negative emotionality  *(8 and 12-months)*  Fathers reported on their infants temperament during different contexts.  **Measure:** QUIT (Axia, 2002) | Cross-sectional analyses extracted  *Extracted exposure and outcomes both measured at 8-12 months*  Results  **Paternal psychopathology and father involvement**   - Non-significant association between paternal involvement in bedtime child care and fathers level of overall affective disorders   **Maternal psychopathology and father involvement**   - Non-significant association between paternal involvement in bedtime child care and mothers level of overall affective disorders   **Father involvement and offspring outcomes**   - Increased paternal involvement in bedtime child care was associated with a high level of infants bedtime difficulties (as perceived by the father) (*β* = -.45, *p* = <.001) - Non-significant association between paternal involvement in bedtime child care and child negative emotionality   Study limitations   - Lack of generalizability (homogenous sample, SES) - Cross-sectional data analyses limited inferences of causation | |
|  |  |  |  |  |  | |
| **25. Seimyr et al. (2009)**  Sweden  **Aims(s):** To examine different aspects of maternal and paternal fetal attachment scales and investigate whether these scales are related to psychosocial factors and antenatal depressive mood. | **Sample**  *Recruitment:*   - 274 mothers and their partners - Community sample - recruitment at pregnancy through antenatal clinics   *Father socio-demographics:*  - Father demographics were not reported | **Tool extracted**  Paternal Fetal Attachment Scale (PFAS; Weaver & Cranley, 1983)  **Main tool domain:** father-foetal attachment  *Time point:* pregnancy  *Time to complete:* n/r  *Total items:* 23  *Scoring format:*   - Likert scale: 5-points | **Maternal psychopathology**  Depressive symptoms  *(measured antenatally)*  **Measure:** EPDS (Cox et al., 1987) | **Relationship quality**  Fathers attachment to the foetus  *(measured antenatally)*  Fathers’ report on their attachment behaviours to their unborn child (PFAS)  *Paternal behaviours*  -Interacting with the fetus  -Attributing characteristics  -Giving of self  -Differentiation from self  -Role taking  *Total scores of paternal behaviours and sub-domains of behaviour related to fathers attachment to the foetus were examined in relation to psychopathology* | Cross-sectional analyses extracted  *Extracted exposure and outcome variables measured antenatally*  Results  **Maternal psychopathology and fathers attachment to the foetus**     - Non-significant difference in fathers attachment to the foetus (total scores and sub-domains) between fathers whose partners reported increased (EPDS ≥ 10) *vs.* decreased depressive symptoms (EPDS < 10)   Study limitations:   - No objective measures of parenting, only self-report - Low response rate of 65% - Paternal demographics were not reported | |
|  |  |  |  |  |  | |
| **26. Shorey et al. (2018)**  Singapore  **Aims(s):** To explore factors influencing paternal involvement at 6-months postpartum and to examine longitudinal trends | **Sample**  *Recruitment:*   - 201 fathers accompanying partner during childbirth - Two groups: 109 first-time, 92 experienced (one or more child) - Community sample - recruited at birth through postnatal wards   *Father socio-demographics:*  - 47% Chinese, 34% Malay, 13% Indian, 5% of other ethnicity  - 98% in full-time work  - 52% had education above high school  - Mean age, 34 years | **Tool extracted**  Paternal Involvement Scale (PI; Rustia & Abbott, 1993)  **Main tool domain:** Fathers’ involvement in childcare duties  *Time point:* 6-months  *Time to complete:* n/r  *Total items:* 8  *Scoring format:*  -Likert scale: 5-points | **Paternal psychopathology**  Depressive symptoms  *(day of hospital discharge)*  **Measure:** EPDS (Cox et al., 1987) | **Father involvement**  Fathers involvement in child care activities (*6-months)*  Fathers indicate their degree of involvement in proportion to the mothers in childcare duties (e.g., playing with infant, diaper changing, dressing infant etc.) (PI)  *Total scores of father involvement were examined in relation to paternal psychopathology* | Longitudinal analyses extracted  *6-month follow-up from extracted exposure (day of hospital discharge) to outcome variables (6-months)*  Results  **Paternal psychopathology and father involvement**   - *Total sample of first-time and multiparous fathers*: higher paternal depressive symptoms (birth) were associated with increased scores in paternal involvement (6-months)   (*ß* = .18, *p* = .005)    Study limitations:   - No objective measures of involvement - self-report subject to rater bias / overreporting - Convenience sample recruited form one local hospital – limiting generalizability | |
|  |  |  |  |  |  | |
| **27. Tikotzky et al. (2010)**  Israel  **Aims(s):** To assess: (a) the involvement of fathers and mothers in overall  and night-time infant caregiving; (b) the links between paternal involvement in infant care and infant sleep  patterns during the first 6 months | **Sample**  *Recruitment:*   - 56 couples and their infants - Community sample -recruitment at pregnancy through hospital’s prenatal courses and adverts   *Father socio-demographics:*   - Mean age, 31-years - Average length spent in education was 16-years - Families were mostly of middle-upper class according to Israeli SES classifications | **Tool extracted**  Parental Involvement Questionnaire (Tikotzky et al., 2010)  **Main tool domain:** paternal involvement in childcare activities  *Time point:* 1 and 6-months  *Time to complete:* n/r  *Total items:* 10  *Scoring format:*  -Likert scale: 7 points | **Father involvement**  Fathers involvement in child care activities *(1-month)*  Father indicate their degree of involvement in proportion to the mother in childcare duties (e.g., feeding, bathing, playing, soothing, putting to sleep) (Parental Involvement Questionnaire. Tikotzky et al., 2010)  *Total scores of father involvement were examined in relation to offspring outcomes.*  *Note,* this total score included the average score of both parents – i.e., both mothers and fathers both filled in questionnaire separately, an average score for each pair of parents was computed and then for the total sample thus creating a combined score – with higher scores indicating increased paternal involvement. | **Offspring outcomes**  Infant sleep quality *(6-months)*  Infants sleep wake patterns assessed via a Micro Motionlogger actigraph and sleep diaries | Longitudinal analyses extracted  *5-month follow-up from extracted exposure (1-month) to outcome variables (6-months)*  Results  **Father involvement and offspring outcomes**   - Higher parental combined involvement (i.e., increased paternal involvement) (1-month) was associated with more consolidated infant sleep variables (6-months)   (*r*_sp_ **= -.**29 to -.38, *p* < .05)  Study limitations:   - Lack of generalizability (homogenous sample, SES) - Relatively short follow-up period from exposure to outcome | |
|  |  |  |  |  |  | |
| **28. Tikotzky et al. (2015)**  Israel  **Aims(s):** To examine triadic links between paternal involvement in infant caregiving and maternal and infant sleep | **Sample**  *Recruitment:*   - 57 couples and their infants - Community sample - Recruitment at pregnancy through hospital’s prenatal courses and adverts   *Father socio-demographics:*   - Mean age, 31-years - Families were mostly of middle-upper class according to Israeli SES classifications   *Note,* this study comprises the same sample as those included in Tikotzky et al. (2010) | **Tool extracted**  Parental Involvement Questionnaire (Tikotzky et al., 2010)  **Main tool domain:** parental involvement  *Time point:* 3 and 6-months  *Time to complete:* n/r  *Total items:* 10  *Scoring format:*  -Likert scale: 7 points | **Father involvement**  Fathers involvement in child care activities *(3-month)*  Father indicate their degree of involvement in proportion to the mother in childcare duties (e.g., feeding, bathing, playing, soothing, putting to sleep) (Parental Involvement Questionnaire. Tikotzky et al., 2010)  *Total scores of father involvement were examined in relation to outcomes.*  *Note,* this total score included the average score of both parents – i.e., both mothers and fathers both filled in questionnaire separately, an average score for each pair of parents was computed and then for the total sample thus creating a combined score – with higher scores indicating increased paternal involvement. | **Offspring outcomes**  Infant sleep  *(6-months)*  Infants sleep wake patterns assessed via a Micro Motionlogger actigraph and sleep diaries | Longitudinal analyses extracted  *3-month follow-up from extracted exposure (3-months) to outcome variables (6-months)*  Results  **Father involvement and offspring outcomes**   - Higher parental combined involvement (i.e., increased paternal involvement) (3-months) was associated with more consolidated infant sleep (6-months) (*r*_sp_ **=** -.35 to -.38, *p* < .05)   Study limitations:   - Lack of generalizability (homogenous sample, SES) - Relatively short follow-up period from exposure to outcome | |
|  |  |  |  |  |  | |
| **29. Wynter et al. (2016)**  Australia  **Aim(s):** to identify factors significantly associated with self-reported father-to-infant attachment at 6 months postpartum | **Sample**  *Recruitment:*   - 270 couples with a first infant - Community sample - recruitment during 2006 – 2007 in six local government areas   *Father socio-demographics*   - Mean age, 33-years - 72% were married - 35% had completed university education - 48% had a managerial or professional occupation | **Tool extracted**  Parental Attachment Questionnaire (PAQ; Condon & Corkindale, 1998)  **Main tool domain**: parent-infant attachment  *Time point:* 6-months  *Time to complete:* n/r  *Total items:* 19  *Scoring format:*  -Likert scale: 5-points | **Paternal psychopathology**  Depressive symptoms  *(6-months)*  **Measure:** EPDS (Cox et al., 1987) | **Relationship quality**  Fathers attachment to the infant  *(6-months)*  The emotional bond or tie of affection experienced by  the parent towards the infant  (PAQ)  *Paternal behaviours*  - Pleasure in proximity  - Acceptance and tolerance  - Competence as parent  *Total scores of paternal behaviours related to fathers attachment to the infant were examined in relation to psychopathology* | Cross-sectional analyses extracted  *Extracted exosure and outcome variables measured at 6-months*  Results  **Paternal psychopathology and fathers attachment to the infant**   - Increased paternal depressive symptoms were associated with lower total scores in fathers attachment to the infant (6-months) *(ß =* -.28, *p* = .02)   Study limitations   - Only partnered men were eligible to participate; future studies should include unpartnered or same sex partnered men. - 60 fathers lost to follow-up | |
|  |  |  |  |  |  | |
| **30. Vreeswijk et al. (2013)**  Netherlands  **Aim(s):** to explore fathers internal representations of their unborn baby and fathers attachment to the foetus, and their relationship with patenral psychological symptoms | **Sample**  *Recruitment:*   - 301 expectant fathers - Community sample - recruitment during partners pregnancy through midwifery practices   *Father socio-demographics*   - Mean age, 34-years - 64% were college educated - 97% were employed | **Tool extracted**  Paternal Antenatal Attachment Scale (PAAS; Condon, 1993)  **Main tool domain:** father-foetal attachment  *Time point:* antenatally  *Time to complete:* n/r  *Total items:* 16  *Scoring format:*  -Likert scale: 5-points | **Paternal psychopathology**  Depressive symptoms  *(measured antenatally)*  **Measure:** EPDS (Cox et al., 1987)  Anxiety symptoms  *(measured antenatally)*  **Measure:** STAI (Spielberger et al., 1983) | **Relationship quality**  Fathers attachment to the foetus  *(measured antenatally)*  The feelings, attitudes, and behaviours towards the foetus.  (PAAS)  *Paternal behaviours*  -Quality of attachment  -Intensity of preoccupation  *Total scores and subscales of paternal behaviours related to fathers attachment to the foetus were examined in relation to psychopathology* | Cross-sectional analyses extracted  *Exposure and outcomes both measured antenatally*  Results  **Paternal psychopathology and fathers attachment to the foetus**   - Increased paternal antenatal depressive symptoms were associated with lower scores in the quality of fathers attachment to the foetus (*r* = -.14, *p* < .05) - Non-significant association between paternal antenatal depressive symptoms and total scores in fathers attachment to the foetus, or fathers intensity of preoccupation - Increased paternal antenatal state *(r* = -.17, *p* < .01) and trait *(r* = -.22, *p* < .01) anxiety symptoms were associated with lower scores in the quality of fathers attachment to the foetus - Increased paternal antenatal trait anxiety symptoms were associated with lower total scores in fathers attachment to the foetus *(r* = -.15, *p* < .05) - Non-significant association between paternal antenatal state anxiety symptoms total scores in fathers attachment to the foetus - Non-significant association between paternal antenatal state or trait anxiety symptoms and fathers intensity of preoccupation   Study Limitations   - Sample primarily highly educated Dutch fathers – lack of generalizability - Only antenatal measurement - results cannot be generalized to the postnatal period | |
