# Supplementary material for: The Applicability and Performance of Tools Used to Assess the Father-Offspring Relationship in Relation to Parental Psychopathology and Offspring Outcomes
Source: Front Psychiatry. 2021 Jan 5;11:596857. doi: 10.3389/fpsyt.2020.596857 (PMC7814871; doi:10.3389/fpsyt.2020.596857)
Supplement: Supplementary file 6 [file Table_6.docx]

| **Supplementary Materials_Table 6**  Descriptive characteristics of studies utilising interview tools to assess the father-infant relationship – including relationship quality and father involvement | | | | | |
| --- | --- | --- | --- | --- | --- |
| **Publication / Country/ Aim(s)** | **Sample details** | **Interview-based tool** | **Type of exposure variable(s)**  *(Paternal and /or maternal psychopathology, father-infant relationship)* | **Type of outcome variable(s)**  *(Father-infant relationship variables, offspring outcomes)* | **Data analysis/ Results /Limitations** |
|  | | | | | |
| **Interview tools (*n* = 4) extracted from *n* = 4 publications** | | | | | |
|  | | | | | |
| **1. Frodi et al. (1983)**  Sweden  **Aims (s):** To examine the effects of parental gender and caretaking role on parental attitudes,  parent-infant interaction, infant-parent attachment, and infants' sociability with strangers  and their parental preferences. | **Sample**  *Recruitment:*   - 51 couples - Community sample -recruited at childbirth and parent-preparation classes   *Father socio-demographics*   - Mean age, 30-years - Majority well educated - Majority middle-class SES | **Tool extracted**  Unnamed tool (Frodi et al., 1983)  **Main tool domain:**  involvement in caretaking and with the child  *Time-point:* 5-months  *Set-up*: at the family’s home  *Scoring format:*   - Time duration - estimated time in active interaction   *Interviewer characteristics:*  -n/r | **Father involvement**  Father involvement in child care activities *(5-months)*  Quantity of paternal engagement in caretaking and involvement with the child (unnamed postnatal interview, Frodi et al., 1983) | **Offspring outcomes**  Infant-attachment security  *(11 and 13-months)*  Infant attachment styles were classified during observations of infant–father strange situations  *Infant attachment styles*  -Secure attachment style  -Insecure attachment style  **Measure:** Strange Situation Procedure (SSP; Ainsworth et al., 1978) | Longitudinal data analyses  *6-8-month follow-up from extracted exposure (5-months) to outcome variables (11 and 13-months)*  Results  **Father involvement and offspring outcomes**  *Paternal direct engagement*   - Non-significant association between paternal reported involvement in child care activities (5-months) and infant-attachment security (11 and 13-months)   Study limitations:   - Lack of generalizability (homogenous sample, SES) - Relatively short follow-up period from exposure to outcome |
|  |  |  |  |  |  |
| **2. Goodman et al. (2014)**  USA  **Aims(s):** To examine concurrent and prospective associations between maternal depression and father involvement - to evaluate support for the spillover model -i.e., higher depressive symptom levels associated with lower father involvement, and the compensatory buffering model- i.e., higher depressive symptom levels associated with higher father involvement. | **Sample**  *Recruitment:*   - 129 families with consenting fathers - Women at risk for perinatal depression recruited at pregnancy through referrals of local obstetrical and mental health practitioners - Potential participants completed checklist and telephone interview - At enrolment, women were included if they fulfilled diagnostic criteria for lifetime history of depression or anxiety. Their partners were invited to participate (50% response) - Final sample included 92 fathers participated with data 3-months, 85 at 6-months, and 74 at 12-months   *Father socio-demographics:*   - Completed an average of 16 years education   - 94% were married   - 90% European America, 9% African American   -Mean age, 36 years   - 21% of fathers had a lifetime history of depression | **Tool extracted**  Child Development Supplement to Panel Study of Income Dynamics Time Diary (CDS; Hofferth et al. 1997)  **Main tool domains:** paternal engagement and accessibility in childcare  *Time point:* 3, 6, 12-months  *Set-up*: over the telephone  *Interview duration*: n/r  *Scoring format:*  -Total time duration (hours)  -Likert scale: 10-points  *Interviewer characteristics:*  -n/r | **Maternal psychopathology**  Depressive symptoms  *(3, 6 and 12-months)*  **Measure:** BDI (Beck, 1978)  Lifetime history of depression or anxiety disorder  **Measure:** DSM-IV-R (SCID; First et al., 2002)  **Paternal psychopathology**  Lifetime history of depression or anxiety disorder  **Measure:** DSM-IV-R (SCID; First et al., 2002)  *Note: paternal and maternal history of depression or anxiety was not a main exposure variable in this study. They were included as main potential confounding variables examined in relation to paternal involvement* | **Father involvement**  Fathers direct engagement in positive activities and in routine childcare tasks  *(3, 6 and 12-months)*  Fathers report on their involvement in childcare activities over a 24-hour period during the previous weekday (workday) and a recent weekend day (non-work day) (CDS, Hofferth et al. 1997)  *Paternal behaviours:*   - Average hours of engagement - Time spent in one-to-one activity with child - Time father is available | Longitudinal data analyses  *Extracted exposure and outcome variables both measured at 3 ,6 and 12-months*  Results  **Maternal psychopathology** **and father involvement**   - Increased maternal depressive symptoms (birth through 3-months) were associated with increased levels of paternal reported weekday (*β* = .28, *p* < .01) and weekend engagement (*β* = .25, *p* < .05) (3-months) - Increased maternal depressive symptoms (4- through 6-months) were associated with increased levels of paternal reported weekend engagement (*β* = .65, *p* < .01) (12-months) - Increased maternal depressive symptoms (4- through 6-months) were associated with increased levels of paternal reported weekday (*β* = .46, *p* < .05) and weekend accessibility (*β* = .77, *p* < .01) (12-months) - In contrast, increased maternal depressive symptoms (7- through 12-months) were associated with *lower* levels of paternal reported weekday (*β* = -.48, *p* < .05) and weekend accessibility (*β* = -.77, *p* < .01) (12-months) - Increased maternal depressive symptoms were associated with paternal weekend engagement (12-months) (concurrently) (*r* = .28, *p* > .05) - Non-significant associations between maternal depressive symptoms and paternal accessibility or weekday engagement (concurrently at 3, 6, 12-months) - Non-significant difference in paternal accessibility or engagement between mothers in the group with a lifetime history of depression *vs.* no history (concurrently at 3, 6, 12-months)   **Paternal psychopathology** **and father involvement**   - Paternal lifetime history of depression was associated with fathers’ weekend (F(1, 67) = 4.10, *p* < .05) and weekday accessibility (F(1, 69) = 4.57, *p* < .05) *(bivariate correlations reported)*   Study limitations:   - 47 fathers completed all measures up to 12-months - Lack of generalizability (homogenous sample, SES) - 50% response rate for fathers - No measure of paternal depressive symptom levels concurrent with father’s involvement |
|  |  |  |  |  |  |
| **3. Hall et al. (2014)**  Netherlands  **Aim(s):** To examine whether the quality of early paternal representations is associated with later quality of paternal and infant interactive behavior, and whether paternal interactive behavior mediates the relation between paternal representations and infant's development. | **Sample**  *Recruitment:*   - 220 fathers, partners, and their infants - Two groups: 71-full term infants, 118 preterm infants - Recruited at birth in local hospitals   *Father socio-demographics:*   - 77% had completed higher secondary education - Mean age, 35-years - 64% were first-time fathers | **Tool extracted**  Working Model of Child Interview (WMCI; Zeanah et al., 1986)  **Main tool domain:**  paternal attachment representations  *Time point:* 6-months  *Set-up:* at the family home  *Interview duration*: 45-mins  *Scoring format:*  -Transcripts rated on a  5-point Likert scale  *Rater characteristics:*  - Trained coders (until 80%  reliability was reached) | **Father-infant relationship quality**  Paternal attachment representations of the infant  *(6-months)*  Fathers report on their subjective experiences and perceptions of their child, caregiving behaviour and his relationship with the child (WMCI):  *Attachment representations:*  -Balanced representations  -Disengaged representations  -Distorted representations | **Offspring outcomes**  Infant language development  *(24-months)*  **Measure:** Peabody Picture Vocabulary Test **(**PPVT; Dunn & Dunn, 2004) | Longitudinal data analyses  *18-month follow-up from exposure (6-months) to outcome (24-months)*  Results  **Father-infant relationship quality and offspring outcomes**  **.**   - Non-significant association between overall paternal attachment representations (6-months) and infants vocabulary scores (24-months) (*ns* direct effect of mediation model)      - Fathers in the group with balanced attachment representations (6-months) had infants with higher scores in vocabulary (24-months), compared to fathers in the group with distorted representations (*p* = .002) - Non-significant difference in infant vocabulary scores (24-months) between fathers with balanced and disengaged attachment representations (6-months) - Non-significant difference in infant vocabulary scores (24-months) between fathers with disengaged and distorted attachment representations (6-months)   Study limitations:   - Lack of generalizability (homogenous sample with regards SES) - 14% attrition at first measurement   (6-months); 39 fathers were lost to follow-up at 24-months.   - Extracted analyses based on 150 fathers (62 term, 88 pre-term infants) - No norms on the PPVT at this age group - raw scores used (i.e., correct number of items) |
|  |  |  |  |  |  |
| **4. Jia et al. (2016)**  USA  **Aims(s):** To examine associations between parents’ psychological adjustment and their own and their partners’ parental involvement time in a sample of dual-earner, first-time parents from the third trimester of pregnancy through 3, 6, and 9 months postpartum. | **Sample**  *Recruitment:*   - 177 first-time, working parents (biological parents) - Community sample   *Father socio-demographics:*  - 87% were married  - 72% completed a bachelor’s degree or above  - Mean age, 30 years  - 86% of couples were of white ethnicity, 6% black and 3% Asian | **Tool extracted**  Parental Involvement Time Diary (Jia et al., 2016)  (modelled based on the American Time Use Survey, (U.S. Bureau of Labor Statistics, 2006)  **Main tool domain**  paternal engagement in enrichment activities and physical care activities  *Time point:* 3, 6, 9-months  *Set-up:* home at 3-months, phone at 6 and 9-months  *Duration*: not described  *Scoring format:*  -Total time (hrs)  *Rater characteristics:*  -Trained research assistants | **Paternal psychopathology**  Dysphoria symptoms  *(antenatally, 3, 6, 9-months)*  **Measure:** Dysphoria survey (Bonomi et al. 2008)  Anxiety symptoms  *(antenatally, 3, 6, 9-months*  **Measure:** STAI (Spielberger et al., 1983)  **Maternal psychopathology**  Dysphoria symptoms  *(antenatally, 3, 6, 9-months)*  **Measure:** Dysphoria survey (Bonomi et al. 2008)  Anxiety symptoms  *(antenatally, 3, 6, 9-months)*  **Measure:** STAI (Spielberger et al., 1983) | **Father involvement**  Fathers involvement in child care activities  *(3, 6 and 9-months)*  Parents reported a sequential list of activities during one recent workday and non-workday over a 24-hour period (Parental Involvement Time Diary, Jia et al., 2016)  *Paternal behaviours*   - Engagement in enrichment activities (e.g., direct involvement in activities, such as reading, playing, talking) - Engagement in routine childcare activities (e.g., direct involvement in physical care, such as feeding, diaper changing, giving baths) | Longitudinal data analyses  *3-9-month follow-up from exposure (antenatally, 3, 6, 9-months) and outcome variables (3,6,9-months)*  Results  **Paternal psychopathology and father involvement**  *Father involvement in activities*   - Increased paternal reported antenatal dysphoria symptoms were associated with a decrease in their own non-workday engagement in activities (*β* =−.49, *p* < .05) - Non-significant association between paternal antenatal dysphoria symptoms and paternal workday engagement in activities and (3 to 9-months) - Non-significant association between paternal postnatal dysphoria symptoms and paternal workday and non-workday engagement in childcare activities (through 3 to 9-months) - Non-significant association between paternal postnatal anxiety symptoms and paternal workday and non-workday engagement in childcare activities (through 3 to 9-months)   *Father involvement in child care*   - Non-significant association between paternal antenatal or postnatal dysphoria symptoms and paternal workday and non-workday engagement in physical childcare (through 3 to 9-months) - Increased paternal postnatal anxiety symptoms were associated with a decrease in their workday engagement in childcare   (*β* = −.08, *p* = .06) (through 3 to 9-months)   - Non-significant association between paternal postnatal anxiety symptoms and paternal non-workday engagement in physical childcare (through 3 to 9-months) - Non-significant association between paternal antenatal anxiety symptoms and paternal workday and non-workday engagement in physical childcare (through 3 to 9-months)   **Maternal psychopathology and father involvement**  *Father involvement in activities*   - Increased maternal antenatal anxiety symptoms were associated with an increased level of paternal reported non-workday engagement in activities with their child (*β* = .17, *p* < .01) - Non-significant association between maternal reported antenatal anxiety symptoms and the level of paternal reported workday engagement in activities with their child - Non-significant association between maternal postnatal anxiety symptoms and the level of paternal reported workday and non-workday engagement in child activities (through 3 to 9-months) - Non-significant association between maternal antenatal and postnatal dysphoria symptoms and the level of paternal reported workday and non-workday engagement in child activities (through 3 to 9-months)   *Father involvement in child care*   - Increased maternal antenatal anxiety symptoms were associated with an increase in the level of paternal reported workday (*β* = .11, *p* < .05) and non-workday (*β* = .15, *p* < .01) engagement in childcare - Non-significant association between maternal postnatal anxiety symptoms and the level of paternal reported workday and non-workday engagement in childcare (through 3 to 9-months) - Non-significant association between maternal reported antenatal and postnatal dysphoria symptoms and the level of paternal reported workday and non-workday engagement in childcare (through 3 to 9-months)   Study limitations:   - Time diary has a limited in capacity to examine quality of involvement - Other characteristics not examined, such as child gender and temperament which may affect associations between psychological functioning and involvement - Lack of generalizability (homogenous sample, SES) |
