# Supplementary material for: The Applicability and Performance of Tools Used to Assess the Father-Offspring Relationship in Relation to Parental Psychopathology and Offspring Outcomes
Source: Front Psychiatry. 2021 Jan 5;11:596857. doi: 10.3389/fpsyt.2020.596857 (PMC7814871; doi:10.3389/fpsyt.2020.596857)
Supplement: Supplementary file 7 [file Table_7.docx]

| **Supplementary Materials_Table 7**  Validity evidence supporting observational tools (*n* = 16) used to assess father-infant relationship quality | | | | | | | |
| --- | --- | --- | --- | --- | --- | --- | --- |
| **Observational tool /**  **Target population^a^ / Training** | **Related publication(s)** | **Internal structure^b^** | **Content validity^a^** | **Response process^c^** | **Relations with other variables**  Convergent, Discriminant, Criterion Validity  *(1) = significant association; (0) = non-significant association* | | |
| **Observational tools developed in maternal samples (*n* = 9)** | | | | | | | |
|  | | | | | | |  |
| **Ainsworth Maternal Sensitivity Scales (AMSS; Ainsworth et al. 1974)**  ----------------------------------  *Initial target population*  Tool initially developed in a community sample of mothers and their infants (Ainsworth et al., 1974)  *Training and availability*  -Coding manual available  -Self-guided training | **Lucassen et al. (2017)** | *Inter-rater reliability*   - On a sub-sample of   *n* = 82, ICC = 0.78 for the overall measure in fathers  *Inter-scale item / domain correlations:* n/r  *Factor analysis:* n/r  *Test-retest reliability:* n/r | N/A | N/A | **Criterion validity (offspring outcomes):**   - **(0) -** Concurrent**:** paternal interactive behaviour (i.e., sensitivity) → infant-attachment security (14-months); *ns* | | |
|  |  |  |  |  |  | | |
| **Assessment of Mind-Mindedness (Meins et al. 2001)**  ---------------------------------  *Initial target population*  Tool initially developed in a community sample of mothers and their infants observed at 6-months (Meins et al., 2001)  *Training and availability:*  -Coding manual available   - Self-guided training - 2-day training course from the developers | **Arnott & Meins (2007)** | *Inter-rater reliability*   - On a sub-sample of 20%, **ϰ** = 0.73, for the overall measure   *Note:* reliability reported across mothers and fathers, not specifically fathers  *Inter-scale item / domain correlations:*   - Within-scale correlations between appropriate and inappropriate mind-related comments   *(r* = 0.55. *p* < .025)  *Factor analysis:* n/r  *Test-retest reliability:* n/r | N/A | N/A | **Criterion validity (offspring outcomes):**   - **(1) -** Predictive: paternal speech (i.e., proportion of appropriate paternal mind–related comments) (6-months) → infant-attachment security (15-months); large *es* - **(0) –** Predictive: paternal speech (i.e., proportion of inappropriate paternal mind-related comments) (6-months) → infant-attachment security (15-months); *ns* | | |
|  |  |  |  |  |  | | |
|  | **Lundy (2003)** | *Inter-rater reliability*   - *r* = 0.77 – 0.83 across all verbal domains   *Note:* reliability reported across mothers and fathers, not specifically fathers  *Inter-scale item / domain correlations :*n/r  *Factor analysis:* n/r  *Test-retest reliability:* n/r | N/A | N/A | **Criterion validity (offspring outcomes):**   - **(1) -** Predictive**:** paternal speech (i.e., comments related to infant thought processes) (6-months) → infant-attachment security (13-months); large *es* - **(0) -** Predictive**:** paternal speech (i.e., comments related to infant emotional engagement and problem solving) (6-months) → infant-attachment security (13-months); *ns*   **Convergent validity (severity of psychological symptoms):**   - **(0) -** Paternal depressive symptoms: → paternal speech (i.e., thought processes comments, emotional engagement and problem solving comments) (6-months); *ns* | | |
|  |  |  |  |  |  | | |
|  | **Miller et al., (2019)** | *Inter-rater reliability*   - **ϰ** = 0.65 – 0-.95, across parent verbal domains   *Note:* reliability reported across mothers and fathers, not specifically fathers  *Inter-scale item / domain correlations:*   - Paternal appropriate mind-related comments → paternal non-attuned mind-related comments (*r* = .34, *p* < .001)   *Factor analysis:* n/r  *Test-retest reliability:* n/r |  |  | **Criterion validity (offspring outcomes):**   - **(1)** Predictive - paternal verbal behaviours: (i.e., attuned mind-related comments) (7-months) → offspring attachment security at 24-months (medium *es*) and 10-years (small – medium *es*). - **(0)** Predictive - paternal verbal behaviours: (i.e., non-attuned mind-related comments) (7-months) → offspring attachment security at 24-months and 10-years; *ns* | | |
|  |  |  |  |  |  | | |
| **Behaviour-State System (Cohn et al., 1986)**  ----------------------------------  *Initial target population*  Tool initially developed in a sample of mothers with increased levels of depressive symptoms, observed with their infants under 12-months (Cohn et al., 1986)  *Training and availability*   - Behaviour-State System described in a published article (Cohn et al., 1986) - Self-guided training | **Chabrol et al. (1996)** |  | N/A | N/A | **Discriminant validity (parent clinical diagnostic groups):**   - **(0) -** Maternal clinical diagnostic groups**:** presence *vs.* absence of depression → paternal interactive behaviours (i.e., time spent in an eliciting or play state) and affect (i.e., negative affective state) (3-6 months); *ns* - **(0) -** Maternal clinical diagnostic groups**:** dyadic interactive behaviours (i.e., time spent in a shared eliciting or play state) (3-6 months) and affect (i.e., negative, and neural state); *ns* | | |
|  |  |  |  |  |  | | |
| **Categorical System for Micro-Analysis of the Early Mother–Child Interaction (Jorg et al. 1994)**  -----------------------------------  *Initial target population*  Tool initially developed in a sample of mothers. Original source was not available in English – no further details (Jorg et al., 1994)  *Training and availability:*   - Published article on the tool (Jorg et al., 1994)   -Self-guided training | **Trautmann-Villalba et al. (2006)** | *Inter-rater reliability*   - **ϰ** = 0.70, for the overall measure in fathers (sub-sample, n/r)   *Inter-scale item / domain correlations:* n/r  *Component analysis*   - Three paternal factors and two infant factors identified   *Test-retest reliability:* n/r | N/A | N/A | **Criterion validity (offspring outcomes):**   - **(1)** Predictive - paternal interactive behaviours: (i.e., sensitivity and responsiveness) (3-months) → externalizing behavioural problems (CBCL score > 60 *vs.* < 60) (8-11 years); medium *es* - **(0)** Predictive - paternal interactive affect: (i.e., positive emotionality) (3-months) → externalizing behavioural problems (CBCL score > 60 *vs.* < 60) (8-11 years); *ns* - **(1)** Predictive - infant interactive affect: (i.e., positive emotionality) (3-months) → externalizing behavioural problems (CBCL score > 60 *vs.* < 60) (8-11 years); small to medium *es* - **(0)** Predictive - infant interactive affect: (i.e., negative emotionality) (3-months) → externalizing behavioural problems (CBCL score > 60 *vs.* < 60) (8-11 years); *ns* | | |
|  |  |  |  |  |  | | |
| **CARE-Index (infant form; Crittenden, 2004)**  ----------------------------------  *Initial target population*  Tool initially developed in a sample of mothers – with several versions available for different infant age groups (Crittenden, 2004)  *Training and availability:*   - Unpublished training manual - Training required from the developers | **Parfitt et al. (2013)** | *Inter-rater reliability*   - On a sub-sample of 12%, ICC = 0.70 – 0.84, across parent domains - On a sub-sample of 12%, ICC = 0.85 – 0.92, across infant domains   *Note: inter-rater reliability reported across mothers and fathers, not specifically fathers*  *Inter-scale item / domain correlations:* n/r  *Factor analysis:* n/r  *Test-retest reliability:* n/r | N/A | N/A | **Convergent validity (with a measure of maternal parenting):**   - **(1) –** Paternal interactive behaviours (i.e., control and sensitivity) → maternal interactive behaviours (same sub-scales) (3-months); medium (control), large *es* (sensitivity) - **(0) –** Paternal interactive behaviours (i.e., unresponsiveness) → maternal interactive behaviours (same sub-scale) (3-months); *ns*   **Convergent validity (severity of psychological symptoms):**   - **(0) -** Paternal depressive/anxiety symptoms: → paternal interactive behaviours (i.e., unresponsiveness and control) (3-months); *ns* - **(0) -** Paternal PTSD symptoms: → paternal interactive behaviours (3-months) *ns* - **(1) -** Paternal depressive/anxiety symptoms: → infant interactive behaviours (i.e., difficulty) (3-months); large *es* - **(0) -** Paternal depressive/anxiety symptoms: → infant interactive behaviours (i.e., passivity) (3-months); *ns* - **(0) -** Paternal PTSD symptoms: → infant interactive behaviours (3-months); *ns* - **(1) -** Maternal depressive/anxiety symptoms: → paternal interactive behaviours (i.e., control and unresponsiveness) (3-months); medium *es* - **(0) -** Maternal PTSD symptoms: → paternal interactive behaviours (3-months); *ns* - **(0) -** Maternal depressive/anxiety symptoms: → infant interactive behaviours (3-months); *ns* - **(0) -** Maternal PTSD symptoms: → infant interactive behaviours (3-months); *ns* | | |
|  |  |  |  |  |  | | |
|  | **Fuertes et al., (2016)** | *Inter-rater reliability*   - On a sub-sample of 20%, ICC = 0.97 – 0.98, across father behavioural domains   *Inter-scale item / domain correlations:* n/r  *Factor analysis:* n/r  *Test-retest reliability:*   - Non-significant association between sensitivity at 9-months and 15-months | N/A | N/A | **Criterion validity (offspring outcomes):**   - **(1) -** Predictive: paternal interactive behaviours (i.e., sensitivity and control) (9-months) → infant secure attachment (12-months); es n/r - **(0) –** Predictive: paternal interactive behaviours (i.e., sensitivity and control) (9-months) → infant secure attachment (18-months); *ns*   **Convergent validity (with a measure of maternal parenting):**   - **(1) -** Paternal interactive behaviours (i.e., sensitivity) → maternal interactive behaviours (same sub-scale) (9-months); large *es* | | |
|  |  |  |  |  |  | | |
| **Competing demands task (Smith & Pederson, 1988)**  ----------------------------------  *Initial target population*  Tool developed in a sample of mothers and their 12-month old infants  *Training and availability:*   - Published article describing the measure (Smith & Pederson, 1988) - Self-guided training | **Brown, Mangelsdorf & Neff (2012)** | *Inter-rater reliability*   - On a sub-sample of 15%, γ = 0.93 (mean), for the overall measure in fathers   *Inter-scale item / domain correlations:* n/r  *Factor analysis:* n/r  *Test-retest reliability:*   - Non-significant association between sensitivity at 13-months and 3-years | N/A | N/A | **Criterion validity (offspring outcomes):**   - **(0) -** Concurrent: paternal interactive behaviours (i.e., sensitivity) → securely *vs.* insecurely attached infants (13-months); *ns*   **Convergent validity (another measure of paternal parenting):**   - **(0)** *–* Paternal sensitivity (competing demands task) → paternal involvement (*via* a composite measure of the PRS and time-diary methods) (3-years); *ns* | | |
|  |  |  |  |  |  | | |
| **Global Rating Scales (GRS; Murray et al. 1996; Gunning & Murray, 2002)**  ------------------------------  *Initial target population*  Tool initially developed in a community sample of mothers screened for depressive symptoms and then diagnosed with major depression and low-risk controls, and their infants at 2 - 5 months (Murray et al., 1996)  *Training and availability:*   - Unpublished training manual - Training course available from the developers | **Koch et al. (2019)** | *Inter-rater reliability*   - On a sub-sample of 30%, **ϰ** = 0.50 – 0.70, across all domains in fathers   *Inter-scale item / domain correlations:* n/r  *Factor analysis:* n/r  *Test-retest reliability:* n/r | N/A | N/A | **Discriminant** **validity (parent clinical diagnostic groups):**   - **(1) -** Paternal clinical diagnostic groups: presence *vs.* absence of clinical depression → lower levels of paternal interactive behaviour (i.e., responsiveness and sensitivity) and increased levels of affect (i.e., depressive affect) (2-16 weeks) - **(1) -** Paternal clinical diagnostic groups: presence *vs.* absence of clinical depression → lower levels of infant interactive behaviour (i.e., attention) (2-16 weeks) - **(0) -** Paternal clinical diagnostic groups: → infant interactive affect (i.e., negative affect); *ns* - **(0) -** Paternal clinical diagnostic groups: → dyadic interactive behaviour (i.e., quality of interaction); *ns* | | |
|  |  |  |  |  | | | |
|  | **Ramchandani et al. (2013)** | *Inter-rater reliability*   - On a sub-sample of 20%, ICC = 0.74- 0.88, across father domains   *Inter-scale item / domain correlations:*   - Paternal mean scores in remote behaviours → paternal intrusiveness   *(r* =.25, *p* = .018)  *Factor analysis*: n/r  *Test-retest reliability*: n/r | N/A | N/A | **Convergent validity (with a measure of maternal parenting):**   - **(1) -** Paternal interactive behaviours (i.e., remoteness) and affect (i.e. depressive affect) → maternal interactive behaviours (same sub-scales); medium (remoteness), small *es* (depressive affect). - **(0) -** Paternal interactive behaviours (i.e., sensitivity, intrusiveness) → maternal interactive behaviours (same sub-scales); *ns*   **Criterion validity (offspring outcomes):**   - **(1) -** Predictive: paternal interactive behaviours (i.e., disengaged and remote behaviours) during father-infant *floor-mat interactions* (3-months) → infant externalising behavioural problems (12-months); medium *es* - **(0) -** Predictive: paternal interactive behaviours (i.e., sensitivity) and affect (i.e., depressive affect) during father-infant *floor-mat interactions* (3-months) → infant externalising behavioural problems (12-months); *ns* - **(0) -** Predictive: paternal interactive behaviours and affect during father-infant *seat interactions* (3-months) → infant externalising behavioural problems (12-months); *ns* | | |
|  |  |  |  |  | | | |
|  | **Sethna et al. (2015)** | *Inter-rater reliability*   - On a sub-sample of 20%, ICC = 0.74 – 0.88, across father domains   *Inter-scale item / domain correlations:*   - Paternal sensitivity → l less intrusiveness (*r* = .33, *p* = < .01; infant seat) (*r* = .41, *p* < .01; floor-mat) - Paternal remoteness → less intrusiveness (*r* = .26, *p* < .01; floor-mat) - Paternal depressive affect → insensitivity (*r* = -.30, *p* = < .01; infant seat) (*r* = -.32, *p* = < .01) and remoteness (*r* = -..55, *p* = < .01; infant seat) (*r* = -.56, *p* < .01; floor-mat)   *Factor analysis*: n/r  *Test-retest reliability*: n/r | N/A | N/A | **Discriminant validity (parent clinical diagnostic groups):**   - **(0) -** Paternal clinical diagnostic groups: presence *vs.* absence of depression → lower levels of paternal interactive behaviour (i.e., intrusiveness) during father-infant *floor-mat interactions* (3-months); *(marginally significant, p* = 0.05) - **(0) -** Paternal clinical diagnostic groups**:** → paternal interactive behaviours (i.e., sensitivity and remoteness) and affect (i.e., depressive affect) during father-infant *floor-mat interactions* (3-months); *ns* - **(0)** Paternal clinical diagnostic groups**:** → paternal interactive behaviours during father-infant *seat interactions* (3-months); *ns*   **Convergent validity (severity of psychological symptoms):**   - **(0) -** Maternal depressive symptoms**:** → paternal interactive behaviours and affect during father-infant floor-mat or infant seat interactions (3-months); *ns* | | |
|  |  |  |  |  |  | | |
|  | **Sethna et al., (2019)** | *Inter-rater reliability*   - On a sub-sample of 20%, ICC = 0.96 for the overall measure of paternal sensitivity   *Inter-scale item / domain correlations*: n/r  *Factor analysis*: n/r  *Test-retest reliability*: n/r | N/A | N/A | **Criterion validity (offspring outcomes):**   - **(1) -** Concurrent: paternal interactive behaviours (i.e., sensitivity) during father-infant interactions → infant cerebellar volumes (3-6 months); large *es* - **(0) -** Concurrent: paternal interactive behaviours (i.e., sensitivity) during father-infant interactions → infant subcortical grey matter volumes (3-6 months); *ns*   **Convergent validity (with a measure of maternal parenting):**   - **(1) -** Paternal interactive behaviours (i.e., sensitivity) → maternal sensitivity (3-6 months); medium to large *es* | | |
|  |  |  |  |  |  | | |
| **National Institute of Child Health and Human Development coding scales** **(NICHD; Early Child Care Research Network, 1999)**  ---------------------------------  *Initial target population*  Tool initially developed in a community sample of mothers and their infants observed at 6-months to 3-years (NICHD Early Child Care Research Network, 1999)  *Training and availability:*  -Training manual  -Self-guided training | **Brown & Cox (2019)** | *Inter-rater reliability*   - On a sub-sample of 20%, ICC = 0.87 - 0.90, across father domains   *Inter-scale item / domain correlations*: n/r  *Factor analysis*: n/r  *Test-retest reliability*: n/r | N/A | N/A | **Convergent validity (another measure of paternal parenting):**   - **(1) -** Paternal interactive behaviours (i.e., sensitivity) → fathers perceptions of their pleasure in parenting; medium *es*   **Criterion validity (offspring outcomes):**   - **(0) –** Concurrent: Paternal interactive behaviours (i.e., sensitivity) infant attachment security (12-months); *ns* | | |
|  |  |  |  |  |  | | |
|  | **Mills-Koonce et al., (2015)** | *Inter-rater reliability*   - On a sub-sample of 30%, *r* = > 0.80 across father domains   *Inter-scale item / domain correlations*: n/r  *Factor analysis*: n/r  *Test-retest reliability*:   - Paternal sensitive parenting 6-months → 24-months (*r* = 0.42, *p* = < .001) | N/A | N/A | **Convergent validity (with a measure of maternal parenting):**   - **(1) -** Paternal interactive behaviours (i.e., overall sensitive parenting) → maternal interactive behaviours (same sub-scale) at 6-months; medium *es*   **Criterion validity (offspring outcomes):**   - **(0) -** Predictive: paternal interactive behaviours (i.e., sensitive parenting) during father-infant interactions (6-months) → child cognitive development (6, 15-months); *ns* (direct effects extracted) - **(0) -** Predictive: paternal interactive behaviours (i.e., sensitive parenting) during father-infant interactions (24-months) → child executive functioning (3-years); *ns* (direct effects extracted) | | |
|  |  |  |  |  |  | | |
|  | **Hall et al. (2014)** | *Inter-rater reliability*   - On a sub-sample of 20%, ICC = 0.77 - 0.90, across father domains   *Inter-scale item / domain correlations*: n/r  *Factor analysis*: n/r  *Test-retest reliability*: n/r | N/A | N/A | *No reported evidence of convergent, discriminant or criterion validity related to the NICHD, from this study* | | |
|  |  |  |  |  |  | | |
|  | **Tamis-LeMonda et al., (2004)** | *Inter-rater reliability*   - On a sub-sample of 15%, IRR range from 84-100%, across parent domains   *Inter-scale item / domain correlations:* n/r  *Factor analysis:* n/r  *Test-retest reliability:* | N/A | N/A | **Criterion validity (offspring outcomes):**   - **(1) -** Concurrent: paternal interactive behaviours (i.e., sensitivity, cognitive stimulation, intrusiveness and paternal supportive behaviours (composite)) and paternal affect (i.e., positive regard) during father-infant interactions → infant MDI scores (24-months); small and medium *es* - **(0) -** Concurrent: paternal interactive behaviours (i.e., detachment) and paternal affect (i.e., negative regard) during father-infant interactions → infant MDI scores (24-months); *ns* - **(1) –** Predictive: paternal interactive behaviours (i.e., sensitivity, cognitive stimulation, detachment, intrusiveness, paternal supportive behaviours (composite)) and paternal affect (i.e., positive regard) during father-infant interactions → infant MDI scores (36-months); small and medium *es* - **(1) –** Predictive: paternal interactive behaviours (i.e., sensitivity, cognitive stimulation, paternal supportive behaviours (composite)) and paternal affect (i.e., positive regard) during father-infant interactions → infant PPVT scores (36-months); small and medium *es* - **(0) –** Predictive: paternal interactive behaviours (i.e., detachment, intrusiveness) and paternal affect (i.e., negative regard) during father-infant interactions → infant PPVT scores (36-months); *ns*   **Convergent validity (with a measure of maternal parenting):**   - **(1) -** Paternal interactive behaviours (i.e., sensitivity, cognitive stimulation, intrusiveness) and affect (i.e. positive and negative regard) → maternal interactive behaviours and maternal affect (same sub-scales); small to medium *es* | | |
|  |  |  |  |  |  | | |
|  | **Towe-Goodman et al., (2014)** | *Inter-rater reliability*   - On a sub-sample of 20%, ICC = 0.85 (7-months) and 0.91 (24-months) for overall paternal sensitive parenting   *Inter-scale item / domain correlations: n/r*  *Factor analysis:* n/r  *Test-retest reliability:*   - Paternal sensitive parenting 7-months → 24-months (*r* = 0.44, *p* = 0.01) | N/A | N/A | **Convergent validity (with a measure of maternal parenting):**   - **(1) -** Paternal interactive behaviours (i.e., overall sensitive parenting) → maternal interactive behaviours (same sub-scale) at 7- and 24-months; medium *es’s*   **Criterion validity (offspring outcomes):**   - **(1) -** Predictive: paternal interactive behaviours (i.e., sensitive parenting) during father-infant interactions (24-months) → child executive functioning (3-years); large *es* - **(0) -** Predictive: paternal interactive behaviours (i.e., sensitive parenting) during father-infant interactions (7-months) → child executive functioning (3-years); *ns* | | |
|  |  |  |  |  |  | | |
|  | **Volling et al., (2019)** | *Inter-rater reliability*   - On a sub-sample of 15%, IRR range from 0.79 – 0.89. across father domains   *Inter-scale item / domain correlations:*   - Paternal sensitivity → cognitive stimulation (*r* = .51, *p* = <.01) and positive reward (*r* = .77, *p* = <. 01) - Paternal sensitivity → detachment (*r* = -.61, *p* < .01) and intrusiveness (*r* = -.37, *p* = <.01)   *Factor analysis:* n/r  *Test-retest reliability:* n/r | N/A | N/A | **Convergent validity (with a measure of maternal parenting):**   - **(1) -** Paternal interactive behaviours (i.e., sensitivity, cognitive stimulation, intrusiveness) and affect (i.e. positive regard) → maternal interactive behaviours and maternal affect (same sub-scales); small *es*   **Criterion validity (offspring outcomes):**   - **(0) -** Concurrent: paternal interactive behaviours (i.e., sensitivity, cognitive stimulation, intrusiveness, detachment) and paternal affect (i.e., positive regard) during father-infant interactions → infant attachment security (12-months); *ns* | | |
|  |  |  |  |  |  | | |
| **Scale for Mother-Infant Interactions during Feeding** **(SVIA; Chatoor et al., 1997)**  ---------------------------------  *Initial target population*  Tool initially developed in a sample of mothers and their infants observed at 1-month to 3 years (Chatoor et al., 1997)  *Training and availability*   - Publication describing tool (SVIA) development (Chatoor et al., 1997) - Self-guided training | **Cerniglia et al. (2014)** | *Inter-rater reliability* n/r  *Inter-scale item / domain correlations:* n/r  *Factor analysis:* n/r  *Test-retest reliability:* n/r | N/A | N/A | **Convergent validity (severity of psychological symptoms):**   - **(0) -** Paternal psychological symptoms: → paternal interactive behaviours (i.e., interactional conflict) and affective state (24-months); *ns* - **(0) -** Paternal psychological symptom**:** → infant interactive behaviours (i.e., food refusal behaviours) (24-months); *ns* - **(0) -** Paternal psychological symptoms**:** → dyadic affective state (24-months); *ns* | | |
|  |  |  |  |  |  |  | |
|  | **Tambelli et al. (2015)** | *Inter-rater reliability*   - *r* = 0.74 - 0.89, across all parent domains (sub-sample, n/r)   *Note: reliability reported across mothers and fathers, not specifically fathers*  *Inter-scale item / domain correlations:* n/r  *Factor analysis:* n/r  *Test-retest reliability:* n/r | N/A | N/A | **Convergent validity (severity of psychological symptoms):**   - **(0) -** Paternal psychological symptoms: → paternal interactive behaviour (i.e., interactional conflict) and affect (i.e., overall affective state) during father-infant feeding interactions (3-months); *ns* - **(1) -** Paternal anxiety symptoms: → infant interactive behaviours (i.e., food refusal behaviours) (3-months); medium *es* - **(0) -** Paternal psychological symptoms: → dyadic affective state (3-months); *ns* | | |
| **Observational tools developed in paternal (*n* = 2) and parental samples (*n* = 5)** | | | | | | | |
|  |  |  |  |  |  | | |
| **Emotional Availability Scales (EAS; Biringen, 2008)**  ----------------------------------  *Initial target population*  Tool developed in a sample of parents and their young children  *Training and availability:*   - Unpublished training manual - Completion of training course required (face-to-face, group training or self-paced distance learning) | **Rossen et al. (2018)** | *Inter-rater reliability*   - On a sub-sample of *n* = 23 father-infant interactions, ICC = 0.85 for the overall measure, and **ϰ** = 0.64 – 0.83 across paternal domains - On a sub-sample of *n* = 23 father-infant interactions, **ϰ** = 0.60 – 0.74 across infant domains   *Inter-scale item / domain correlations:* n/r  *Factor analysis:* n/r  *Test-retest reliability:* n/r | *Content validity*  Reviewed from source (Biringen, 2008):  *Theory*: n/r  *Expert review*: n/r  *Content analysis with target group*: n/r | *Response process*  Reviewed from source (Biringen, 2008): n/r  Reviewed from source (Rossen et al. 2018): n/r | *No reported evidence of convergent, discriminant or criterion validity related to the EAS, from this study* | | |
|  |  |  |  |  |  | | |
| **Nursing Child Assessment Teaching Scales (NCATS; Sumner & Spietz, 1994)**  ---------------------------------  *Initial target population*  Tool initially developed in a sample of parents – targeted for use from birth to 3-years (Sumner & Spietz, 1994)  *Training and availability*   - Published training manual - Training course required with certificate of reliability | **Goodman (2008)** | *Cronbach alpha:*   - α = 0.74, for the overall measure in fathers (sub-sample, n/r)   *Inter-scale item / domain correlations:* n/r  *Factor analysis:* n/r  *Test-retest reliability:* n/r | *Content validity*  Reviewed from Goodman et al. (2008):  *Theory:* n/r  *Expert review*: n/r  *Content analysis with target group*: n/r | *Response process*   - Reviewed from Goodman et al. (2008): n/r | **Convergent validity (severity of psychological symptoms)**   - **(1) -** Paternal depressive symptoms**:** → overall quality of father-infant interactions (2-3 months); small *es* - **(1) -** Maternal depressive symptoms**:** → overall quality of father-infant interactions (2-3 months); small to medium *es*   **Discriminant validity (parent symptom level groups):**   - **(1) -** Maternal depressive symptom level groups**:** higher (EPDS > 10) *vs.* lower (EPDS < 10) symptoms →   lower overall scores (i.e., less optimal) during father-infant interactions (2-3 months) | | |
|  |  |  |  |  |  | | |
|  | **Magill-Evans & Harrison (1999)** | *Inter-rater reliability*   - On a sub-sample of 10%, 86% IRR average across paternal domains   *Inter-scale item / domain correlations:* n/r  *Factor analysis:* n/r  *Test-retest reliability:* n/r | *Content validity*  Reviewed from Magill-Evans & Harrison (1999): n/r  *Theory:* n/r  *Expert review*: n/r  *Content analysis with target group*: n/r | *Response process*  Reviewed from Magill-Evans & Harrison (1999): n/r | **Criterion validity (offspring outcomes):**   - **(1) –** Predictive**:** quality of father-infant interactions (3-months; total paternal NCATS score) → child receptive language (18-months); small *es* - **(0) –** Predictive**:** quality of father-infant interactions (12-months; total paternal NCATS score) → child receptive language (18-months); *ns* - **(0) –** Predictive**:** quality of father-infant interactions (3, 12-months; total paternal NCATS score) → child Bayley Mental Development scale and expressive language development (18-months); *ns* | | |
|  |  |  |  |  |  | | |
| **Parent Child Early Relational Assessment Scale (PCERA; Clark, 1985, 1999)**  ----------------------------------  *Initial target population*  Tool initially developed in sample of parents observed with their young infants (Clark, 1985)  *Training and availability*   - Unpublished training manual - Training required | **Edhborg et al. (2003)** | *Cronbach alpha*   - α = 0.86 – 0.88, for paternal domains across structured and free-play interactions with infants (sub-sample, n/r) - α = 0.76 – 0.94, for infant domains across structured and free-play interactions with fathers (sub-sample, n/r)   *Inter-scale item / domain correlations:* n/r  *Factor analysis:* n/r  *Test-retest reliability:* n/r | *Content validity*   - Reviewed from Edhborg et al. (2003):   *Theory:* n/r  *Expert review:* n/r  *Content analysis with target group*: n/r | *Response process*   - Reviewed from Edhborg et al. (2003): n/r | **Discriminant validity (parent symptom level groups):**   - **(1)** Maternal depressive symptom level groups**:** higher (EPDS > 12) *vs.* lower (EPDS < 10) symptoms (2-months) → increased levels of paternal enjoyment and pleasure during *father-infant structured play* interactions (15-18 months) - **(0)** Maternal symptom level groups**:** → overall paternal positive interactive behaviours during *father-infant free-play* interactions; *ns* - **(1)** Maternal depressive symptom level groups: higher *vs.* lower symptoms → increased levels of infant interactive affect (i.e., negative affect) during *father-infant structured play* interactions (15-18 months) - **(0)** Maternal depressive symptom level groups**:** → overall infant interactive mood during *father-infant free play* interactions (15-18 months); *ns* - **(0)** Maternal depressive symptom level groups**:** → overall infant interactive behaviours during father-infant *free play* or *structured play* interactions (15-18 months); *ns* | | |
|  |  |  |  |  |  | | |
|  | **Eiden et al. (2009)** | *Inter-rater reliability*   - On a sub-sample of 17%, IRR = 0.81 – 0.92, across parent domains   *Note:* reliability reported across mothers and fathers, not specifically fathers  *Inter-scale item / domain correlations:*   - Within-scale correlations between paternal sensitivity and warmth   *(r* = 0.85. *p* < .05), and sensitivity and negative affect (*r* = 0.81, *p* < .05)  *Factor analysis:*   - One paternal factor identified (composite of individual sub-scales)   *Test-retest reliability:* n/r | *Content validity*   - Reviewed from Eiden et al. (2009):   *Theory:* n/r  *Expert review:* n/r  *Content analysis with target group*: n/r | *Response process*   - Reviewed from Eiden et al. (2009): *n/r* | **Convergent validity (with a measure of maternal parenting):**   - **(1) -** Paternal interactive behaviours (i.e., sensitivity, warmth) and affect (i.e. negative affect) → maternal interactive behaviours and maternal affect (same sub-scales – 24-months); medium to large *es’s*   **Convergent validity (severity of psychological symptoms):**   - **(1) -** Paternal depressive symptoms (12-18 months): → paternal interactive behaviours (i.e., warmth) (24-months); small *es* - **(0) -** Paternal depressive symptoms (12-18 months): → paternal interactive behaviours (i.e., sensitivity) and affect (i.e., negative affect) (24-months); *ns* - **(1) -** Maternal depressive symptoms (12-18 months): → paternal interactive behaviours (i.e., sensitivity, warmth) and affect (i.e., negative affect) (24-months); small *es*   **Discriminant validity (parent clinical diagnostic groups):**   - **(1) -** Paternal clinical diagnostic groups: presence *vs.* absence of alcohol dependence (12-18 months) → lower levels of paternal sensitivity/warmth (24-months); medium *es*   **Criterion validity (offspring outcomes):**   - **(0) –** Concurrent/predictive**:** paternal interactive behaviours (i.e., sensitivity, warmth) and affect (i.e., negative affect) (24-months) → child self-regulation and externalising problems (24, 36-months); *ns* | | |
|  |  |  |  |  |  | | |
| **Parent Child Early Relational Assessment Scale (PCERA; Clark, 1980)** – *unpublished version* | **Eiden, Chavez & Leonard (1999)** | *Inter-rater reliability*   - *r* = 0.89 – 0.95 across parent domains (not specifically father domains)   *Inter-scale item / domain correlations:* n/r  *Factor analysis:*   - Three parental and four infant composite parenting scales were based on factor analyses   *Test-retest reliability:* n/r | *Content validity*  Reviewed from source (Eiden et al., 1999):  *Theory:* n/r  *Expert review*: n/r  *Content analysis with target group*: n/r | *Response process*  Reviewed from source (Eiden et al., 1999): n/r | **Convergent validity (severity of psychological symptoms)**   - **(1) -** Paternal depressive symptoms**:** → paternal interactive behaviours (i.e., sensitivity) (12-months); small *es* - **(1) -** Paternal alcohol abuse and dependence**:** → paternal interactive behaviours (i.e., sensitivity) (12-months); small – medium *es* - **(1) -** Paternal alcohol abuse and dependence**:** → paternal speech (i.e., verbalisations) (12-months); small – medium *es* - **(1) -** Paternal alcohol abuse and dependence**:** → paternal affect (i.e., positive and negative affect) (12-months); small – medium *es* - **(1) -** Paternal alcohol abuse and dependence**:** → infant interactive behaviours (i.e., responsiveness) (12-months); small *es* - **(0) -** Paternal depressive symptoms**:** → paternal speech (i.e., verbalisations), paternal and infant affect (i.e., positive and negative affect) and infant interactive behaviour (i.e., responsiveness) (12-months); *ns*   **Criterion validity (offspring outcomes):**   - **(0) -** Concurrent**:** paternal interactive behaviours, paternal and infant affect and infant interactive behaviours → infant fussy-difficult temperament (12-months); *ns* | | |
|  |  |  |  |  |  | | |
| **Paternal Cognitive Attributional Mentalizing Scale (PCAMS; Sethna et al. 2012)**  -----------------------------------  *Initial target population*  Tool initially developed in a community sample of fathers with diagnosed depression and a group of non-depressed fathers, and their infants observed at 3-months  *Training and availability*  -Training manual available  upon request  -Self-guided training | **Sethna et al. (2012)** | *Inter-rater reliability:*   - On a sub-sample of 25%, ICC = 0.60 – 0.98, across father domains   *Inter-scale item / domain correlations:* n/r  *Factor analysis:* n/r  *Test-retest reliability:* n/r | *Content validity*  Reviewed from source (Sethna et al., 2012):  *Theory:* evidence of theory driven items specific to paternal parenting  *Expert review*: n/r  *Content analysis with target group*: n/r | *Response process*  Reviewed from source (Sethna et al., 2012): n/r | **Discriminant validity (parent clinical diagnostic groups):**   - **(1) -** Paternal clinical diagnostic groups: presence *vs.* absence of depression → higher proportions of speech focused on the paternal experience, lower proportions of speech focused on the infants’ experience, and a higher proportion of infant-directed negativity (3-months) - **(0) -** Paternal clinical diagnostic groups: → paternal speech (i.e., mental state comments) (3-months); *ns* | | |
|  |  |  |  |  |  | | |
| **Paternal-Physicality, Affect & Touch Scale** **(P-PATS; Sethna et al. 2018)**  ---------------------------------  *Initial target population*  Tool initially developed in a community sample of fathers with diagnosed depression and a group of non-depressed fathers, and their infants observed at 3-months  *Training and availability:*   - Training manual available upon request - Self-guided training | **Sethna et al. (2018)** | *Inter-rater reliability*     - ICC = 0.74 – 0.97 across paternal domains (all interactions were double coded)   *Inter-scale item / domain correlations:* n/r  *Factor analysis:* n/r  *Test-retest reliability:* n/r | *Content validity*  Reviewed from source (Sethna et al., 2018):   - *Theory:* evidence of theory driven items specific to paternal parenting   *Expert review*: n/r  *Content analysis with target group*: n/r | *Response process*  Reviewed from source (Sethna et al., 2018): n/r | **Discriminant validity (parent clinical diagnostic groups):**   - **(1) -** Paternal clinical diagnostic groups*:* presence *vs.* absence of depression → lower levels of paternal interactive behaviours (i.e., episodes of playful excitation, active engagement, time spent in paternal gentle touch) (3-months) - **(0) -** Paternal clinical diagnostic groups*:* → paternal interactive behaviours (i.e., physicality in play overall duration of paternal touch (quantity) and time spent in vigorous touch (quality)) (3-months); *ns* | | |
|  |  |  |  |  |  | | |
| **Unnamed (Feldman &** **Eidelman, 2007)**  -----------------------------------  *Initial target population*  Tool described in a published article including a sample of mothers and fathers, and their young infants observed at 3-months (Feldman & Eidelman, 2007)  *Training and availability:*   - Publication describing tool details (Feldman & Eidelman, 2007) - Self-guided training | **Feldman & Eidelman (2007)** | *Inter-rater reliability*   - On a sub-sample of   *n* = 20, **ϰ** = 0.75 - 0.87 across all sub-domains and **ϰ** = 0.80 for the overall measure  *Note:* reliability reported across mothers and fathers, not specifically fathers   - *Inter-scale item / domain correlations:* father-infant synchrony → father affectionate touch; *ns*   *Factor analysis:* n/r  *Test-retest reliability:* n/r | *Content validity*  Reviewed from source (Feldman & Eidelman, 2007):   - *Theory:* evidence of theory driven items related to paternal parenting – specifically, the inclusion of father-infant synchrony and affectionate touch   *Expert review*: n/r  *Content analysis with target group*: n/r | *Response process*  Reviewed from source (Feldman & Eidelman, 2007): n/r | **Convergent validity (with a measure of maternal parenting):**   - **(1) –** Father-infant synchrony: → mother-infant synchrony (3-months); medium *es* - **(1) –** Father affectionate touch: → mother affectionate touch (3-months); medium *es* | | |
|  |  |  |  |  |  |  | |
| **Unnamed tool (Pepi, 1981)**^a^  ----------------------------------  *Initial target population*  Tool initially developed in a community sample of mothers and fathers, and their infants observed during the first 3-months (Pepi, 1981)  *Training and availability*   - Coding scheme developed as part of an unpublished dissertation (Pepi (1981) - Self-guided training | **Beal, 1989** | *Inter-rater reliability*   - ICC = 0.83 – 0.95 across paternal sub-domains (sub-sample, n/r) - ICC = 0.74 – 0.92 across infant sub-domains (sub-sample, n/r)   *Inter-scale item / domain correlations:* n/r  *Factor analysis:* n/r  *Test-retest reliability:* n/r | *Content validity*   - Reviewed from Beal (1989):   *Theory*: n/r  *Expert review*: n/r  *Content analysis with target group*: n/r | *Response process*   - Reviewed from Beal (1989): n/r | **Criterion validity (offspring outcomes):**   - **(0) -** Concurrent**:** overall quality of paternal interactive behaviours → infant difficult temperament (8-weeks); *ns* | | |
|  | | | | | | | |
| 1 = significant association (*p* < 0.05), 0 = non-significant association (*p* > 0.05); n/r = not reported; *es* = effect size; ns = non-significant  ^a^ Details of initial target population and content validity reviewed and extracted from initial source of tool (where available) or the related publication(s)  ^b^ Internal structure and relations with other variables extracted from related publication(s)  ^c^ Response process reviewed and extracted from initial source of tool (where available) and/or the related publication(s) | | | | | | | |

.
