# Supplementary material for: The Applicability and Performance of Tools Used to Assess the Father-Offspring Relationship in Relation to Parental Psychopathology and Offspring Outcomes
Source: Front Psychiatry. 2021 Jan 5;11:596857. doi: 10.3389/fpsyt.2020.596857 (PMC7814871; doi:10.3389/fpsyt.2020.596857)
Supplement: Supplementary file 8 [file Table_8.docx]

| **Supplementary Materials_Table 8**  Validity evidence supporting self-report tools (*n* = 18) used to assess the father-offspring relationship – including relationship quality and father involvement | | | | | | | |
| --- | --- | --- | --- | --- | --- | --- | --- |
| **Self-report tool / Target population^a^ / Training** | **Related Publication(s)** | **Internal Structure^b^** | **Content Validity^a^** | **Response Process^c^** | **Relations with other variables^b^**  Convergent, Discriminant, Criterion Validity  *(1) = significant association; (0) = non-significant association* | | |
|  |  |  |  |  |  | | |
| **Self-report tools developed in maternal samples (*n* =4)** | | | | | | | |
|  |  |  |  |  |  | | |
|  | | | | | | |  |
| **Child Rearing Questionnaire (CRQ; Paterson & Sanson, 1999)**  ----------------------------------  *Initial target population*  Tool initially developed in a sample of mainly mothers reporting on their pre-school children  *Training and availability:*   - Published article describing the tool (Paterson & Sandon, 1999) - Self-guided training | **Giallo et al. (2015)** | *Internal consistency*   - *Cronbach alpha:* α = 0.83, for the overall measure in fathers   *Test-retest reliability:*   - Paternal warmth at 3-12 months → 2-3 years *(r* = 0.51, *p* < .001)   *Inter-scale item / domain correlations:* n/r  *Factor analysis:* n/r | N/A | N/A | **Convergent validity (severity of psychological symptoms):**   - **(0) -** Paternal symptoms of psychological distress: → paternal warmth (3-12 months); *ns* | | |
|  |  |  |  |  |  | | |
| **How I Feel About my Baby Now (FAB, Leifers, 1977)**  -----------------------------------  *Initial target population*:  Tool developed in a community sample of women completing self-reports during pregnancy and at 2 and 7-months  *Training and availability:*   - Published article describing the tool (Leifers, 1977) - Self-guided training | **Ferketich & Mercer (1995)** | *Internal consistency*   - *Cronbach alpha:* α = 0.66 – 0.81, range in reliability scores for the overall measure in fathers   *Inter-scale item / domain correlations:* n/r  *Factor analysis:* n/r  *Test-retest reliability:*  n/r | N/A | N/A | **Convergent validity (another measure of paternal parenting):**   - **(1) -** Fathers attachment to the infant (FAB) → paternal competence (*via* the Paternal Sense of Competence scale; Wallston & Wandersman, 1978) (birth and 8-months only; first-time fathers only); medium *es* - **(0) -** Fathers attachment to the infant (FAB) → paternal competence (Paternal Sense of Competence scale; Wallston & Wandersman, 1978) (birth, 1, 4 and 8-months; multiparous fathers); *ns*   **Convergent validity (severity of psychological symptoms):**   - **(1) -** Paternal depressive symptoms*:* → fathers’ attachment to the foetus (1 and 4-months; first-time fathers); small to medium *es* - **(0) -** Paternal depressive symptoms*:* → fathers’ attachment to the foetus (birth and 8-months; first-time fathers); *ns* - **(0)** Paternal anxiety symptoms*:* → fathers’ attachment to the infant (birth, 1, 4 and 8-months; first-time fathers); *ns* - **(1) -** Paternal depressive symptoms*:* → fathers’ attachment to the infant (birth and 8-months; multiparous fathers); medium *es* - **(0) -** Paternal depressive symptoms*:* → fathers’ attachment to the foetus (1 and 4-months; multiparous fathers); *ns* - **(1) -** Paternal anxiety symptoms: → fathers’ attachment to the infant (1 and 4-months; multiparous fathers); medium *es* - **(0) -** Paternal anxiety symptoms: → fathers’ attachment to the infant (birth and 8-months; multiparous fathers); *ns* | | |
|  |  |  |  |  |  | | |
| **Parental Attachment Questionnaire (PAQ; Condon & Corkindale, 1998)**  ------------------------------  *Initial target population*  Tool initially developed in a sample of mothers reporting during the antenatal period and first year postnatal  *Training and availability:*   - Published article describing the tool (Condon & Corkindale, 1998)   -Self-guided training | **Wynter et al. (2016)** | *Internal consistency:* n/r  *Inter-scale item / domain correlations:* n/r  *Factor analysis:* n/r  *Test-retest reliability:*  n/r | N/A | N/A | **Convergent validity (severity of psychological symptoms):**   - **(1) -** Paternal depressive symptoms: → fathers’ attachment to the infant (6-months); small to medium *es* | | |
|  |  |  |  |  |  | | |
| **Postpartum Bonding Questionnaire (PBQ; Brockington et al., 2001)**  ------------------------------  *Initial target population*  Tool initially developed in a sample of mothers reporting on their young infants  *Training and availability:*   - Tool freely available in published article describing the tools development (Brockington et al., 2001)   -Self-guided training | **Edhborg et al. (2005)** | *Test-retest reliability*   - Father-infant bonding difficulties (total scores) at 1-week → 8-weeks *(no difference in scores at the two time-points)*   *Inter-scale item / domain correlations:*   - Within-scale associations between total scores in bonding difficulties and impaired bonding sub-scale (*ß* = .55, *p* = .016) and rejection and anger sub-scale (*ß* = .56, *p* = .09; *trend level*)   *Internal consistency:* n/r  *Factor analysis:* n/r | N/A | N/A | **Criterion validity (offspring outcomes):**   - **(1) –** Concurrent: father-infant bonding difficulties → infant dull and unpredictable temperament (2-months); large *es* - **(0) -** Concurrent: father-infant bonding difficulties → infant fussy-difficult infant temperament (2-months); *ns*   **Convergent validity (severity of psychological symptoms):**   - **(1) -** Paternal depressive symptoms: → father-infant bonding difficulties (2-months; large *es* - **(0) -** Maternal depressive symptoms: → father-infant bonding difficulties (2-months); *ns* | | |
|  |  |  |  |  |  | | |
|  | **Kerstis et al., (2015)** | *Internal consistency:* n/r  *Inter-scale item / domain correlations:* n/r  *Factor analysis:* n/r  *Test-retest reliability:*  n/r | N/A | N/A | **Convergent validity (severity of psychological symptoms):**   - **(1) -** Paternal depressive symptoms: → fathers impaired bonding with their infant (6-weeks and 6-months); small *es* - **(1) -** Maternal depressive symptoms: → fathers impaired bonding with their infant (6-weeks and 6-months); small *es*   **Convergent validity (with a measure of maternal parenting):**   - **(1) -** Fathers impaired bonding with their infant → mothers impaired bonding with their infant (via the same measure; PBQ) (6-months); small *es* | | |
| **Self-report tools developed in parental (*n* = 9) and paternal samples (*n* = 5)** | | | | | | | |
|  | | | | | | | |
| **Childrearing**  **Practices Report (CRPR; Block, 1965)**  ----------------------------------  *Initial target population*  Tool initially developed in a sample of parents and their young toddlers and older children  *Training and availability:*   - Published article describing the tool (Block, 1965) | **Mezulis, Hyde & Clark (2004)** | *Internal consistency:*   - Good internal consistency across paternal warmth and moderate consistency across paternal control (statistics n/r)   *Test-retest reliability:*   - Paternal warmth at 12-months → 4.5 years *(r* = 0.50, *p* < .01) - Paternal control at 12-months → 4.5 years *(r* = 0.56, *p* < .01)   *Inter-scale item / domain correlations:*   - Within-scale correlations between paternal warmth and control (*r* = -0.03; *ns*)   *Factor analysis:*   - 2 factors identified – paternal warmth and control | *Content validity*  Reviewed from Mezulis, Hyde & Clark (2004): n/r   - *Theory:* n/r - *Expert review:* n/r - *Content analysis with target group:* n/r | *Response process*  Reviewed from Mezulis, Hyde & Clark (2004): n/r | **Convergent validity (severity of psychological symptoms):**   - **(1) -** Paternal depressive symptoms: → paternal warmth and control (12-months); small *es* - **(0) -** Maternal depressive symptoms: → paternal warmth and control (12-months); *ns*   **Discriminant validity (parent clinical diagnostic groups):**  **(0) -** Maternal clinical diagnostic groups**:** presence *vs.* absence depression → paternal warmth and control (12-months); *ns*  **Convergent validity (another measure of paternal parenting):**   - **(0) –** Paternal warmth and control → quantity of paternal involvement (*via* self-reported hours) (12-months); *ns*   **Criterion validity (offspring outcomes):**   - **(1) –** Predictive: paternal warmth (12-months) → child internalizing problems (pre-school); small *es* - **(0) –** Predictive: paternal control (12-months) → child externalizing problems (pre-school); *ns* | | |
|  |  |  |  |  |  | | |
| **Korean Paternal-Fetal Attachment Scale (K-PAFAS; Noh & Yeom, 2017)**  -----------------------------------  *Initial target population*  Tool developed in a community sample of expectant fathers reporting on their attachment to their foetus  *Training and availability:*   - Published article describing development of the tool (Noh & Yeom, 2017)   -Self-guided training | **Noh & Yeom, (2017)** | *Internal consistency*   - *Cronbach alpha:*   α = 0.89, for the overall measure in fathers  α = 0.56–0.86, across all sub-domains in fathers  *Factor analysis*   - 4 factors identified - *Inter-scale item / domain correlations:* within-scale correlations between sub-domains and total scores in father-foetal attachment *(r* = 0.41 - 0.70)   *Test-retest reliability*   - Father-foetal attachment over a 2-week period (*r* = 0.67 – 0.94, *p* < 0.001; range across sub-domains) | *Content validity*  Reviewed from source (Koh & Yeom, 2017)   - *Theory:* evidence of theory driven items specific to the measurement of paternal parenting - *Expert review:* items reviewed by experts who rated the relevance of each item - *Content analysis with target group:*   items developed through interviews with 10 expectant fathers and subject to content analysis | *Response process*  Reviewed from source (Koh & Yeom, 2017)  *Feedback:* pilot testing on a sample of fathers who rated the clarity and understanding of items. | **Convergent validity (another measure of paternal parenting):**   - **(1) -** fathers attachment to the foetus (K-PAFAS) → another measure of paternal antenatal attachment (via the Paternal Antenatal Attachment Scale (PAAS; Condon, 1993)); large *es*   **Convergent validity (severity of psychological symptoms):**   - **(1) -** Paternal depressive symptoms**:** → fathers’ attachment to the foetus (antenatally); small *es* | | |
|  |  |  |  |  |  | | |
| **Home Observation for Measurement of the**  **Environment**  **(HOME) -Short Form version (Baker & Mott, 1992)**  -----------------------------------  *Initial target population*  Tool developed in a sample of parents  *Training and availability:*   - Article describing the tool (Baker & Mott, 1992)   -Self-guided training | **Bronte-Tinkew et al., (2007)** | *Internal consistency*   - *Cronbach alpha:*   α = 0.84, for the overall measure in fathers  *Factor analysis*: n/r  *Inter-scale item / domain correlations:* n/r  *Test-retest reliability:* n/r | *Content validity*~~:~~   - Reviewed from Bronte-Tinkew et al., (2007)   *Theory:* n/r  *Expert review:* n/r  *Content analysis with target group:* n/r | *Response process*   - Reviewed from Bronte-Tinkew et al., (2007): n/r | **Convergent validity (severity of psychological symptoms):**   - **(1) -** Paternal depressive symptoms**:** → father involvement in child care related activities - **(0) -** Maternal depressive symptoms**:** → father involvement in childcare related activities; *ns* | | |
|  |  |  |  |  |  | | |
| **Parental Involvement Questionnaire (****Tikotzky et al., 2010)**  -----------------------------------  *Initial target population*  Tool developed in mothers and fathers reporting on their 1-6-month-old infants  *Training and availability:*   - Published article describing development of the tool (Tikotzky et al., 2010)   -Self-guided training | **Tikotzky et al. (2010)** | *Internal consistency*   - *Cronbach alpha,* α = 0.80, for the overall measure in fathers   *Test re-test reliability:*   - Parental combined involvement scores at 1-month → 6-months (*p* < 0.05)   *Inter-scale item / domain correlations:* n/r  *Factor analysis:* n/r | *Content validity*~~:~~   - Reviewed from source (Tikotzky et al., 2010): - *Theory:* n/r   *Expert review:* n/r  *Content analysis with target group:* n/r | *Response process*   - Reviewed from source (Tikotzky et al., 2010): n/r | **Criterion validity (offspring outcomes):**   - **(1) –** Predictive: parental combined involvement in childcare activities involvement (i.e., increased scores indicate higher paternal involvement) (1-month) → more consolidated infant sleep (6-months); medium *es*   **Convergent validity (with a measure of maternal parenting):**   - **(1) -** Fathers involvement in child care activities → mothers involvement in child care activities (via the same measure; Parental Involvement Questionnaire); large *es* | | |
|  |  | | | | | | |
|  | **Tikotzky et al. (2015)** | *Internal consistency*   - *Cronbach alpha,* α = 0.80, for the overall measure   *Test re-test reliability:*   - Parental combined involvement scores at 3-months → 6-months (*p* < 0.05)   *Inter-scale item / domain correlations:* n/r  *Factor analysis:* n/r | ↑ | ↑ | **Criterion validity (offspring outcomes):**   - **(1) –** Predictive: parental combined involvement in childcare activities involvement (i.e., increased scores indicate higher paternal involvement) (3-months) → more consolidated infant sleep (6-months); medium *es*   **Convergent validity (with a measure of maternal parenting):**   - **(1) -** Fathers involvement in child care activities → mothers involvement in child care activities (via the same measure; Parental Involvement Questionnaire); large *es* | | |
|  |  |  |  |  |  | | |
| **Paternal Antenatal Attachment Scale (PAAS; Condon et al., 1993)**  ------------------------------  *Initial target population*  Tool initially developed in a community sample of fathers reporting on their attachment with their foetus  *Training and availability:*   - Published article describing the tools development (Condon et al., 1993) - Copies of the instrument available upon request - Self-guided training | **Beesley et al., (2019)** | *Internal consistency*  - *Cronbach alpha: α* =  0.71 for the overall  measure in fathers  *Inter-scale item / domain correlations:* n/r  *Factor analysis:* n/r  *Test-retest reliability:* n/r | *Content validity*  Reviewed from source (Condon et al., 1993):   - *Theory driven:* evidence of theory driven item pool specific to the measurement of paternal parenting - *Expert review:* evidence of item development supported by clinical expertise and experience - *Content analysis with target group:*   Evidence of item development supported with interviews of 15 expectant couples subject to content analysis | *Response process*  Reviewed from source (Condon et al., 1993):   - *Feedback:* expectant couples completed questionnaires with items modified based on pilot responses   Reviewed from Beesley et al. (2019): n/r | **Convergent validity (severity of psychological symptoms):**   - **(0) -** Paternal depressive symptoms (antenatally): → fathers’ attachment to the foetus; *ns* - **(0) -** Paternal anxiety symptoms (antenatally): → fathers’ attachment to the foetus; *ns* | | |
|  |  |  |  |  |  | | |
|  | **Brandão et al. (2019)** | *Internal consistency*   - *Cronbach alpha:* α = 0.81, for the overall measure in fathers   *Inter-scale item / domain correlations:* n/r  *Factor analysis:* n/r  *Test-retest reliability:* n/r | ↑ | ↑ | **Convergent validity (severity of psychological symptoms):**   - **(1) -** Paternal depressive symptoms (antenatally): → fathers’ attachment to the foetus; moderate *es* - **(1) -** Paternal anxiety symptoms (antenatally): → fathers’ attachment to the foetus; small *es* - **(1) -** Maternal depressive symptoms (antenatally): → fathers’ attachment to the foetus - **(1) -** Maternal anxiety symptoms (antenatally): → fathers’ attachment to the foetus | | |
|  |  |  |  |  |  | | |
|  | **Dayton et al., (2019)** | *Internal consistency*   - *Cronbach alpha:*   α = 0.64, for the overall measure in fathers  *Factor analysis:* n/r  *Inter-scale item / domain correlations:*   - Within-scale correlations between quality of attachment and time in attachment subscales; *ns*   *Test-retest reliability:* n/r | ↑ | ↑ | **Convergent validity (severity of psychological symptoms):**   - **(0) -** Paternal psychiatric distress symptoms (antenatally): → fathers’ attachment to the foetus; *ns* | | |
|  |  |  |  |  |  | | |
|  | **de Cock et al., (2017)** | *Internal consistency*   - *Cronbach alpha:*   α = 0.78, for the overall measure in fathers  *Factor analysis:* n/r  *Inter-scale item / domain correlations:* n/r  *Test-retest reliability:* n/r | ↑ | ↑ | **Convergent validity (another measure of paternal parenting):**   - **(1) -** Fathers attachment to the foetus (PAAS)→ fathers attachment to the infant; large *es*   **Convergent validity (with a measure of maternal parenting):**   - **(1) -** Fathers attachment to the foetus (PAAS)→ mothers attachment to the foetus (*via.* the Maternal Antenatal Attachment Scale (MAAS; Condon, 1993)); medium *es*   **Criterion validity (offspring outcomes):**   - **(0) –** Predictive: fathers’ attachment to the foetus → infant executive functioning problems (24-months); *ns* | | |
|  |  |  |  |  |  | | |
|  | **Pisoni et al. (2015)** | *Internal consistency:* n/r  *Inter-scale item / domain correlations:* n/r  *Factor analysis:* n/r  *Test-retest reliability:* n/r | ↑ | ↑ | **Convergent validity (with a measure of maternal parenting):**   - **(1) -** Fathers attachment to the foetus (PAAS)→ mothers attachment to the foetus (*via.* the MAAS); medium to large *es*   **Convergent validity (severity of psychological symptoms):**   - **(0) -** Maternal depressive symptoms (antenatally): → fathers’ attachment to the foetus (in parents at risk for pre-term delivery group); *trend level, p =* 0.095; medium *es* - **(0) -** Maternal depressive symptoms (antenatally): → fathers’ attachment to the foetus (in parents experiencing a physiologic pregnancy group); *ns* - **(0) -** Maternal anxiety symptoms (antenatally): → fathers’ attachment to the foetus (in both groups - parents at risk for pre-term delivery and experiencing a physiologic pregnancy); *ns* | | |
|  |  |  |  |  |  |  | |
|  | **Fijałkowska &**  **Bielawska- Batorowicz** | *Internal consistency:*   - *Cronbach alpha: α* = 0.94 for the overall measure in fathers (based on the Polish translation of the scale)   *Inter-scale item / domain correlations:* n/r  *Factor analysis:* n/r  *Test-retest reliability:* n/r | ↑ | ↑ | **Convergent validity (severity of psychological symptoms):**   - **(1) -** Paternal depressive symptoms (antenatally): → fathers’ overall attachment towards the foetus; medium *es*   **Convergent validity (another measure of paternal parenting):**   - **(1) –** fathers overall attachment to the foetus (PAAS): → fathers’ overall attachment towards the infant (PPAQ; Condon et al., 2008); large *es* - **(1) –** fathers overall attachment to the foetus (PAAS): → fathers’ non-verbal representation of parental attachment (via The Pictorial Representation of Attachment Measure (PRAM; Vreeswijk et al., 2009)); large *es* | | |
|  |  |  |  |  |  |  | |
|  | **Vreeswijk et al. (2014)** | *Inter-scale correlations*   - Within-scale correlations conducted between total father-foetus attachment scores and quality of interaction (*r* = 0.90, *p* < 0.01) and intensity of preoccupation (*r* = 0.89, *p* < 0.01)   *Internal consistency:* n/r  *Factor analysis:* n/r  *Test-retest reliability:* n/r | ↑ | ↑ | **Convergent validity (severity of psychological symptoms):**   - **(1) -** Paternal depressive symptoms (antenatally): → fathers’ quality of attachment towards the foetus (sub-scale); small *es* - **(0) -** Paternal depressive symptoms (antenatally): → fathers’ overall attachment to the foetus and intensity of preoccupation (sub-scale); *ns* - **(1) -** Paternal anxiety symptoms (antenatally): → fathers’ quality of attachment towards the foetus; small *es* - **(1) -** Paternal anxiety symptoms (antenatally): (trait symptoms only) → fathers’ overall attachment to the foetus; small *es* - **(0) -** Paternal anxiety symptoms (antenatally): (state symptoms only) → fathers’ overall attachment to the foetus; *ns* - **(0) -** Paternal anxiety symptoms (antenatally): → intensity of preoccupation (sub-scale); *ns* | | |
|  |  |  |  |  |  | | |
| **Paternal Fetal Attachment Scale (PFAS; Weaver & Cranley, 1983)**  -----------------------------------  Tool initially developed in a sample of expectant ­fathers­ reporting on their unborn child  *Training and availability:*   - Published article describing development of the tool (Weaver & Cranley, 1983)   -Self-guided training | **Seimyr et al. (2009)** | *-Internal consistency:*   - *Cronbach alpha:* α = 0.85, for the overall measure in fathers - *Factor analysis:* 5 factors identified (an alternative factor structure to Cranley’s initial sub-scales)   *Inter-scale item / domain correlations:* n/r  *Test-retest reliability:* n/r | *Content validity:*   - Reviewed from source (Weaver & Cranley, 1983) - *Theory:* evidence of theory driven items specific to the measurement of paternal parenting   *Expert review:* n/r  *Content analysis with target group:* n/r | *Response process:*   - Reviewed from source (Weaver & Cranley, 1983): n/r - Reviewed from Seimyr et al. (2009): n/r | **Convergent validity (with a measure of maternal parenting):**   - **(1) –** Fathers attachment to the foetus (PFAS) → mothers attachment to the foetus (*via.* the Maternal Fetal Attachment Scale (Cranley, 1981); medium *es*   **Discriminant validity (parent symptom level groups):**   - **(0) -** Maternal depressive symptom level groups (antenatally): higher *vs.* lower symptoms → fathers’ attachment to the foetus; *ns* | | |
|  |  |  |  |  |  | | |
| **Paternal Involvement Scale** **(****PI; Rustia & Abbott, 1993)**  -----------------------------------  *Initial target population*  Tool initially developed in a community sample of mothers and fathers reporting on their infants in the first year  *Training and availability:*   - Published article describing development of the tool (Rustia & Abbott, 1993)   -Self-guided training | **Shorey et al. (2018)** | *Internal consistency*   - ICC = 0.93, for the average measure across sub-scales   *Test-re-test reliability:*   - Paternal involvement scores at the day of partners hospital discharge → 3 → 6-months (*F* = 14.82, *p* < 0.001)   *Inter-scale item / domain correlations:* n/r  *Factor analysis:* n/r | *Content validity:*   - Reviewed from source (Rustia & Abbot, 1993) - *Theory:* evidence of theory driven items specific to the measurement of paternal parenting   *Expert review:* n/r  *Content analysis with target group:* n/r | *Response process:*   - Reviewed from source (Rustia & Abbot, 1993): n/r - Reviewed from Shorey et al. (2018): n/r | **Convergent validity (another measure of paternal parenting):**   - **(1)** *–* Paternal involvement in child care activities (PI) → paternal self-efficiency (*via* the Parenting Self-Efficiency Scale; Leerkes & Crockenberg, 2003) (*β* = 0.16, *p* = .003) (6-months); small *es* | | |
|  |  |  |  |  |  | | |
| **Paternal Postnatal Attachment Questionnaire (PPAQ; Condon et al., 2008)**  -----------------------------------  *Initial target population*  Tool initially developed in a sample of first-time fathers reporting on their 6 and 12-month old infants  *Training and availability:*   - Published article describing the tools development (Condon et al., 2008) - Copies of the instrument available upon request - Self-guided training | **Condon et al. (2008)** | *Internal consistency:*   - *Cronbach alpha:*   α = 0.80, for the overall measure in fathers  α = 0.70, for the average measure across sub-scales in fathers  *Test-re-test reliability:*   - Father-infant attachment (total scores) at 6-months →   → 12-months (*r* = 0.65 – 0.70, *p* < .001)  *Factor analysis:*   - 3 factors identified (45% variance)   *Inter-scale item / domain correlations:*   - Within-scale correlations between sub-scales (tolerance, pleasure, and pride) at 6-months and 12-months (*r* = 0.42 to 0.52, *p* < 0.001 across all correlations) | *-Theory:*  Evidence of theory driven item pool specific to the measurement of paternal parenting  *-Expert review:*  Evidence of item development supported by clinical expertise and experience   - *Content analysis with target group:*   Evidence of item development supported through interviews with 15 postnatal fathers -responses subject to content analysis | *Response process*   - *Feedback:* a sub-sample of fathers (*n* = 65) piloted the PPAQ - responses subject to statistical item analysis and items modified based on responses | **Convergent validity (severity of psychological symptoms)**   - **(1) -** Paternal depressive symptoms: → fathers’ attachment to the infant (6-months; small *es*) (12-months; medium *es*)   **Criterion validity (offspring outcomes):**   - **(1) –** Concurrent: fathers’ attachment to the infant → infant difficult temperament (6-months); medium *es* | | |
|  |  |  |  |  |  | | |
|  | **de Cock et al., (2017)** | *Internal consistency*   - *Cronbach alpha:*   α = 0.83, for the overall measure in fathers  *Factor analysis:* n/r  *Inter-scale item / domain correlations:* n/r  *Test-retest reliability:*   - Fathers attachment to the infant (6-month) → (24-months) (*r* = .72, *p* <. 001). | ↑ | ↑ | **Convergent validity (with a measure of maternal parenting):**   - **(1) -** Fathers attachment to the infant (PPAQ)→ mothers attachment to the infant (*via.* the MPAQ at 6 and 24-months; Condon, 1993); medium *es*   **Criterion validity (offspring outcomes):**   - **(0) –** Predictive: fathers’ attachment to the infant (6 and 24-months) → infant executive functioning problems (24-months); *ns* | | |
|  |  |  |  |  |  | | |
|  | **Fijałkowska &**  **Bielawska- Batorowicz** | *Internal consistency:*   - *Cronbach alpha: α* = 0.86 for the overall measure in fathers (based on a Chinese translation of the scale)   *Inter-scale item / domain correlations:* n/r  *Factor analysis:* n/r  *Test-retest reliability:* n/r | ↑ | ↑ | **Convergent validity (severity of psychological symptoms):**   - **(1) -** Paternal depressive symptoms (antenatally): → fathers’ overall attachment towards the infant; medium to large *es* | | |
|  |  |  |  |  |  | | |
|  | **Ip et al. (2018)** | *Internal consistency:*   - *Cronbach alpha: α* = 0.93 for the overall measure in fathers (based on the Polish translation of the scale)   *Inter-scale item / domain correlations:* n/r  *Factor analysis:* n/r  *Test-retest reliability:* n/r | ↑ | ↑ | **Convergent validity (severity of psychological symptoms):**   - **(1) -** Paternal depressive symptoms (6-weeks): → fathers patience and tolerance (moderate *es*), pleasure in interaction (small *es*), and affection and pride attachment sub-scales (medium *es*)   **Criterion validity (offspring outcomes):**   - **(1) –** Predictive: fathers’ patience and tolerance attachment sub-scale (6-weeks) → infant overall development (6-months); medium *es* - **(1) –** Predictive: fathers’ pleasure in interaction attachment sub-scale (6-weeks) → infant social overall development (6-months); medium *es* - **(1) –** Predictive: fathers’ affection and pride attachment sub-scale (6-weeks) → infant social overall development (6-months); medium *es* - **(0) –** Predictive: fathers’ patience and tolerance attachment sub-scale (6-weeks) → infant social development (6-months); *ns* | | |
|  |  |  |  |  |  | | |
| **Paternal Postnatal Attachment Questionnaire (PPAQ) - unpublished version (Condon & Corkindale, 1998)**  *Note*, at the time of publication, authors of the related articles (Buist et al., 2002 and Feldstein et al., 2004) describe the use of an updated unpublished version of the PAQ for fathers – the PPAQ. Since this time, a published version of the PPAQ is available (see Condon et al., 2008, above)  ----------------------------------  *Initial target population*  Tool initially developed in a sample of fathers – no further details (unpublished version of the PPAQ) | **Buist et al. (2003)** | *Internal consistency:* n/r  *Inter-scale item / domain correlations:* n/r  *Factor analysis:* n/r  *Test-retest reliability:*  n/r | *Content validity:*   - Reviewed from Buist et al. (1993): - *Theory driven:*  Evidence of a theoretical basis specific to the measurement of paternal parenting   *Theory:* n/r  *Expert review:* n/r  *Content analysis with target group:* n/r | *Response process:*   - Reviewed from Buist et al. (1993): n/r | **Discriminant validity (parent symptom level groups):**   - **(1) -** Paternal depressive symptom level groups: higher (EPDS > 10) *vs.* lower symptoms (EPDS < 10) → increased scores in paternal hostility in interaction (sub-scale) (1 and 4-months) - **(1) -** Paternal depressive symptom level groups: higher *vs.* lower symptoms → lower scores in the quality of father-infant attachment (sub-scale) (1 and 4-months) - **(0) -** Paternal depressive symptom groups: → paternal pleasure in interaction (sub-scale) (1 and 4-months); *ns* | | |
|  |  |  |  |  |  |  | |
|  | **Feldstein et al. (2004)** | *Internal consistency*  - *Cronbach alpha:*  α = 0.85, for the overall measure in fathers  α = 0.60 – 0.72, for the measure across sub-scales in fathers  *Inter-scale item / domain correlations:* n/r  *Factor analysis:* n/r  *Test-retest reliability:*  n/r | ↑ | ↑ | **Convergent validity (with a measure of maternal parenting):**   - **(1) -** Fathers pleasure in interaction (sub-scale) → mothers pleasure in interaction (*via.* the Maternal Postnatal Attachment Questionnaire (MPAQ; Condon & Corkindale, 1998)); medium *es* - **(0) -** Fathers overall attachment to the infant, quality of attachment and absence of hostility (sub-scales) → mothers overall attachment to the infant, quality of attachment and absence of hostility (*via.* the MPAQ); *ns*   **Criterion validity (offspring outcomes):**   - **(1) -** Concurrent: fathers’ attachment to the infant → infant-attachment security (12-months); medium *es* - **(1) -** Concurrent: pleasure in interaction (sub-scale) → infant attachment security (12-months); medium *es* - **(0) –** Concurrent: absence of hostility and quality of interaction (sub-scales) → infant attachment security (12-months); *ns* | | |
|  |  |  |  |  |  |  | |
| **Parental responsibility Scale (PRS; McBride & Mills, 1993) (adapted version)**  ----------------------------------  *Initial target population*  Tool initially developed in a sample of mothers and fathers reporting on their preschool age children  *Training and availability:*   - Published article describing the tool (McBride & Mills, 1993) - Self-guided training | **Brown, Mangelsdorf & Neff (2012)** | *Internal consistency*  - *Cronbach alpha:*  α = 0.77, for the overall measure in fathers  *Inter-scale item / domain correlations:* n/r  *Factor analysis:* n/r  *Test-retest reliability:*  n/r | *Content validity:*   - Reviewed from source (McBride & Mills, 1993)   *Theory:* n/r  *Expert review:* n/r  *Content analysis with target group:* n/r | *Response process:*   - Reviewed from source (McBride & Mills, 1993): n/r | **Criterion validity (offspring outcomes):**   - **(0) -** Concurrent: fathers’ responsibility in child care related activities → securely *vs.* insecurely attached infants   (13-months); *ns*  **Convergent validity (another measure of paternal parenting):**   - **(0)** *–* Paternal responsibility in child care activities (PRS) → paternal sensitivity (*via* the Competing demands task (Smith & Pederson, 1988) (13-months); *ns* - **(0)** *–* Paternal responsibility in child care activities (PRS) (13-months) → father-infant attachment (*via* the AQS) (3-years); *ns* | | |
|  |  |  |  |  |  |  | |
|  | **Fuertes et al., (2016)** | *Internal consistency*  - *Cronbach alpha:*  α = > 0.70 for mothers and fathers in each involvement category  *Inter-scale item / domain correlations:* n/r  *Factor analysis:*   - Yes, but no specific details reported   *Test-retest reliability:*   - Non-significant associations between involvement categories assessed at 12-months and 18-months | ↑ | ↑ | **Criterion validity (offspring outcomes):**   - **(1) -** Concurrent: fathers’ involvement in play activities and primary care → infant secure attachment (12, 18-months); es n/r - **(0) -** Concurrent: fathers’ involvement in health care → infant secure attachment (12, 18-months); *ns* | | |
|  |  |  |  |  |  |  | |
|  |  |  |  |  |  | | |
| **Unnamed tool (Barnett & Baruch, 1987)**  -----------------------------------  *Initial target population*  Tool initially developed in a community sample of mothers and fathers reporting on their children in kindergarten  *Training and availability:*   - Published article describing the tool (Barnett & Baruch, 1987) - Self-guided training | **Barry et al. (2011)** | *Internal consistency*   - *Cronbach alpha:*   α = 0.72 for the overall measure (1-month)  *a* = 0.80 for the overall measure (12-months)  *Test re-test reliability*   - Paternal involvement scores (1-month) → (12-months) (*F* (1, 1.29) = 29.47, *p* < .001)   *Inter-scale item / domain correlations:* n/r  *Factor analysis:* n/r | *Content validity*   - Reviewed from source (Barnett & Baruch, 1987)   *Theory*: n//r  *Expert review:* n/r  *Feedback:* n/r  *Content analysis with target group:* n/r | *Response process*   - Reviewed from source (Barnett & Baruch, 1987): n/r - Reviewed from Barry et al. (2011): n/r | **Convergent validity (another measure of paternal parenting):**   - **(1) -** Paternal involvement in childcare activities → paternal perceived parenting skill (*via.* the same tool, but fathers additionally report on their level of skill for each involvement activity); small *es* (1-month)*;* medium *es* (12-months) - **(0)** - Paternal involvement in childcare activities (1-month) → paternal perceived parenting skill (12-months); *ns*   **Convergent validity (severity of psychological symptoms):**   - **(0)** Paternal depressive symptoms: → paternal involvement in childcare activities (1 and 12-months); *ns*   **Discriminant validity (parent symptom level groups):**   - **(0) -** Paternal depressive symptom level groups**:** higher (CES-D > 16) *vs.* lower (CED-D < 16) symptoms → paternal involvement in childcare activities (1 and 12-months); *ns* | | |
|  |  |  |  |  |  |  | |
| **Unnamed tool (****Goodman et al., 2014)**  *Note,* tool devised by the authors (Goodman et al., 2014) by combining two published tools: The Parental Responsibility Scale (PRS; McBride & Mills, 1993) and the Child Care Activity Questionnaire (Montague & Walker-Andrews, 2002)  -----------------------------------  *Initial target population*  Tool initially developed in a sample of mothers with diagnosed depression, and their partners reporting on their infants at 3-12 months  *Training and availability:*   - Published article describing tool (Goodman et al., 2014) - Self-guided training | **Goodman et al. (2014)** | *Internal consistency*   - *Cronbach alpha:*   α = 0.71 for the overall measure (3-month)  α = 0.79 for the overall measure (6-month)  α = 0.83 for the overall measure (12-month)  *Test-retest reliability:*   - Paternal responsibility scores at 3-months → 6-months (*r* = .63) → 12-months *(r* = .65)   *Inter-scale item / domain correlations:* n/r  *Factor analysis:* n/r | *Content validity*~~:~~   - Reviewed from Goodman et al. (2014):   *Theory:* n/r  *Expert review:* n/r  *Content analysis with target group:* n/r | *Response process:*   - Reviewed from Goodman et al. (2014): n/r | **Convergent validity (another measure of paternal parenting):**  **(1)** Paternal responsibility (unnamed tool, Goodman et al., 2014) → paternal weekday engagement (*via.* the CDS, Hofferth et al., 1997) at 3, 6 and 12-months, and weekend engagement at 3 and 12-months; medium *es*  **(1)** Paternal responsibility → paternal weekday accessibility (*via.* the CDS, Hofferth et al., 1997), at 6 and 12-months; medium *es*  **(0)** Paternal responsibility → paternal weekend accessibility (*via.* the CDS, Hofferth et al., 1997), at 3, 6 and 12-months; *ns*  **Convergent validity (severity of psychological symptoms):**  **(0) -** Maternal depressive symptoms**:** → paternal responsibility in childcare activities (birth through 3-months, 3- through 6-months, 6- through 12-months); *ns*  **Discriminant validity (parent clinical diagnostic groups):**  **(0) -** Paternal clinical diagnostic groups**:** presence *vs.* absence of a history of depression/anxiety → paternal responsibility in childcare activities (3, 6, 12-months); *ns*  **(0) -** Maternal clinical diagnostic groups**:** presence *vs.* absence of a history of depression/anxiety → paternal responsibility in childcare activities (3, 6, 12-months); *ns* | | |
|  |  |  |  |  |  |  | |
| **Unnamed tool (Nugent, 1991)**  Analyses of father involvement in relation to offspring outcomes was examined using a combination of summary scores derived from interview data and items from the Father Caretaking Inventory (Nugent, 1987) at 1 and 12-months (Unnamed, Nugent, 1991)  ---------------------------------  *Initial target population*  Tool described in a published article including fathers reporting on their 1-12 months old infants  *Training and availability*   - Published article describing tool (Nugent, 1991)   -Self-guided training | **Nugent (1991)** | *Test-retest reliability:*   - Paternal involvement scores at 1-month → 12-months (*r* = 0.47, *p* < 0.05)   *Internal consistency:* n/r  *Inter-scale item / domain correlations:* n/r  *Factor analysis:* n/r | *Content validity*~~:~~   - Reviewed from source (Nugent, 1991): - *Theory:* evidence of theory driven items specific to paternal parenting   *Expert review:* n/r  *Content analysis with target group:* n/r | *Response process:*   - Reviewed from source (Nugent et al., 1991): n/r | **Criterion validity (offspring outcomes):**   - **(1) -** Predictive: paternal involvement in childcare activities (over the first 12 months) → Infant Bayley Mental Development Index (12-months); small *es* - **(0) -** Predictive: paternal involvement in childcare activities (over the first 12 months) → Infant Bayley Motor Development Index (12-months); *ns* | | |
|  |  |  |  |  |  |  | |
| **Unnamed tool (Paulson et al., 2006)**  -----------------------------------  *Initial target population*  Tool described in a publication including a community sample of mothers and fathers, reporting on their 9-month old infants  *Training and availability*   - Published article describing tool (Paulson et al., 2006)   -Self-guided training | **Paulson et al. (2006)** | *Factor analysis*  -Two factors identified  *Internal consistency:* n/r  *Inter-scale item / domain correlations:* n/r  *Test-retest reliability:*  n/r | *Content validity*~~:~~   - Reviewed from source (Paulson et al., 2006):   *Theory:* n/r  *Expert review:* n/r  *Content analysis with target group:* n/r | *Response process*   - Reviewed from source (Paulson et al., 2006): n/r | **Discriminant validity (parent symptom level groups):**   - **(1) -** Paternal depressive symptom level groups: higher *vs.* lower symptoms (CES-D cut-off) → lower paternal reported engagement in play activities (i.e. playing outside with their child) (9-months) - **(0) -** Paternal depressive symptom level groups: → paternal reported engagement in enrichment activities (i.e., reading, telling stories, singing) and other play activities (i.e. playing games, tickling child, running errands with their child) (9-months) - **(1) –** Maternal depressive symptom level groups: higher *vs.* lower symptoms (CES-D cut-off) → lower paternal reported engagement in play activities (i.e., singing with their child) (9-months) - **(0) -** Maternal depressive symptom level groups: → paternal reported engagement in other enrichment activities and play activities (9-months)   **Convergent validity (severity of psychological symptoms):**   - **(1) -** Paternal depressive symptoms: → paternal positive engagement in enrichment activities (9-months); small *es* - **(0) -** Paternal depressive symptoms: → paternal engagement in play activities (9-months); *ns* - **(0) -** Maternal depressive symptoms: → paternal engagement in enrichment or play activities (9-months); *ns* | | |
|  |  |  |  |  |  | | |
| **Unnamed measure (Ragni et al., 2019)**  (Ad-hoc item attached to the  BISQ; Sadeh et al., 2009)  -----------------------------------  *Initial target population*  Measure described in a publication including a community sample of mothers and fathers reporting on their 8-12-month old infants  *Training and availability*   - Published article describing measure (Ragni et al., 2019)   -Self-guided training | **Ragni et al. (2019)** | *Internal consistency:* n/r  *Inter-scale item / domain correlations:* n/r  *Factor analysis:* n/r  *Test-retest reliability:*  n/r | *Content validity*: n/r  *Theory:* n/r  *Expert review:* n/r  *Content analysis with target group:* n/r | *Response process*   - Reviewed from source (Ragni et al., 2019): n/r | **Convergent validity (severity of psychological symptoms):**   - **(0) -** Paternal affective disorders (self-report): → paternal involvement in bedtime child care (8-12 months); *ns* - **(0) -** Maternal affective disorders (self-report): → paternal involvement in bedtime child care (8-12 months); *ns*   **Criterion validity (offspring outcomes):**   - **(1) -** Concurrent: paternal involvement in bedtime child care (8- 12 months) → infant bedtime difficulties; medium *es* - **(0) -** Concurrent: paternal involvement in bedtime child care (8- 12 months) → child negative emotionality; *ns* | | |
|  | | | | | | | |
| 1 = significant association (*p* < 0.05), 0 = non-significant association (*p* > 0.05); n/r = not reported  ^a^ Details of initial target population and content validity reviewed and extracted from initial source of tool (where available) and/or the related publication(s)  ^b^ Internal structure and relations with other variables extracted from related publication(s)  ^c^ Response process reviewed and extracted from initial source of tool (where available) and/or related publication(s) | | | | | | | |

.
