# Supplementary material for: The Applicability and Performance of Tools Used to Assess the Father-Offspring Relationship in Relation to Parental Psychopathology and Offspring Outcomes
Source: Front Psychiatry. 2021 Jan 5;11:596857. doi: 10.3389/fpsyt.2020.596857 (PMC7814871; doi:10.3389/fpsyt.2020.596857)
Supplement: Supplementary file 9 [file Table_9.docx]

| **Supplementary Materials_Table 9**  Validity evidence supporting interview tools (*n* = 4) used to assess the father-infant relationship - including relationship quality and father involvement. | | | | | | |
| --- | --- | --- | --- | --- | --- | --- |
| **Interview tool / Target population^a^ / Training** | **Related publication(s)** | **Internal structure^b^** | **Content validity^a^** | **Response process^c^** | **Relations with other variables**  Construct, Discriminant and Criterion Validity  *(1) = significant association; (0) = non-significant association* | |
|  |  |  |  |  |  | |
| **Interview tools developed in parental samples (*n* = 4)** | | | | | | |
|  |  |  |  |  |  | |
| **Child Development Supplement to Panel Study of Income Dynamics Time Diary (CDS; Hofferth et al. 1997)**  ----------------------------------  *Initial target population*  Tool is based on survey data gathered from mothers and fathers reporting on their children under 12-years  *Training and availability:*   - Interview manual freely available - 5-day workshop available from the developers | **Goodman et al. (2014)** | *- Inter-scale item / domain correlations:* n/r  *-Inter-rater reliability:*  n/r  *-Factor analysis:* n/r  *-Test-retest reliability:* n/r | *Content validity*  Reviewed from source (Hofferth, 1997): n/r  *Theory:* n/r  *Expert evaluation:* n/r | *Response process*  Reviewed from source (Hofferth, 1997): n/r | **Convergent validity (severity of psychological symptoms):**   - **(1) -** Maternal depressive symptoms**:** → paternal weekend engagement in child care activities (concurrently, 12-months); medium *es* - **(0) -** Maternal depressive symptoms**:** → paternal weekday engagement or paternal accessibility in child and child care activities (concurrently, 3, 6 and 12-months); *ns*   **Discriminant validity (clinical diagnostic groups):**   - **(0) -** Maternal clinical diagnostic groups**:** lifetime history of depression *vs.* no history → paternal engagement or accessibility in child or child care activities (3, 6, 12-months); *ns.* - **(1) -** Paternal clinical diagnostic groups**:** lifetime history of depression *vs.* no history → paternal accessibility in child and child care activities (12-months) (*coefficients n/r* – paternal history examined as potential confounding variable) | |
|  |  |  |  |  |  | |
|  |  |  |  |  |  | |
| **Parental Involvement** **Time Diary (Jia et al. 2016) (modelled on the American Time Use Survey)**  ----------------------------------  *Initial target population*  Tool developed in a community sample of mothers and fathers reporting on their infants from 3-9 months  *Training and availability:*  -Published article describing  tool (Jia et al., 2016)  -Self-guided training | **Jia et al. (2016)** | *-Inter- rater reliability:*  n/r  *- Inter-scale item / domain correlations:* n/r  *-Factor analysis:* n/r  *-Test-retest reliability:*  n/r | *Content validity*  Reviewed from source (Jia et al., 2016): n/r  *Theory:* n/r  *Expert review:* n/r | *Response process*  Reviewed from source (Jia et al., 2016): n/r | **Convergent validity (severity of psychological symptoms):**   - **(1) -** Paternal postnatal anxiety symptoms: → paternal workday engagement in physical child care (through 3 to 9-months); small *es* - **(0) -** Paternal antenatal dysphoria symptoms: → non-workday engagement in enrichment activities (through 3 to 9-months); moderate *es* - **(1) -** Maternal antenatal anxiety symptoms: → paternal non-workday engagement in enrichment activities and physical care (through 3 to 9-months); small *es* - **(0) -** Maternal dysphoria symptoms: → paternal workday and non-workday engagement in physical child care and engagement in child activities (through 3 to 9-months); *ns* | |
|  |  |  |  |  |  |  |
| **Working Model of Child Interview (WMCI; Zeanah et al., 1986)**  ----------------------------------  *Initial target population*  Tool developed in caregivers reporting on their subjective experience and perceptions of childcare and their child.  *Training and availability:*   - Interview manual freely available   2-day training course available from the developers | **Hall et al. (2014)** | *-Inter-rater reliability*  On a sub-sample of 10%, **ϰ** = 0.83 – 0.95 for the overall measure in fathers  *- Inter-scale item / domain correlations:* n/r  *-Factor analysis:* n/r  *-Test-retest reliability:* n/r | *Content validity*  Reviewed from source (Zeanah et al., 1986):  *Theory:* n/r  *-Expert review*:  n/r | *Response process*  Reviewed from source (Zeanah et al., 1986): n/r | **Convergent validity (another measure of paternal parenting):**   - **(1) -**paternal attachment representations (WMCI) → paternal sensitivity (*via.* the NICHD); large *es*   **Criterion validity (offspring outcomes):**   - **(0) –** Predictive: overall paternal attachment representations (6-months) → infant scores in vocabulary (24-months); *ns* | |
|  |  |  |  |  |  |  |
| **Unnamed tool (Frodi et al., 1983)**  ----------------------------------  *Initial target population*  Tool initially developed in a community sample of mothers and fathers reporting on their infants at 5-months  *Training and availability:*   - Tool described in a published article (Frodi et al., 1983) - Self-guided training | **Frodi et al. (1983)** | *-Inter- rater reliability:*  n/r  *- Inter-scale item / domain correlations:* n/r  *-Factor analysis:* n/r  *-Test-retest reliability:* n/r | *Content validity*  Reviewed from source (Frodi et al., 1983):   - *Theory:* evidence of theory driven items specific to paternal parenting   -*Expert review*:  n/r | *Response process*  Reviewed from source (Frodi et al., 1983): n/r | **Criterion validity (offspring outcomes):**   - **(0) –** Predictive: – paternal involvement in child care activities (5-months) → infant-attachment security (11 and 13-months); *ns* | |
| 1 = significant association (*p* < 0.05), 0 = non-significant association (p > 0.05); n/r = not reported; *es* = effect size; ns = non-significant  a Details of initial target population and content validity reviewed and extracted from initial source of tool (where available) or the related publication(s)  b Internal structure and relations with other variables extracted from related publication(s)  c Response process reviewed and extracted from initial source of tool (where available) and/or the related publication | | | | | | |

.
