# Supplementary material for: The Applicability and Performance of Tools Used to Assess the Father-Offspring Relationship in Relation to Parental Psychopathology and Offspring Outcomes
Source: Front Psychiatry. 2021 Jan 5;11:596857. doi: 10.3389/fpsyt.2020.596857 (PMC7814871; doi:10.3389/fpsyt.2020.596857)
Supplement: Supplementary file 10 [file Data_Sheet_1.docx]

| **Supplementary Materials_Search Strategy** | | |
| --- | --- | --- |
| **PsycINFO (Ovid)** | | |
| 1 exp FATHERS/ (10352) | 29 pregnan*.ti,ab. (43499) | 58 ocd.ti,ab. (9955) |
| 2 dad*.ti,ab. (3051) | 30 Prenatal Care/ (1720) | 59 (obsessive adj2 compulsive*).ti,ab. (19017) |
| 3 father*.ti,ab. (44854) | 31 Postnatal Period/ (4237) | 60 (anxiety or anxious).ti,ab. (186294) |
| 4 exp PARENTS/ (87077) | 32 prenatal.ti,ab. (17142) | 61 exp Phobias/ (12633) |
| 5 paternal*.ti,ab. (11065) | 33 pre-natal.ti,ab. (229) | 62 affective disorder*.ti,ab. (16142) |
| 6 couple*.tw. (60125) | 34 postnatal.ti,ab. (18705) | 63 Mental Disorders/ (80186) |
| 7 exp PARENTING/ (91683) | 35 post-natal.ti,ab. (1023) | 64 Adjustment Disorders/ (681) |
| 8 exp PARENTS/ (87077) | 36 postpartum.ti,ab. (10731) | 65 dysthymic disorder.ti,ab. (964) |
| 9 men.ti,ab. (154402) | 37 post-partum.ti,ab. (1132) | 66 exp STRESS/ (102666) |
| 10 parent*.tw. (263192) | 38 puerperal.ti,ab. (481) | 67 Post-Traumatic/ or posttraumatic stress disorder/ (30755) |
| 11 mens.ti. (3833) | 39 ante-natal.ti,ab. (53) | 68 blues.ti,ab. (801) |
| 12 male*.ti. (39535) | 40 antenatal.ti,ab. (3203) | 69 Mental Health/ (59717) |
| 13 partner*.tw. (98728) | 41 antepartum.ti,ab. (313) | 70 melancholia.tw. (2474) |
| 14 spous*.ti,ab. (20508) | 42 ante-partum.ti,ab. (12) | 71 distress*.ti,ab. (66903) |
| 15 "co-mother*".tw. (28) | 43 postnatal period/ (4237) | 72 PTSD.tw. (30944) |
| 16 (LGBT or lesbian* or gay* or homosexual* or queer* or bisexual* or transgender*).tw. (42418) | 44 peri-partum.ti,ab. (10) | 73 ("post traumatic stress" or posttraumatic).tw. (42277) |
| 17 GRANDPARENTS/ (2449) | 45 peripartum.ti,ab. (282) | 74 exp Postpartum Depression/ (4365) |
| 18 SIBLINGS/ (6465) | 46 perinatal.ti,ab. (9518) | 75 pnd.ti,ab. (1348) |
| 19 SPOUSES/ (11449) | 47 peri-natal.ti,ab. (65) | 76 ppd.ti,ab. (1033) |
| 20 exp Parent Child Relations/ (65696) | 48 Parturition.tw. (1270) | 77 (trauma* adj3 birth).ti,ab. (488) |
| 21 (sibling* or relative* or brother* or sister* or grandparent* or grandmother* or grandfather* or grandad* or grandpa* or granny or grannies or grandma*).tw. (304785) | 49 childbirth.ti,ab. (4563) | 78 (psychosocial* or psychological*).ti. (92882) |
| 22 (wive* or wife or girlfriend* or boyfriend* or husband*).tw. (21465) | 50 childbearing.ti,ab. (3502) | 79 ((wellbeing or engag* or psychosocial* or psychological*) adj2 (partner* or father* or parent* or spouse* or husband*)).tw. (2864) |
| 23 coparent*.tw. (915) | 51 Birth/ (7498) | 80 (co-parent* adj2 (quality or skills or educat*)).tw. (41) |
| 24 "co parent*".tw. (598) | 52 or/28-51 [postpartum period] (86331) | 81 co-parent*.ti. (140) |
| 25 Homosexuality/ (7443) | 53 exp MAJOR DEPRESSION/ (122093) | 82 (relationship* adj3 (infant* or child* or baby or toddler* or newborn*)).tw. (27525) |
| 26 ("same sex" adj2 couple*).tw. (973) | 54 depress*.ti,ab. (282461) | 83 Father Child Relations/ (4570) |
| 27 or/1-26 [parents] (892969) | 55 ANXIETY/ (57662) | 84 exp Parent Child Relations/ (65696) |
| 28 pregnancy/ (21033) | 56 ANXIETY DISORDERS/ (17464) | 85 or/53-84 (789973) |
|  | 57 Obsessive Compulsive Disorder/ (13274) | 86 27 and 52 and 85 (13184) |
